# What does an AI-generated “cancer survivor” look like? An analysis of images generated by text-to-image tools

**DOI:** 10.1007/s11764-025-01760-1

**Published:** 2025-03-01

**Authors:** Nicole Senft Everson, Anna Gaysynsky, Irina A. Iles, Kristin E. Schrader, Wen-Ying Sylvia Chou

**Affiliations:** 1 Health Communication and Informatics Research Branch, Behavioral Research Program, Division of Cancer Control and Population Sciences, National Cancer Institute, Rockville, MD USA; 2 ICF Next, ICF, Rockville, MD USA; 3 Westat, Rockville, MD USA

**Keywords:** Cancer survivors, Generative artificial intelligence, AI-generated images, Algorithmic bias, Representational harm

## Abstract

**Purpose:**

Cancer survivorship begins at diagnosis and encompasses a wide variety of experiences, yet prominent societal narratives of survivorship emphasize a positive, post-treatment “return-to-normal.” These representations shape how survivorship is understood and experienced by cancer survivors and the public. This study aimed to (1) characterize artificial intelligence (AI)–generated images of cancer survivors and (2) compare them to images of cancer patients to understand how these images might reflect and amplify prevalent survivorship narratives.

**Methods:**

Two AI text-to-image tools (*DALL-E*, *Stable Diffusion*) were prompted to generate 40 images each of cancer survivors and cancer patients (*n* = 160 images). Images were coded for perceived demographics, affect, health, markers of illness or cancer, and setting. Chi-square analyses tested differences between images of cancer patients and survivors. Quantitative data were complemented by coders’ qualitative insights.

**Results:**

Cancer survivors in AI-generated images were largely perceived as White (80%), feminine (80%), young (51%), happy (69%), and healthy (80%), and many images were observed to conform to Western beauty ideals. Pink (64%), cancer ribbons (35%), and head scarves (51%) were prominent visual features in survivor images. Compared to images of cancer patients, survivor images more frequently featured individuals perceived as non-White (*p* = .03), young (*p* < .001), affectively positive (*p* < .001), and healthy (*p* < .001), and less frequently included markers of illness like portraying individuals in bed (*p* < .001) or in medical settings (*p* < .001).

**Conclusions:**

AI-generated images of cancer survivors fail to reflect the breadth of survivor demographics or experience.

**Implications for Cancer Survivors:**

AI-generated images may perpetuate narrow views of cancer survivorship.

**Supplementary Information:**

The online version contains supplementary material available at 10.1007/s11764-025-01760-1.

## Introduction

Recent advances in machine learning methods have enabled the development of sophisticated artificial intelligence (AI) text-to-image generators that can convert text prompts into high-quality, contextually relevant visual outputs [1]. These tools offer health researchers and practitioners the ability to create engaging, realistic, and customized images quickly and at low cost [2]. In the context of health, AI text-to-image tools might be used to develop patient-facing education materials [1], generate “stock” images for use in promotional materials for health organizations [3], create medical illustrations to support medical education [4], and make behavioral interventions more engaging and effective [5]. However, because AI tools are trained on text and images scraped from the Internet, they are susceptible to replicating biases contained in those data [1, 6]. Prior research has demonstrated that AI-generated images may amplify biases and stereotypes [1, 7]—for example, research shows that these models overwhelmingly depict surgeons as White and male [1] and may associate certain negative attributes, such as “poor,” with darker skin tones [8]. However, beyond simply reflecting existing biases, there is also concern that once AI-generated images circulate more widely in society, they may further entrench biases and start to shape social perceptions. To date, evaluations of generative AI tools in the context of health have mostly focused on large language models [9], whereas health-related images produced by text-to-image tools have received relatively little attention [2]. The expanding availability and increasing uptake of AI text-to-image generators suggests an urgent need to understand the characteristics of the images generated by these tools and how these images may influence health-related cultural narratives and biases.

The way health conditions are portrayed in culturally available images can reflect, shape, or even challenge how individuals with these conditions are viewed in society—with implications for the way they are treated as well as their own sense of identity [7]. A recent study of dementia-related images generated by *Stable Diffusion*, an AI image generator, found that the images overrepresented light-skinned individuals and featured visual tropes that could reinforce harmful stereotypes (e.g., dementia as a “living death”) [7]. As AI-text-to-image tools are increasingly used in industries that shape cultural norms (such as entertainment and marketing) [10], problematic and biased images could lead to the reinforcement of stereotypes and the erasure of alternate narratives [11].

Cultural discourses related to cancer provide models through which survivors make sense of their experiences [12]. These narratives shape how cancer is collectively understood as well as individually experienced [13]. The National Cancer Institute states that an “individual is considered a cancer survivor from the time of diagnosis, through the balance of life,” and includes both those living with cancer and those free of cancer [14]. Despite this broad definition, certain accounts of survivorship may be privileged over others in popular media. For example, a content analysis of articles about cancer survivors in leading national daily newspapers found that cancer is often portrayed as a positive life event [13]. Similarly, prior research has shown that media portrayals of childhood cancer rarely show children affected by cancer as anything other than brave and uncomplaining [13]. Although inspiring and hopeful portrayals of survivorship can have positive impacts (e.g., decreasing stigma associated with a cancer diagnosis), an idealized and narrow survivorship discourse could also have negative consequences. For example, it could make those whose experiences do not fit within this narrative feel excluded and provide an inaccurate view of the cancer experience and of cancer survivors [13]. Idealized narratives of cancer survivorship could also compel survivors to “self-censor” in an attempt to conform to social expectations about how a cancer survivor should feel or behave [13]. These dominant narratives may legitimize a certain set of experiences, encouraging conformity to practices that align with them while discrediting divergent perspectives [15].

Breast cancer survivorship is portrayed in the media more often than other cancer types [16], and breast cancer survivors are often represented as triumphant, happy, and healthy—portrayals that have been criticized for suppressing the less palatable images of the cancer experience and potentially alienating individuals who experience ongoing effects of cancer, have a poor prognosis, or otherwise struggle with the impacts of cancer and its treatment [17]. These narratives may limit ways of thinking about cancer. Furthermore, these narratives may not serve members of groups who draw on different cultural frameworks and lived experiences to understand their cancer survivorship [17]. For example, Chelsey Hauge, a cancer survivor and scholar, notes that normative breast cancer narratives did not resonate with her as a queer young adult with breast cancer [18]. Some Black prostate cancer survivors similarly describe struggling with how their treatment impacts culturally shaped views of masculinity [19], urging broader representation of men with cancer and attention to the varied ways masculinity may be represented in images of cancer survivorship.

Although the potential for AI models to produce socially biased output is widely recognized, empirical investigation into AI-generated images—especially in the context of health—remains limited [20]. Given the potential of these generated images to perpetuate or amplify stereotypes on a large scale, and perhaps even corrupt future generations of technologies if these AI-generated images become part of datasets used to train new models, it is critical to assess their embedded biases [7, 20, 21]. In the current study, our primary aim was to characterize outputs produced by two leading AI image generators (*DALL-E* and *Stable Diffusion*) when prompted to create images of cancer survivors, in order to offer insights into the ways cancer survivors are depicted in generated images and gauge the utility of using these images for real-world applications. Additionally, over the past few decades, the terminology used for individuals with a cancer diagnosis has shifted from terms like “victim” or “patient” to the currently preferred term “survivor” [22]. Some research has demonstrated differences in psychosocial outcomes between people who endorse these different terms [22], suggesting these terms carry distinct connotations that may have a real-world impact. Therefore, a secondary, exploratory aim of the analysis was to compare image outputs generated in response to prompts using the term “cancer survivor” to those generated by the term “cancer patient” to assess whether the use of different terms produces differences in image features. We explored whether images of cancer survivors would reflect the overrepresentation of breast cancer survivorship relative to other cancers in popular media and would emphasize positivity and healthfulness (relative to the experience portrayed in cancer patient images).

## Methods

### Sample generation

We used *Stable Diffusion* and *DALL-E 3* to generate 40 images per tool for each of the following prompts: (1) “[a photograph of a] cancer survivor” and (2) “[a photograph of a] cancer patient,” for a total of 160 images (see Online Resource 1). This sample size is in line with previous analyses of AI-generated images (e.g., 6, 8, 21). Several rounds of pilot testing to refine the prompts prior to main sample generation led to the addition of the phrase “a photograph of” to prompts for *DALL-E 3* to obtain images that were more comparable to those generated with *Stable Diffusion’s* default style setting. Additionally, *DALL-E 3* was queried through the ChatGPT interface, and explicit instructions not to alter the prompt (“do not make any modifications or additions to the prompt”) were added to prevent the tool from editing the prompt and to maximize consistency in how both tools generated their output (see Online Resource 2 for specific prompt wording and selected settings, where applicable, for each AI tool). The images from both tools were generated over a 2-week period (3/21/2024–4/2/2024).

### Image coding

A codebook informed by prior research on visual representations of individuals with cancer [23–25], other studies of AI-generated images [7, 26], and key image features identified during the prompt refinement process was iteratively developed by the research team (the full codebook is provided in Online Resource 3). Codes included perceived demographic characteristics of individuals depicted in the images (e.g., race, age, gender), affect, overall health appearance, markers of illness (i.e., being in bed, dermatological issues, wearing medical clothes, being in a clinical setting, presence of medical staff or equipment), and inclusion of common visual markers of cancer (i.e., baldness, head scarves, cancer ribbons, the color pink). The codebook was further refined through an initial coding of 20% of images by all members of the research team. All images were then coded using a *Qualtrics* (Qualtrics, Provo, UT) survey form built from the final codebook. Images were randomly assigned to pairs of coders who were blinded to the prompt and AI tool that generated the image. Given that coding entails some degree of subjective judgement, extensive coder training and pilot testing prior to coding the final dataset were conducted to ensure that codes were applied correctly and consistently. Any disagreements between the two initial coders were adjudicated by a third member of the team.

### Analysis

For quantitative analysis, several codes, including health appearance and affect, were converted from 5- to 3-point scales (e.g., combining “slightly negative” and “negative” as well as “slightly positive” and “positive” affect), and two response categories in the “setting” code were collapsed. Summary statistics (frequencies and percentages) were calculated separately for each prompt (“cancer survivor” and “cancer patient”). Chi-square tests were then used to determine if there were statistically significant differences (defined as *p* < 0.05) in characteristics between images generated by the two prompts. In cases when any cell included in the comparison had expected frequencies < 5, Fisher’s exact tests were used in place of Chi-square tests [27]. Percentages were compared across prompts to assess the magnitude of statistically significant differences. In addition, we included qualitative insights derived from coders’ observations to further contextualize quantitative findings and highlight additional salient features of AI-generated images of cancer survivors that emerged during the coding process.

## Results

### Characterization of AI-generated images of cancer survivors

**Fig. 1:**
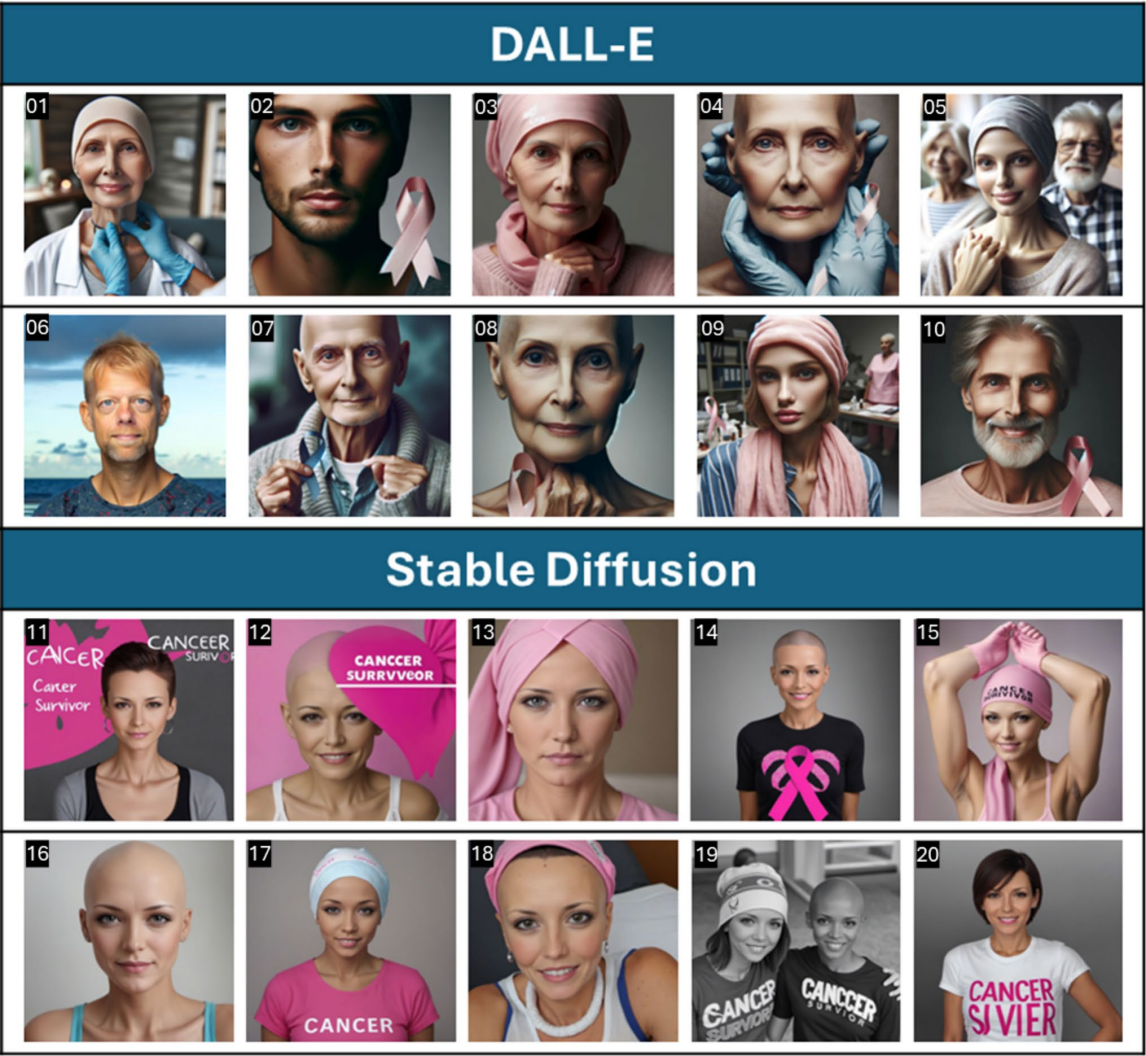
Example images of cancer survivors generated by Dall-E and Stable Diffusion. Each set of images was randomly sampled from the 40 outputs generated using each tool

As shown in Table 1, most cancer survivors portrayed in AI-generated images were perceived as White (80%), feminine (80%), and young (51%). Coders observed that many images depicted people with smooth, unwrinkled skin, indicating youth (see examples of generated images in Fig. 1). Even images that portrayed individuals perceived to be middle-aged or older (e.g., due to gray hair) often depicted them with smooth skin and a youthful, attractive appearance (see Fig. 1, image 8).

**Table 1:** Frequency of image features for each prompt, combined across AI image generation tools, and compared using Chi-square analyses

	Cancer survivor *n* = 80 (%)	Cancer patient *n* = 80 (%)	Chi-square value	*p*-value
Race			6.82	**0.03**
White	64 (80.0)	73 (91.3)		
Non-White	11 (13.8)	2 (2.5)		
Unclear	5 (6.3)	5 (6.3)		
Age			25.68	** < .001**
Child (< 18)	0 (0.0)	0 (0.0)		
Young adult (18–39)	41 (51.3)	15 (18.8)		
Middle-aged (40–65)	15 (18.8)	43 (53.8)		
Older adult (> 65)	24 (30.0)	22 (27.5)		
Gender			0.04	0.84
Masculine	16 (20.0)	14 (17.5)		
Feminine	64 (80.0)	66 (82.5)		
Affect			25.95	** < .001**
Negative	1 (1.3)	12 (15)		
Neutral or mixed	24 (30.0)	43 (53.8)		
Positive	55 (68.8)	25 (31.3)		
Health appearance			56.18	** < .001**
Sick	2 (2.5)	17 (21.3)		
Neutral or can’t tell	14 (17.5)	46 (57.5)		
Healthy	64 (80.0)	17 (21.3)		
Image setting			NA	** < .001**^**a**^
Medical setting	2 (2.5)	18 (22.5)		
Indoor-nonmedical setting	21 (26.3)	29 (36.3)		
Outdoor	5 (6.3)	1 (1.3)		
No identifiable background	52 (65.0)	32 (40.0)		
Medical equipment			16.45	** < .001**
Yes	2 (2.5)	21 (26.3)		
No	78 (97.5)	59 (73.8)		
Medical staff			2.67	0.10
Yes	2 (2.5)	8 (10.0)		
No	78 (97.5)	72 (90.0)		
Medical clothing (e.g., hospital gown)			24.29	** < .001**
Yes	1 (1.3)	25 (31.3)		
No	79 (98.8)	55 (68.8)		
Subject in bed			24.58	** < .001**
Yes	0 (0.0)	23 (28.8)		
No	80 (100.0)	57 (71.3)		
Dermatological symptoms (e.g., rash)			NA	**0.03**^**a**^
Yes	0 (0.0)	6 (7.5)		
No	80 (100.0)	74 (92.5)		
Baldness			0.35	0.55
Yes	18 (22.5)	14 (17.5)		
No	62 (77.5)	66 (82.5)		
Head scarf			1.63	0.20
Yes	41 (51.3)	50 (62.5)		
No	39 (48.8)	30 (37.5)		
Pink color prominent			2.07	0.15
Yes	51 (63.8)	41 (51.3)		
No	29 (36.3)	39 (48.8)		
Cancer ribbon			12.94	** < .001**
Yes	28 (35.0)	8 (10.0)		
No	52 (65.0)	72 (90.0)		

^a^Due to expected values < 5, Fisher’s exact test was used in place of Chi-square

Most individuals in cancer survivor images were perceived as showing positive affect (69%), with coders observing that the positive emotions were typically indicated by smaller or closed-mouth smiles associated with calmness. Coders also perceived other positive feelings, such as strength or determination shown, for example, through a direct gaze. Images also largely portrayed people who appeared to be in good health (80%), indicated by characteristics like luminous skin and straight posture. Few images conveyed medical settings (2.5%) or showed medical equipment, staff, or clothing (≤ 2.5%), and none showed the person in bed or with dermatologic symptoms, all further contributing to an overall sense of the good health of image subjects. Most images had no identifiable background (e.g., showed a neutral backdrop) (65%) or featured a non-medical indoor space (26%), or more rarely were set outdoors (6%).

Over one-third of AI-generated images of cancer survivors included cancer ribbons (35%). Images portraying people who were bald (23%) or wearing head scarves (51%) were also relatively common. Head scarves were frequently shown on individuals both with and without hair, and several images that did show hair depicted very short hair, as if it were growing back following hair loss. The color pink, typically associated with breast cancer, was even more prominent (64%), regardless of the perceived gender of the person in the image.

Several additional salient themes were qualitatively observed during the coding process. Though we did not code for body size, it was notable that all images portrayed people with thin appearances, and none showed excess weight. Coders noted that images largely aligned with Western beauty ideals, with survivors being depicted as young, thin, with flawless skin texture, large eyes, high cheekbones, and often wearing make-up (see Fig. 1, image 9). Although we did not code for the presence of other individuals (excluding medical staff), coders also noted that very few images included more than the focal person (Fig. 1, images 5 and 19 are notable exceptions). Additionally, images generally did not depict subjects engaged in any kind of activity; the subject was usually shown simply standing or sitting and looking straight ahead. These features, along with the frequent use of neutral or otherwise unclear backgrounds, resulted in a general lack of context for the subjects depicted. Coders noted that the images often relied on head scarves, clothing with the label “cancer survivor,” baldness or very short hair, and cancer ribbons to indicate the subject was a cancer survivor, with there being little else to suggest a connection to cancer (see Fig. 1, images 2 and 20).

### Comparison between AI-generated images of cancer survivors vs. cancer patients

AI-generated images of cancer survivors differed significantly from images generated using the prompt “cancer patient” in terms of race (*p* = 0.03), age (*p* < 0.001), affect (*p* < 0.001), health appearance (*p* < 0.001), setting (*p* < 0.001), and some markers of illness and cancer (see Table 1 for results of all comparison tests; see Online Resource 4 for examples of images generated by the “cancer patient” prompt). Examination of proportions across prompts suggests that statistically significant differences reflect large and meaningful differences between the images generated by each prompt. Though all images, across prompts, overwhelmingly depicted people who appeared to be of White race, cancer survivors were more likely to be perceived as non-White compared to cancer patients (14% versus 2.5%). Compared to cancer patients, cancer survivors were also more likely to be perceived as young adults (51% versus 19%), affectively positive (69% versus 31%), and healthy (80% versus 21%). Cancer survivors were less likely to be shown in a medical setting (2.5% versus 23%), in bed (0% versus 29%), with medical equipment (2.5% versus 26%), wearing medical clothes (like hospital gowns) (1.3% versus 31%), or with dermatologic symptoms (0% versus 7.5%). Prevalence of baldness (23% survivors versus 18% patients) and head scarves (51% survivors versus 63% patients) did not significantly differ across prompts. Cancer ribbons were more common in images of cancer survivors than patients (35% versus 10%), though the prevalence of pink was similar in images of cancer patients and cancer survivors (64% survivors versus 51% patients).

## Discussion

This visual content analysis examined images produced by two common AI text-to-image tools when prompted to generate images of a “cancer survivor” and compared these images to those generated using the term “cancer patient” to characterize the way cancer survivors are depicted by generative AI models and assess the potential benefits and pitfalls of using AI-generated images of cancer survivors in real-world applications. Findings reveal a notable lack of heterogeneity in terms of both demographic characteristics and experiences of cancer survivorship, with AI text-to-image tools generating images that portray cancer survivors as predominantly young, White, happy, and healthy. Results of this study suggest that AI-generated images reflect several normative narratives of cancer survivorship and risk reinforcing them if widely deployed.

AI-generated images of cancer survivors illustrate common tropes and visual characteristics used to depict cancer survivorship in popular media. Breast cancer survivorship is the most culturally prominent and widely portrayed cancer type in the media [16], which is perhaps why AI-generated images were predominantly feminine-appearing and why the color pink, associated with breast cancer awareness, was prevalent even in images portraying men and even though our prompt did not specify a cancer type. Consistent with our results, analyses of breast cancer–related images (e.g., in magazines, on social media) have found that people of color were underrepresented [25, 28, 29] and that featured individuals tended to be younger [25, 28].In their study of breast cancer images, Andsager et al. also found that magazine images tended to reinforce stereotypical portrayals of femininity, noting that the social construction of feminine beauty seemed to have been prioritized over other considerations like accuracy [28] or variety in image selection. This is consistent with our qualitative observation that many of the “cancer survivor” images depicted individuals who aligned with Western ideals of feminine beauty. As noted by Samantha King in “Pink Ribbon Inc.,” the feminine ideal is often reflected in mass media images of breast cancer survivors who are often presented as youthful, ultrafeminine, slim, and immaculately groomed [30].

It is important to critically reflect on potential harms if AI text-to-image tools reinforce normative representations of cancer survivorship as relating primarily to young, White, conventionally attractive women. Older individuals and racially minoritized individuals are in fact at higher risk for many types of cancer, and a lack of representation in survivor images of individuals with these characteristics may give the public an inaccurate impression of cancer risk in these populations [28] or create the impression that these groups are less likely than younger, White women to be survivors in the colloquial sense of the word (i.e., to “beat” cancer). Furthermore, a lack of demographically varied AI-generated images of cancer survivors may be an example of erasure—a type of representational harm where certain groups are systematically absent or underrepresented in AI systems, which can lead to their further marginalization [31, 32]. Overrepresentation of breast cancer, relative to other cancer diagnoses, may be another example of representational harm or erasure, as individuals with other types of cancers may not see themselves represented in these images. For example, a qualitative study of cancer survivors found that for women with a history of ovarian cancer, the vision of “life beyond cancer” that is dominant in the discourse around breast cancer survivorship failed to resonate with their own experiences [16]. Similarly, portrayals that consistently align with conventional beauty ideals may provide a narrow model for viewers about what is “acceptable” and “beautiful” for a cancer survivor [15], leaving little room for alternative discourses that expand conventional notions of beauty [15] or acknowledge that cancer and its treatment may alter bodily appearance [25, 28].

In addition to demographic homogeneity, AI-generated images also presented a narrow view of the cancer survivorship experience. Many of the generated “cancer survivor” images in our study portrayed individuals with a healthy appearance, positive affect, and few markers of illness. This contrasted with images generated by the “cancer patient” prompt, which less frequently portrayed individuals who looked healthy or happy, and more often included markers of illness and allusions to active treatment. Many survivor images relied on the inclusion of text stating “cancer survivor” or cancer ribbons to signal a connection to cancer. Relatedly, whereas around one-quarter of “cancer patient” images featured a medical setting or included medical equipment, “cancer survivor” images rarely featured medical settings or showed medical equipment. The overall narrative conveyed by these features of cancer survivor images aligns with dominant cultural discourses that portray survivors as happy, healthy, and whole [17, 33]. These images also seem to reflect the lay understanding of the term “survivor” as an individual who has “beaten” cancer and moved past treatment, rather than the technical definition of the term. AI-generated images seem to exclusively portray survivorship as a “return to normal” after treatment completion, a contrast to the National Cancer Institute’s definition of survivorship as beginning from the time of diagnosis and including those living with cancer [14].

Positive portrayals of cancer survivorship are not inherently problematic—they could be inspiring,reduce stigma, provide hope, encourage feelings of social worth, and facilitate healthy adjustment to a cancer diagnosis [17]. However, the lack of alternative portrayals in AI-generated images is concerning and may alienate survivors who are living with metastatic disease, experience long-term effects of cancer treatment, or have a poor prognosis [17, 33]. Even survivors who are in remission or have a positive prognosis may struggle with emotional, social, financial, or other difficulties related to their cancer or its treatment [33], and exclusively positive images of survivorship could create cultural norms that compel survivors to adopt a relentlessly cheerful performance of self in the face of a cancer diagnosis [12]. Portrayals of the upbeat, grateful, resilient cancer survivor have been criticized for silencing and invalidating emotions such as anger and grief [17] and potentially inducing shame in survivors whose experiences do not conform to these narratives [12]. Ideally, images generated by AI tools would reflect the variety of survivorship experiences, both those that conform to dominant cultural narratives about survivorship and those that acknowledge alternative constructions of life after a cancer diagnosis that are typically invisible in cultural discourses about survivorship.

Another noteworthy finding was that many of the “cancer survivor” images had no identifiable setting, often using neutral backgrounds. In their study of dementia-related AI images, Putland et al. similarly observed that subjects were regularly set against decontextualized backgrounds, following the conventions of “stock images” meant to convey a general example of an idea or category rather than an individual [7]. The observed homogeneity of these images, discussed above, becomes much more concerning if these images are used as stock images and interpreted as generic archetypes of cancer survivors [7]. Cancer survivor images also infrequently depicted multiple individuals, such that the focus was generally on the solitary individual with cancer, removed from any kind of social context. This is another way that cancer survivors were decontextualized in these images, failing to acknowledge the important role of others—including caregivers, friends, family, and care teams—in the cancer survivorship experience. There were a few notable exceptions of images where the focal subject was shown alongside others, which served as a contrast that further highlighted the usefulness of images that emphasize community and social relationships in the survivorship experience.

### Limitations

Although this study provides some important initial insights into how cancer survivors are portrayed in AI-generated images, the analysis has some limitations. First, coding for image features—particularly complex characteristics such as race—is inherently subjective to some degree and misclassification is possible. Steps were taken to improve coding reliability, including extensive coder training, team discussion, and double coding of the final dataset. It should also be noted that this study relied on only two broad prompts to generate 40 images per prompt. It is possible that different prompts or a larger number of images would have revealed different patterns. It is also not clear whether outputs may have been affected by specific user characteristics (e.g., location). Future studies may therefore seek to replicate current findings to see whether identified patterns are consistent across users, prompts, and samples. Additionally, AI models are constantly evolving, and this analysis can only reflect the tools’ outputs at a particular moment in time (March–April 2024). Similarly, this study only assessed two AI models, and results may not generalize to other AI tools that employ different algorithms or training data. Future studies could look at a wider set of AI models and monitor their outputs for change over time. It is also possible that some of the observations highlighted in this study may be an artifact of the defaults of these systems or standard conventions in photography and other types of images AI tools are trained on, rather than being specific to the portrayal of cancer survivors (e.g., AI tools generally tend to generate images of young, attractive women [10]). However, it is important to be aware that these features may show up in AI-generated images of cancer survivors and shape the discourse around survivorship.

### Implications

It is possible that careful prompt engineering could help mitigate some of the biases observed in the present study and increase heterogeneity in the generated images [7]. For example, prompts specifying an “African American cancer survivor” could be used to address the lack of racial diversity, and prompts requesting images of a “cancer survivor in a hospital” could be used to obtain more images of cancer survivors in medical settings. However, the ultimate goal should be for these tools to produce accurate and varied representations without the need for additional specifiers [1], as prompt engineering requires individual users to recognize the issue and commit to spending the time and resources to achieve diverse outputs [8, 11]. Images also necessarily contain details beyond what is specified in the prompt, and these unspecified elements present additional opportunities for bias. For example, the prompt “African American cancer survivor” may increase racial diversity, but may continue to yield images of young, attractive, smiling women [8, 11]. If generative AI systems follow the “Ambiguity In, Diversity Out” principle (i.e., that outputs cover the range of possibilities when a characteristic of the image is under-specified) [8], this would take the burden of identifying and addressing bias off individual users. The use of training data that is more varied and inclusive, in terms of the people and perspectives represented [7], could also help mitigate some of the issues observed.

## Conclusion

Available images of cancer survivors reflect and shape cultural narratives about what cancer is, who gets cancer, and what it means to be a survivor [13, 18]. These cultural narratives can influence how survivors perceive themselves and their experiences as well as how others in society understand them [12, 17, 18]. Images can have a powerful impact on individuals’ attitudes, values, beliefs, and behaviors [28], making it critical to examine the content of AI-generated images depicting cancer survivors and understand the messages they convey about survivorship. The current analysis suggests that AI-generated images of cancer survivors fail to reflect the full breadth of cancer survivorship in terms of both demographics and experience, tending to feature young, White-appearing women and to conform to dominant cultural narratives of cancer survivorship, including successful treatment, return to normalcy, wholeness of appearance, and resilience after cancer [15]. Greater variety in cancer survivor images—both in terms of demographics and experiences—is needed. The homogeneity observed in the current analysis suggests a need for caution in the use of current AI text-to-image models for applications such as the development of patient-facing materials. The study also highlights the need for ongoing research using systematic approaches to analyze cancer-related AI-generated images, as new models are constantly being released, and some of the problematic features of these images are subtle and may only become apparent when seen as a pattern across a larger set of images [7].

## Supplementary Information

Below is the link to the electronic supplementary material.

Supplementary file1 (ZIP 11162 KB)

Supplementary file2 (DOCX 335 KB)

Supplementary file3 (DOCX 17 KB)

Supplementary file4 (DOCX 579 KB)

## Data Availability

Images generated and analyzed in this manuscript are included in online supplements to this manuscript. Additional metadata are available upon request.

## Declarations

### Disclaimer

Opinions expressed by the authors are their own and this material should not be interpreted as representing the official viewpoint of the US Department of Health and Human Services, the National Institutes of Health, or the National Cancer Institute.
