# Supplementary figures and images for: What does an AI-generated “cancer survivor” look like? An analysis of images generated by text-to-image tools (part 2 of 2)
Source: J Cancer Surviv. 2025 Mar 1;20(4):1612–21. doi: 10.1007/s11764-025-01760-1 (PMC13375691; doi:10.1007/s11764-025-01760-1)

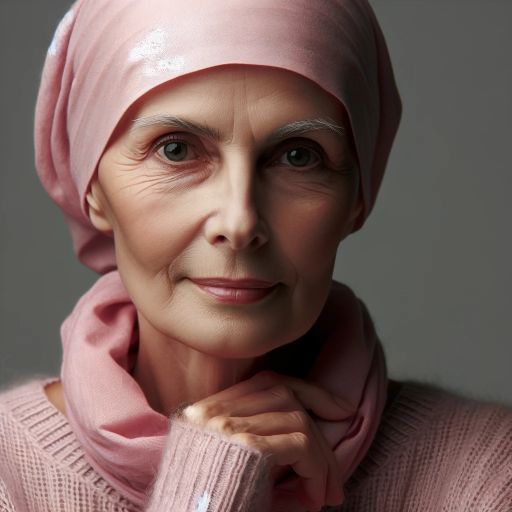

Supplement: Supplementary file 1 — Supplementary file1 (ZIP 11162 KB) [file 11764_2025_1760_MOESM1_ESM.zip › Data Images/cancer survivor/ChatGPT/401.jpg]

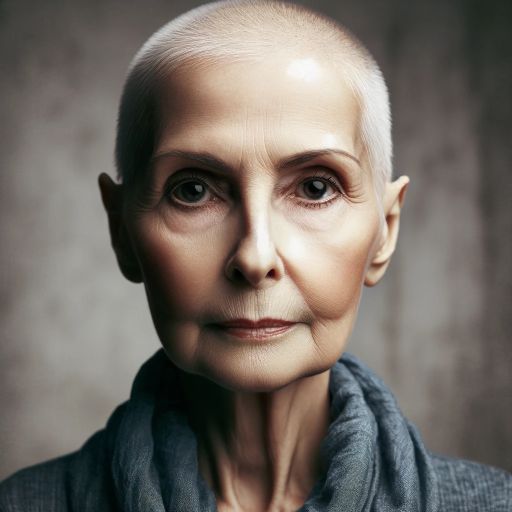

Supplement: Supplementary file 1 — Supplementary file1 (ZIP 11162 KB) [file 11764_2025_1760_MOESM1_ESM.zip › Data Images/cancer survivor/ChatGPT/402.jpg]

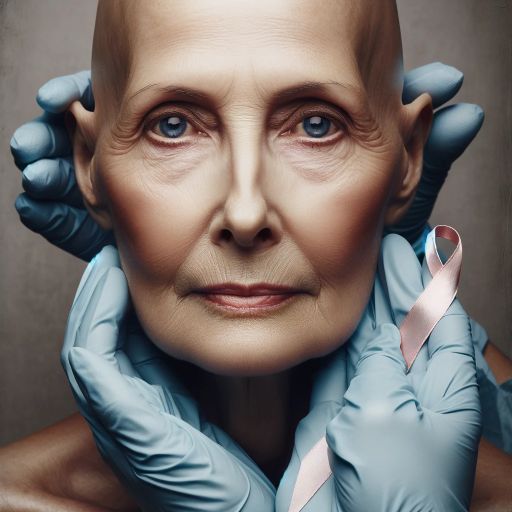

Supplement: Supplementary file 1 — Supplementary file1 (ZIP 11162 KB) [file 11764_2025_1760_MOESM1_ESM.zip › Data Images/cancer survivor/ChatGPT/403.jpg]

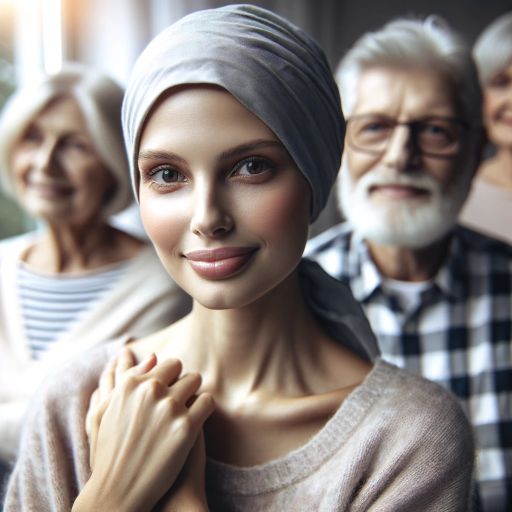

Supplement: Supplementary file 1 — Supplementary file1 (ZIP 11162 KB) [file 11764_2025_1760_MOESM1_ESM.zip › Data Images/cancer survivor/ChatGPT/404.jpg]

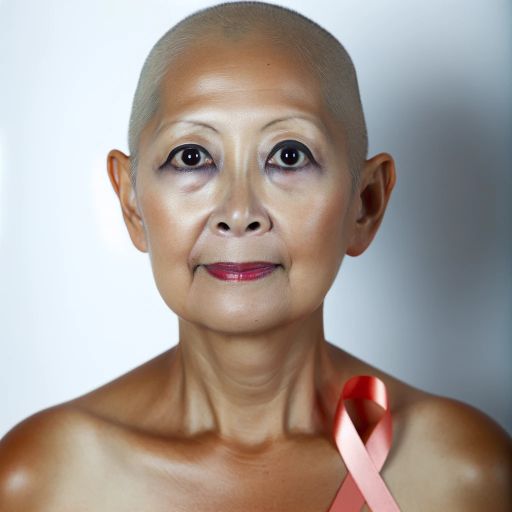

Supplement: Supplementary file 1 — Supplementary file1 (ZIP 11162 KB) [file 11764_2025_1760_MOESM1_ESM.zip › Data Images/cancer survivor/ChatGPT/405.jpg]

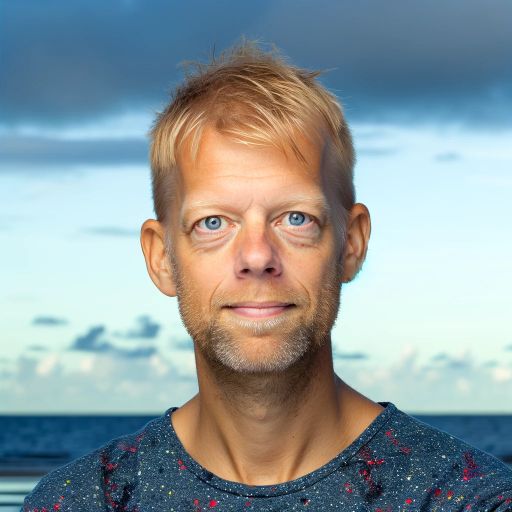

Supplement: Supplementary file 1 — Supplementary file1 (ZIP 11162 KB) [file 11764_2025_1760_MOESM1_ESM.zip › Data Images/cancer survivor/ChatGPT/406.jpg]

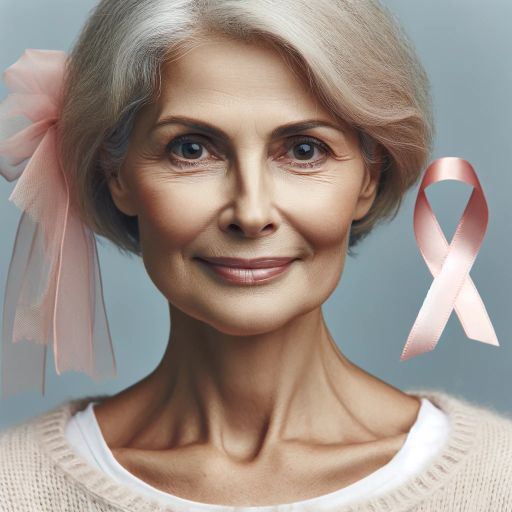

Supplement: Supplementary file 1 — Supplementary file1 (ZIP 11162 KB) [file 11764_2025_1760_MOESM1_ESM.zip › Data Images/cancer survivor/ChatGPT/407.jpg]

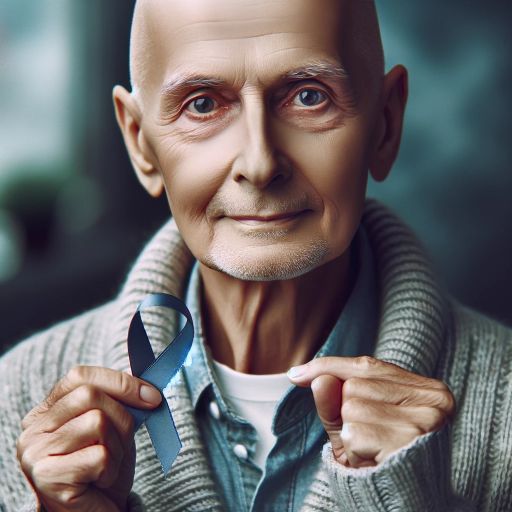

Supplement: Supplementary file 1 — Supplementary file1 (ZIP 11162 KB) [file 11764_2025_1760_MOESM1_ESM.zip › Data Images/cancer survivor/ChatGPT/408.jpg]

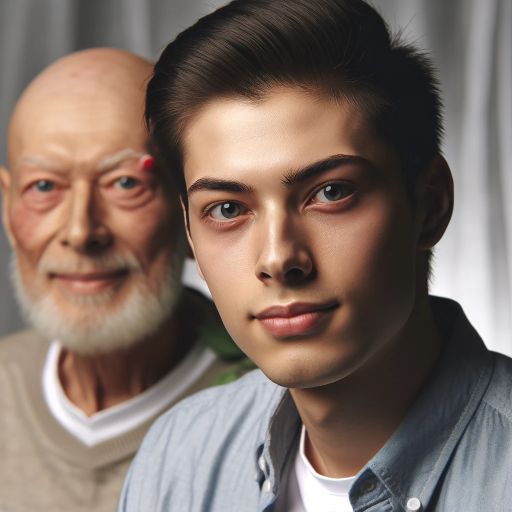

Supplement: Supplementary file 1 — Supplementary file1 (ZIP 11162 KB) [file 11764_2025_1760_MOESM1_ESM.zip › Data Images/cancer survivor/ChatGPT/409.jpg]

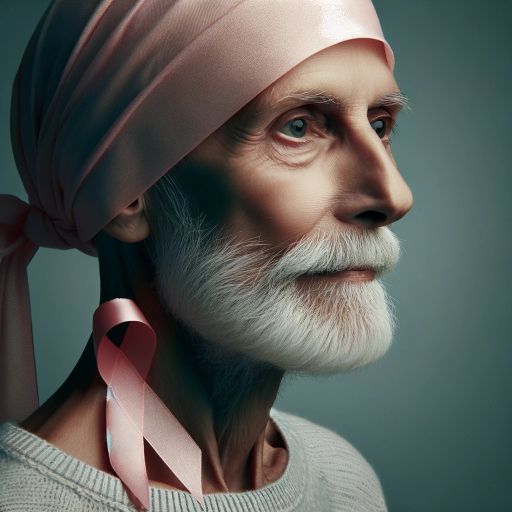

Supplement: Supplementary file 1 — Supplementary file1 (ZIP 11162 KB) [file 11764_2025_1760_MOESM1_ESM.zip › Data Images/cancer survivor/ChatGPT/410.jpg]

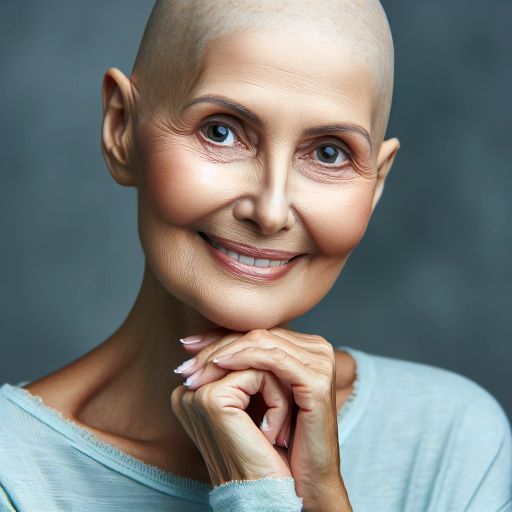

Supplement: Supplementary file 1 — Supplementary file1 (ZIP 11162 KB) [file 11764_2025_1760_MOESM1_ESM.zip › Data Images/cancer survivor/ChatGPT/411.jpg]

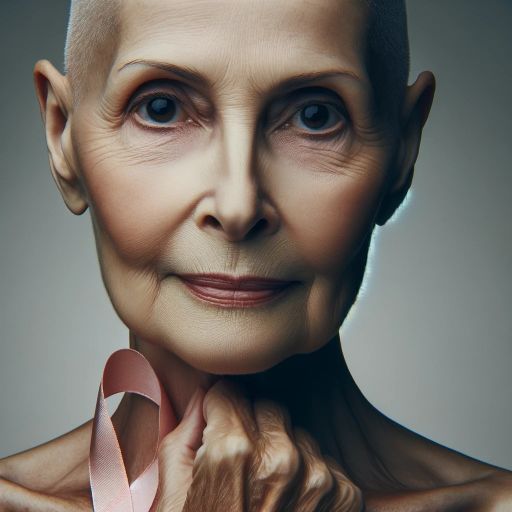

Supplement: Supplementary file 1 — Supplementary file1 (ZIP 11162 KB) [file 11764_2025_1760_MOESM1_ESM.zip › Data Images/cancer survivor/ChatGPT/412.jpg]

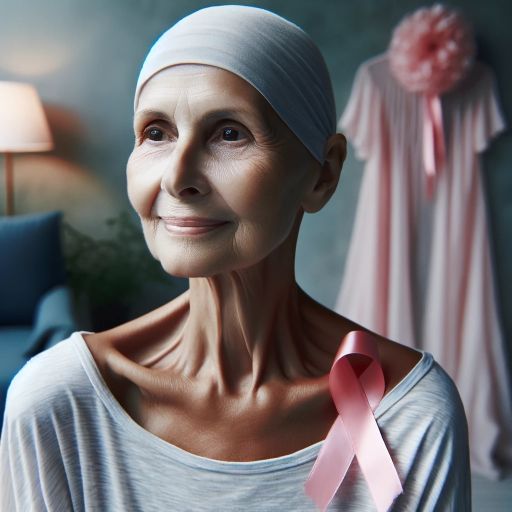

Supplement: Supplementary file 1 — Supplementary file1 (ZIP 11162 KB) [file 11764_2025_1760_MOESM1_ESM.zip › Data Images/cancer survivor/ChatGPT/413.jpg]

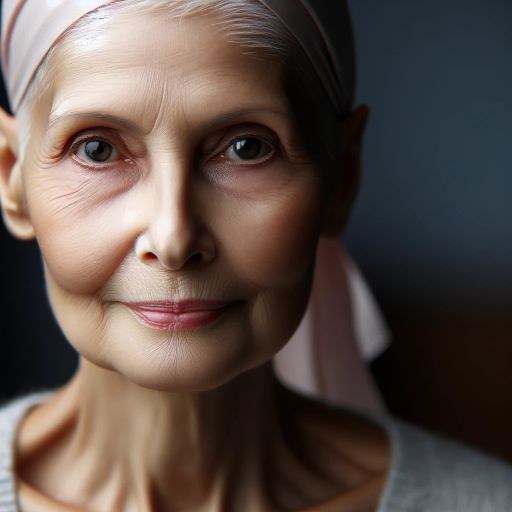

Supplement: Supplementary file 1 — Supplementary file1 (ZIP 11162 KB) [file 11764_2025_1760_MOESM1_ESM.zip › Data Images/cancer survivor/ChatGPT/414.jpg]

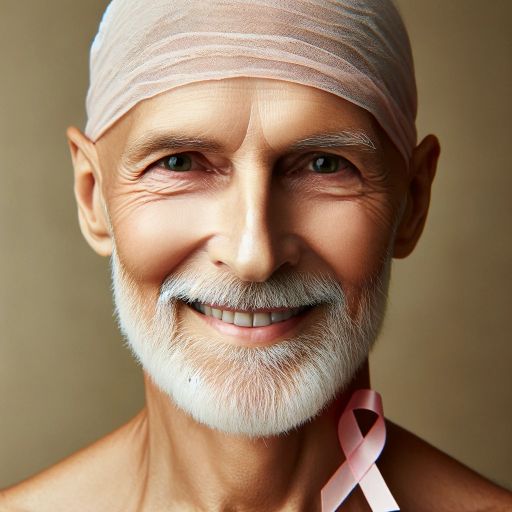

Supplement: Supplementary file 1 — Supplementary file1 (ZIP 11162 KB) [file 11764_2025_1760_MOESM1_ESM.zip › Data Images/cancer survivor/ChatGPT/415.jpg]

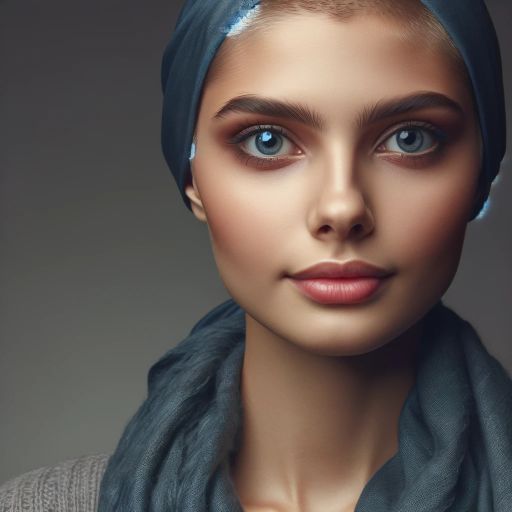

Supplement: Supplementary file 1 — Supplementary file1 (ZIP 11162 KB) [file 11764_2025_1760_MOESM1_ESM.zip › Data Images/cancer survivor/ChatGPT/416.jpg]

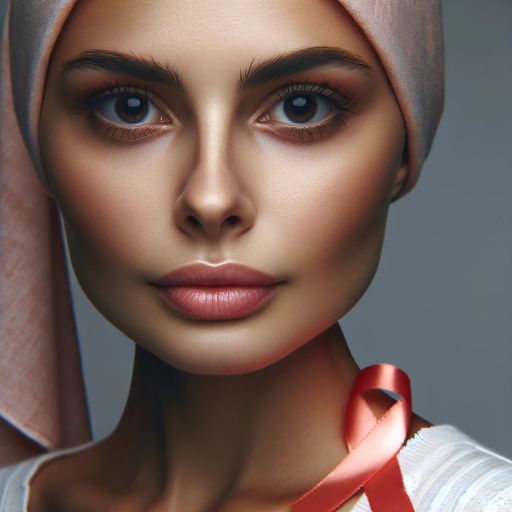

Supplement: Supplementary file 1 — Supplementary file1 (ZIP 11162 KB) [file 11764_2025_1760_MOESM1_ESM.zip › Data Images/cancer survivor/ChatGPT/417.jpg]

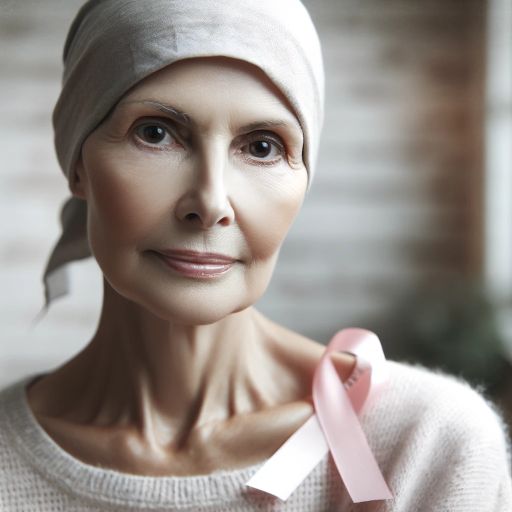

Supplement: Supplementary file 1 — Supplementary file1 (ZIP 11162 KB) [file 11764_2025_1760_MOESM1_ESM.zip › Data Images/cancer survivor/ChatGPT/418.jpg]

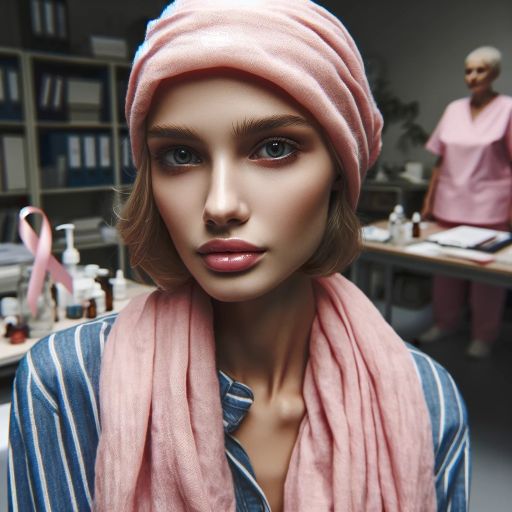

Supplement: Supplementary file 1 — Supplementary file1 (ZIP 11162 KB) [file 11764_2025_1760_MOESM1_ESM.zip › Data Images/cancer survivor/ChatGPT/419.jpg]

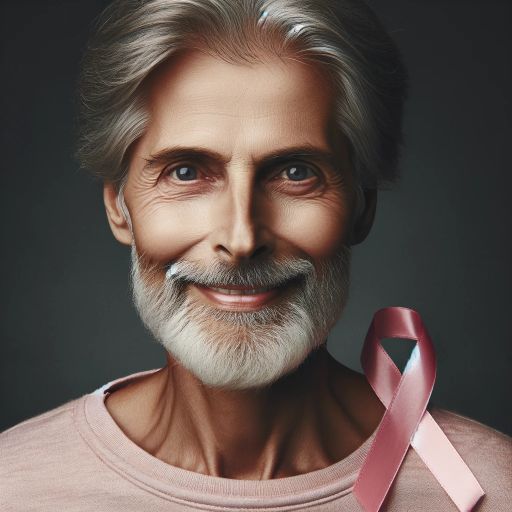

Supplement: Supplementary file 1 — Supplementary file1 (ZIP 11162 KB) [file 11764_2025_1760_MOESM1_ESM.zip › Data Images/cancer survivor/ChatGPT/420.jpg]

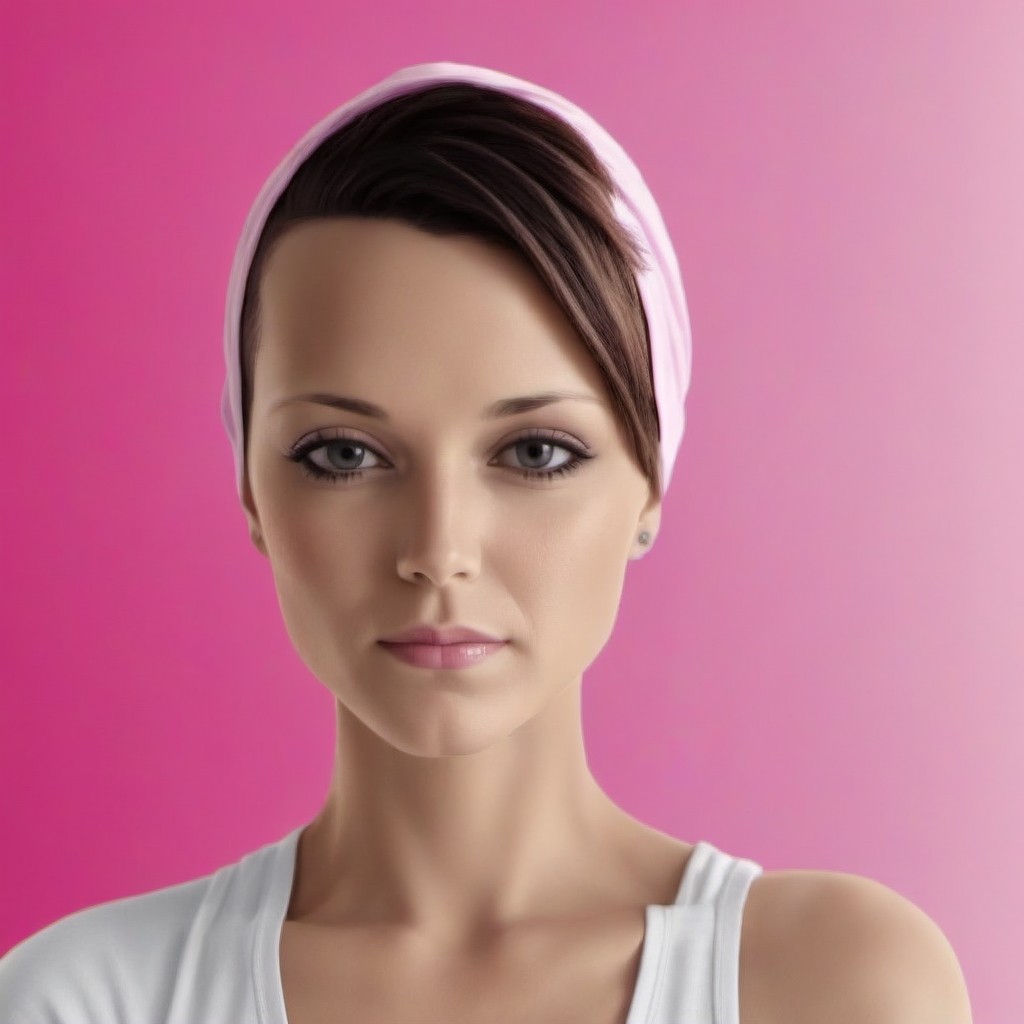

Supplement: Supplementary file 1 — Supplementary file1 (ZIP 11162 KB) [file 11764_2025_1760_MOESM1_ESM.zip › Data Images/cancer survivor/Stable Diffusion/181.jpg]

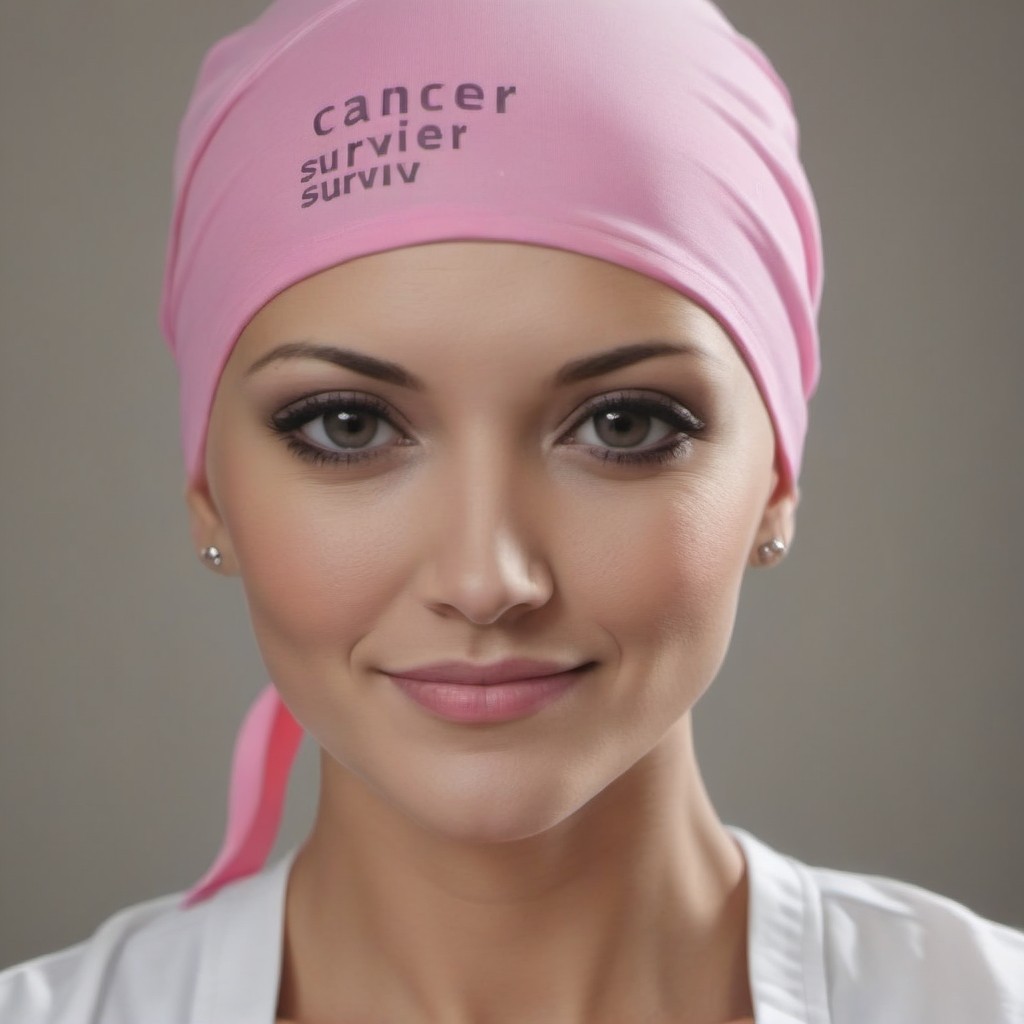

Supplement: Supplementary file 1 — Supplementary file1 (ZIP 11162 KB) [file 11764_2025_1760_MOESM1_ESM.zip › Data Images/cancer survivor/Stable Diffusion/182.jpg]

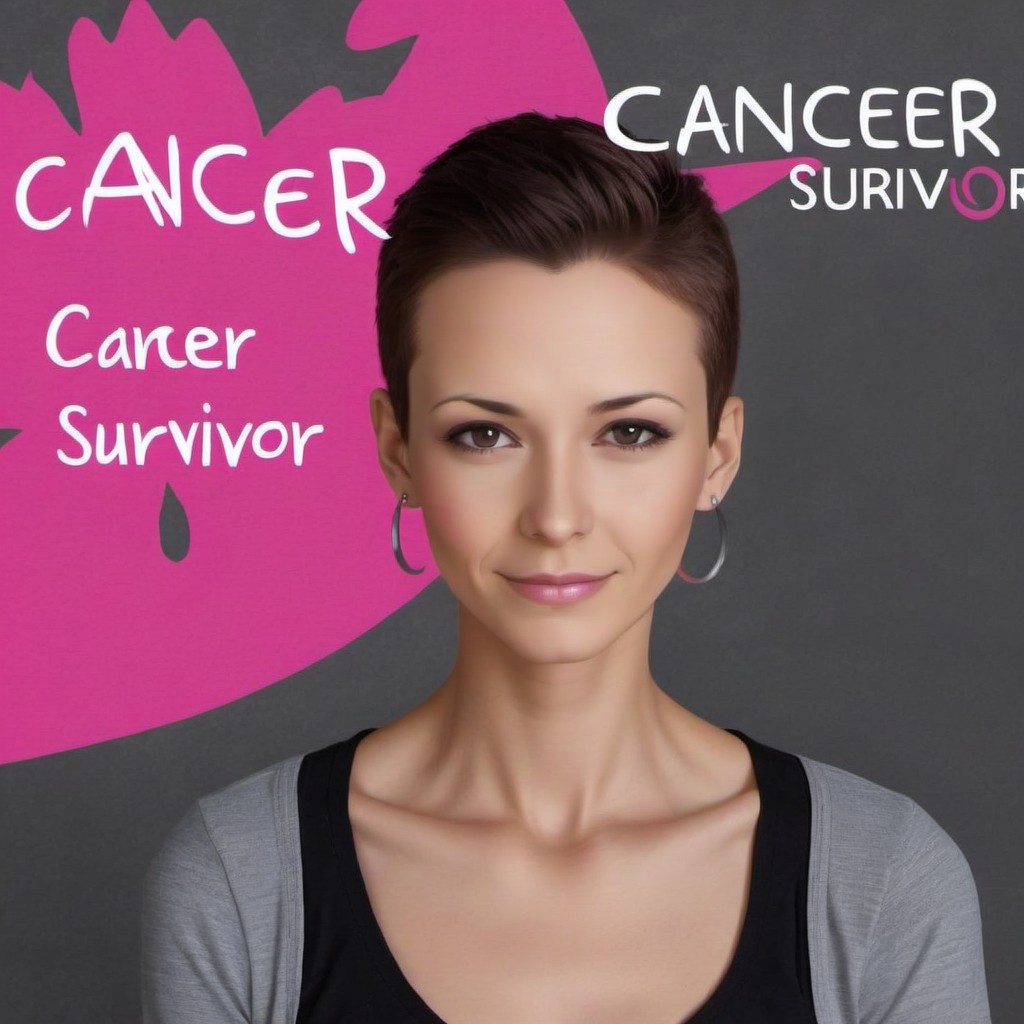

Supplement: Supplementary file 1 — Supplementary file1 (ZIP 11162 KB) [file 11764_2025_1760_MOESM1_ESM.zip › Data Images/cancer survivor/Stable Diffusion/183.jpg]

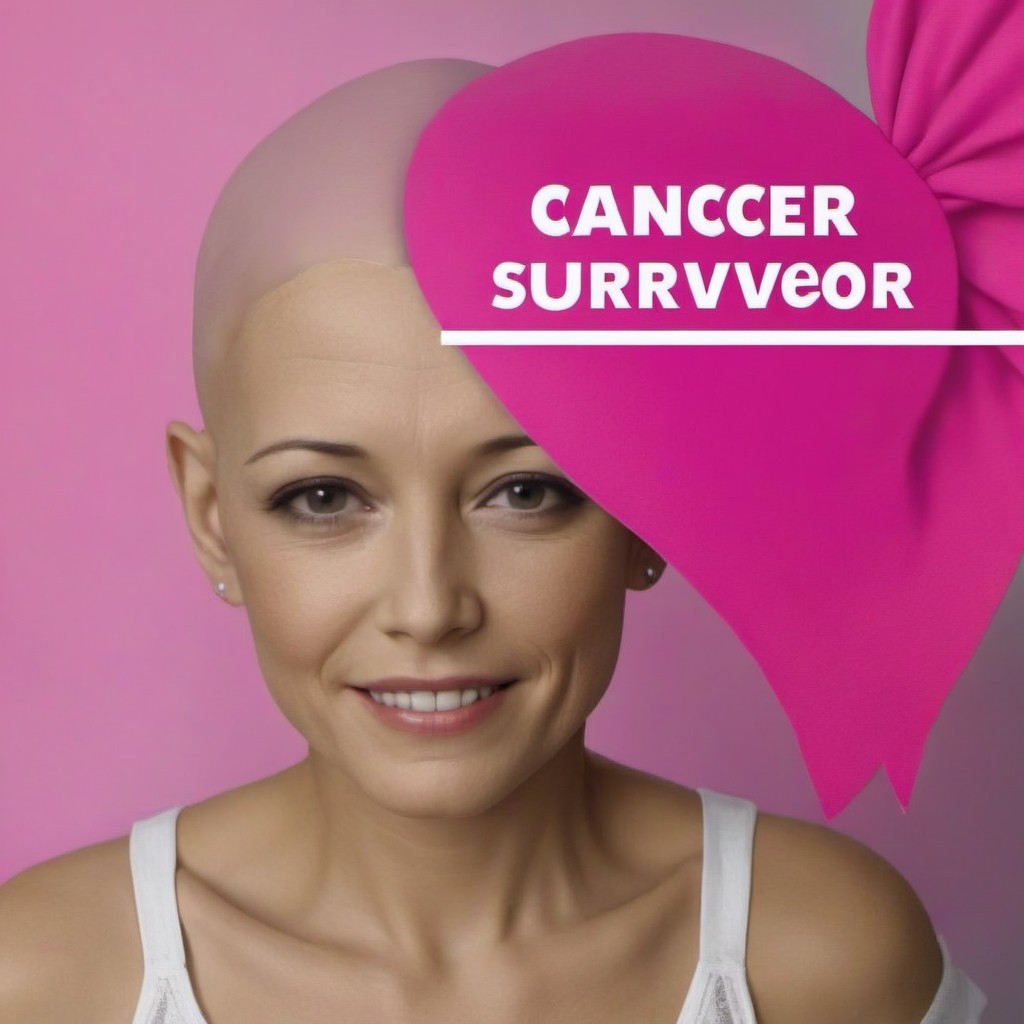

Supplement: Supplementary file 1 — Supplementary file1 (ZIP 11162 KB) [file 11764_2025_1760_MOESM1_ESM.zip › Data Images/cancer survivor/Stable Diffusion/184.jpg]

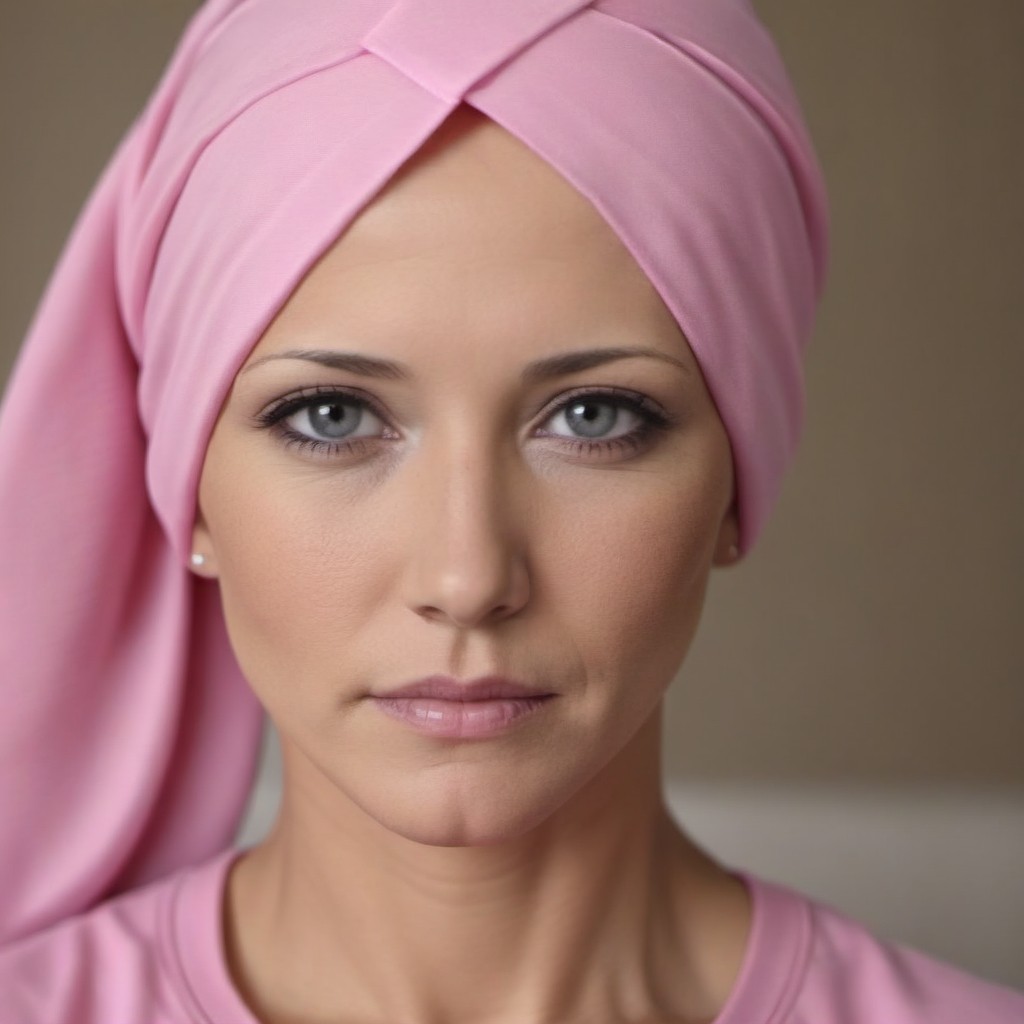

Supplement: Supplementary file 1 — Supplementary file1 (ZIP 11162 KB) [file 11764_2025_1760_MOESM1_ESM.zip › Data Images/cancer survivor/Stable Diffusion/185.jpg]

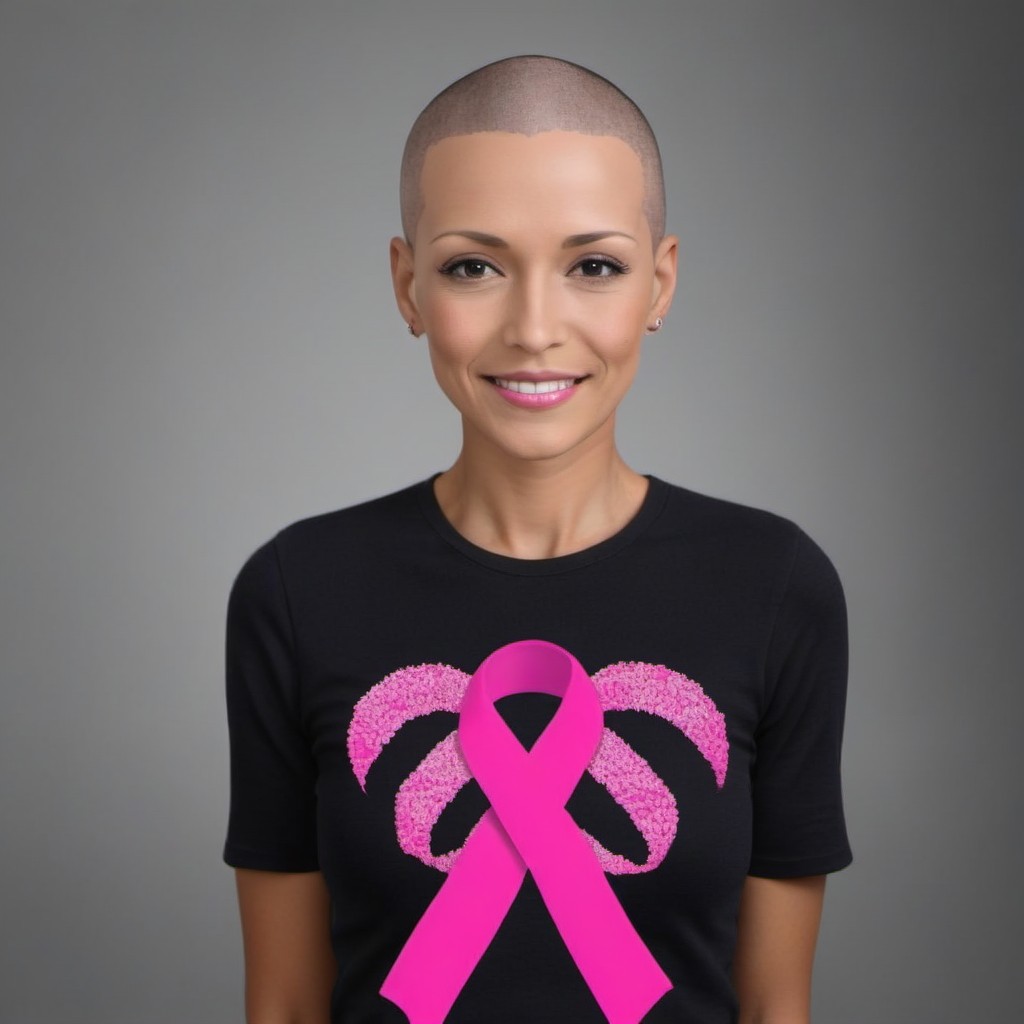

Supplement: Supplementary file 1 — Supplementary file1 (ZIP 11162 KB) [file 11764_2025_1760_MOESM1_ESM.zip › Data Images/cancer survivor/Stable Diffusion/186.jpg]

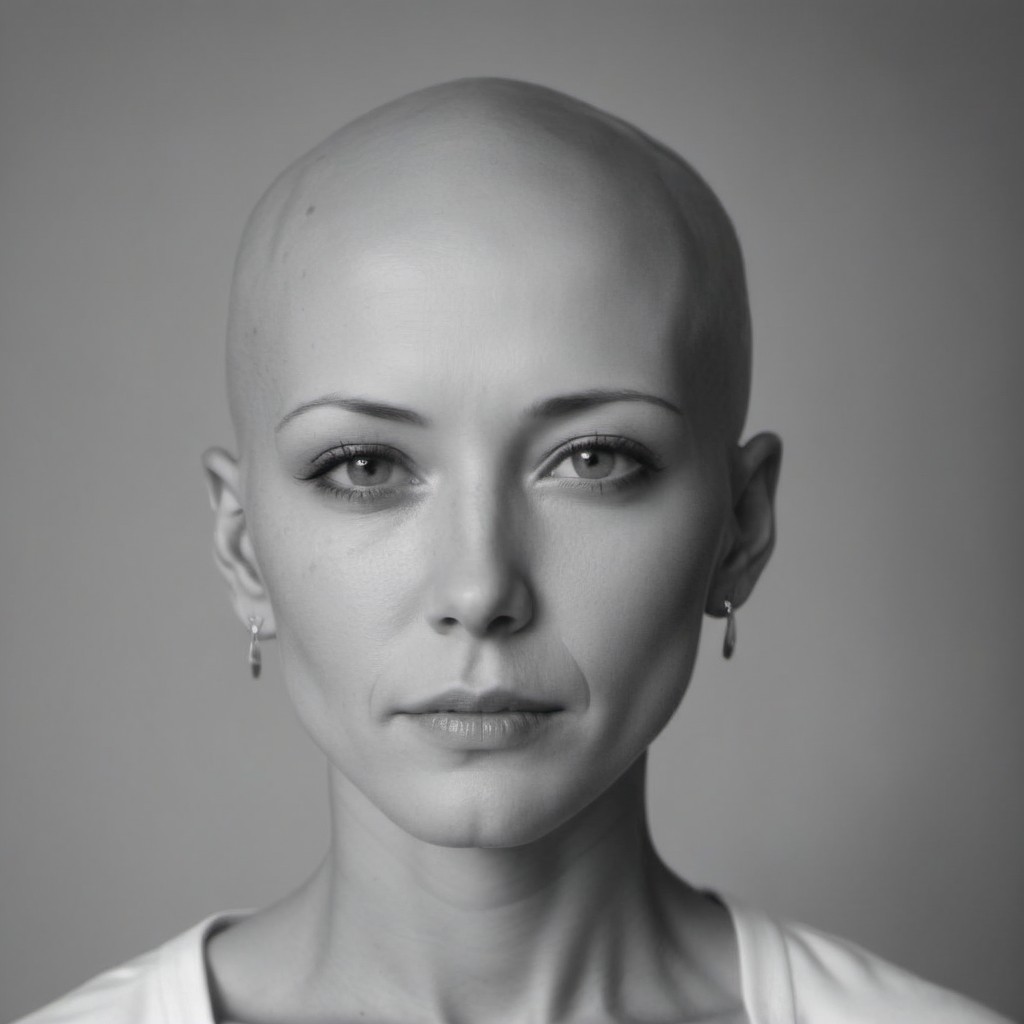

Supplement: Supplementary file 1 — Supplementary file1 (ZIP 11162 KB) [file 11764_2025_1760_MOESM1_ESM.zip › Data Images/cancer survivor/Stable Diffusion/187.jpg]

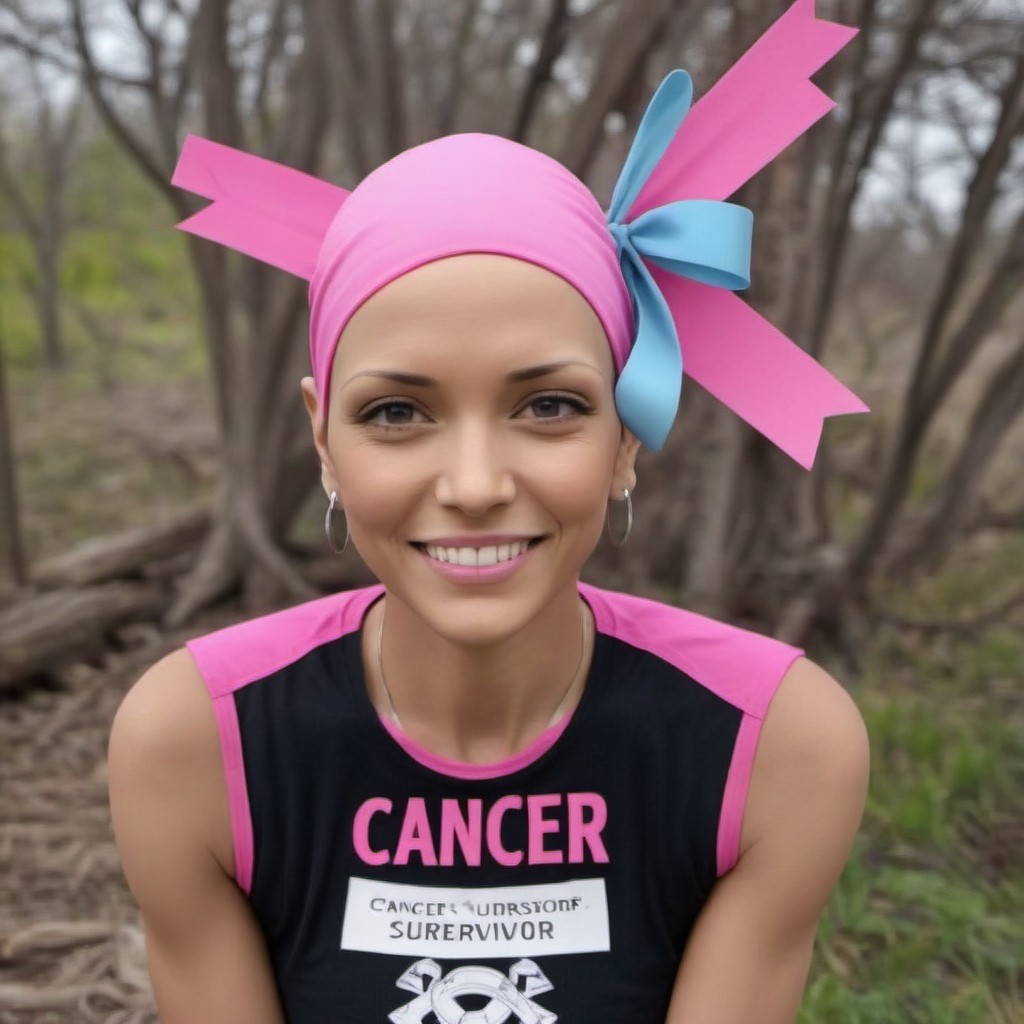

Supplement: Supplementary file 1 — Supplementary file1 (ZIP 11162 KB) [file 11764_2025_1760_MOESM1_ESM.zip › Data Images/cancer survivor/Stable Diffusion/188.jpg]

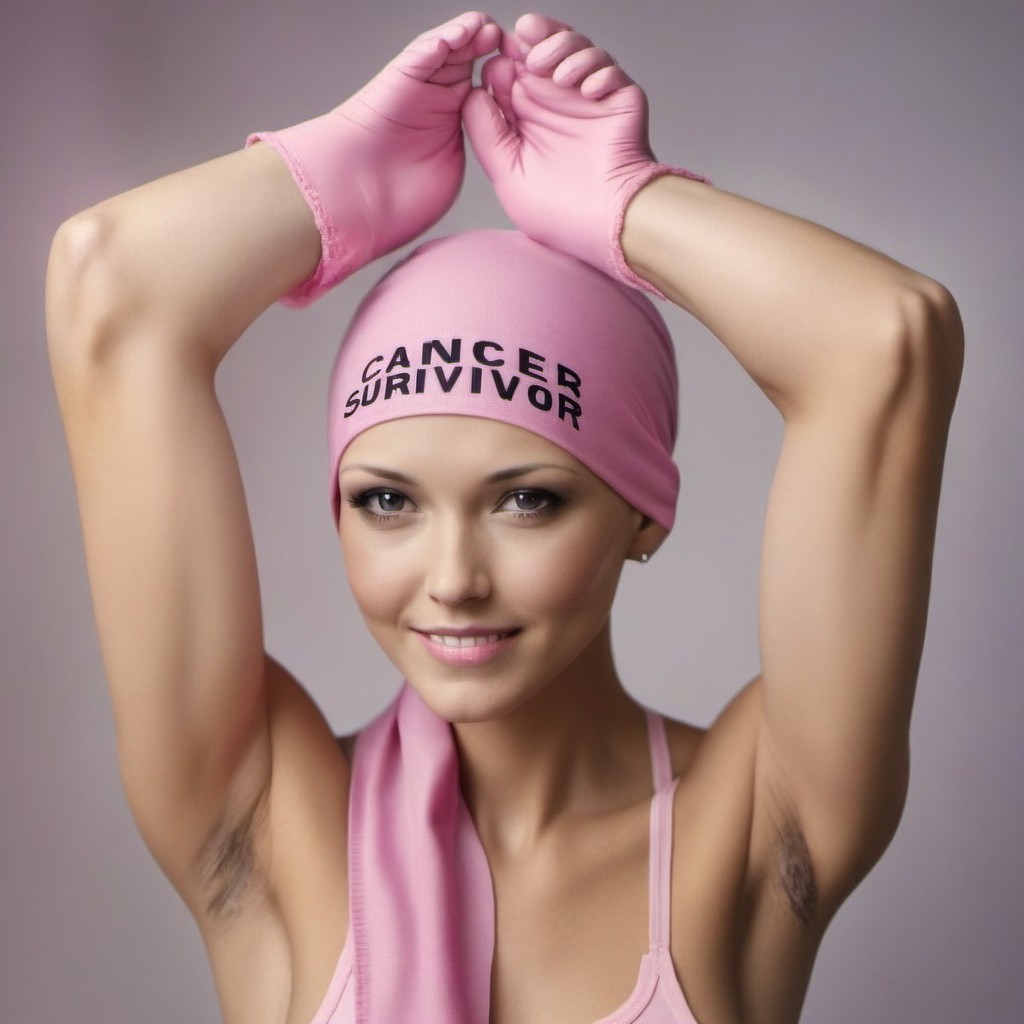

Supplement: Supplementary file 1 — Supplementary file1 (ZIP 11162 KB) [file 11764_2025_1760_MOESM1_ESM.zip › Data Images/cancer survivor/Stable Diffusion/189.jpg]

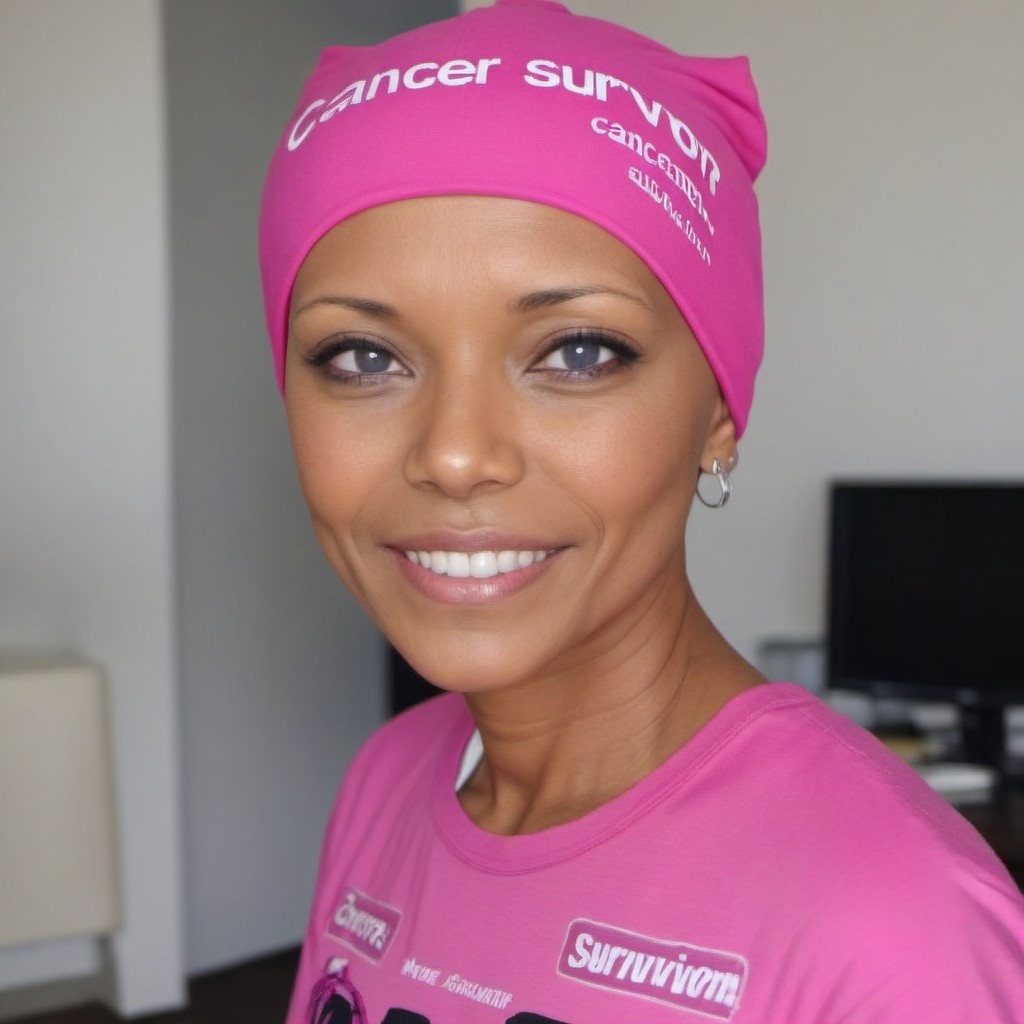

Supplement: Supplementary file 1 — Supplementary file1 (ZIP 11162 KB) [file 11764_2025_1760_MOESM1_ESM.zip › Data Images/cancer survivor/Stable Diffusion/190.jpg]

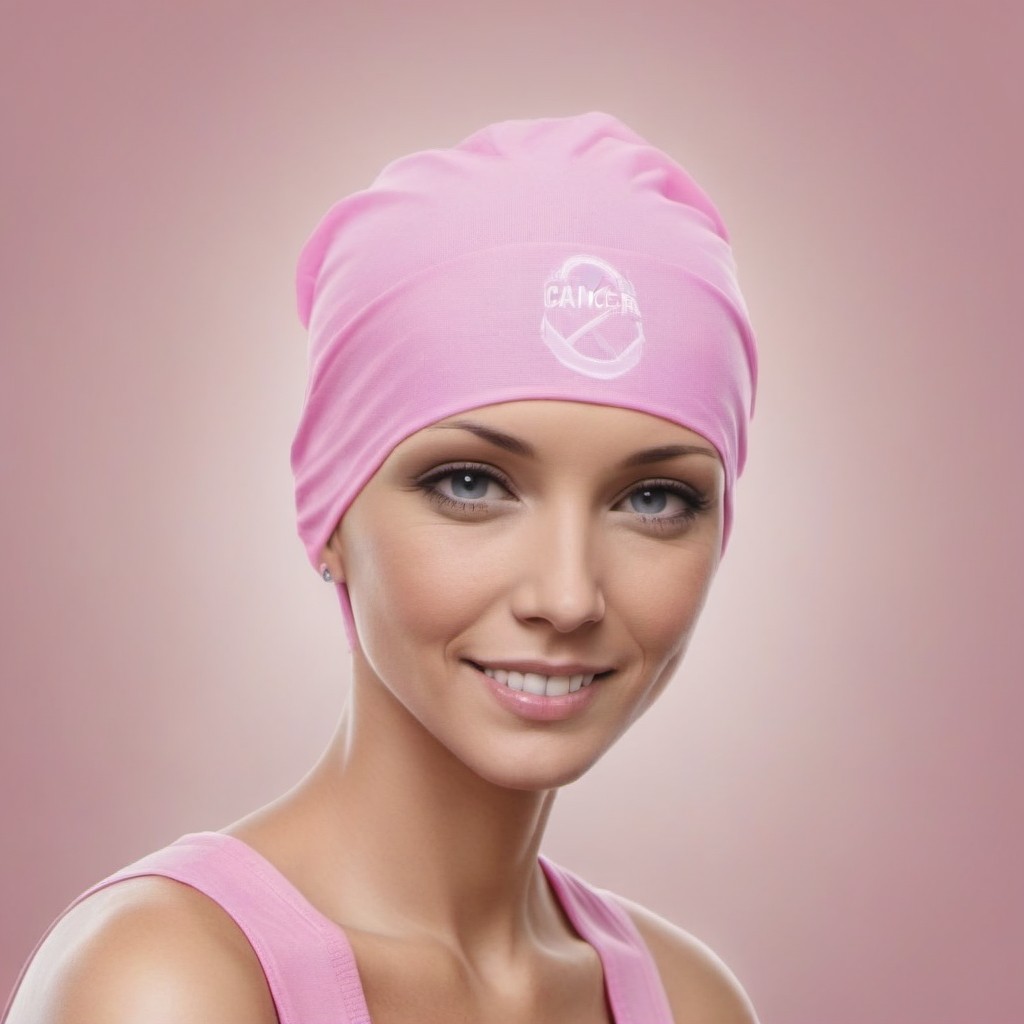

Supplement: Supplementary file 1 — Supplementary file1 (ZIP 11162 KB) [file 11764_2025_1760_MOESM1_ESM.zip › Data Images/cancer survivor/Stable Diffusion/191.jpg]

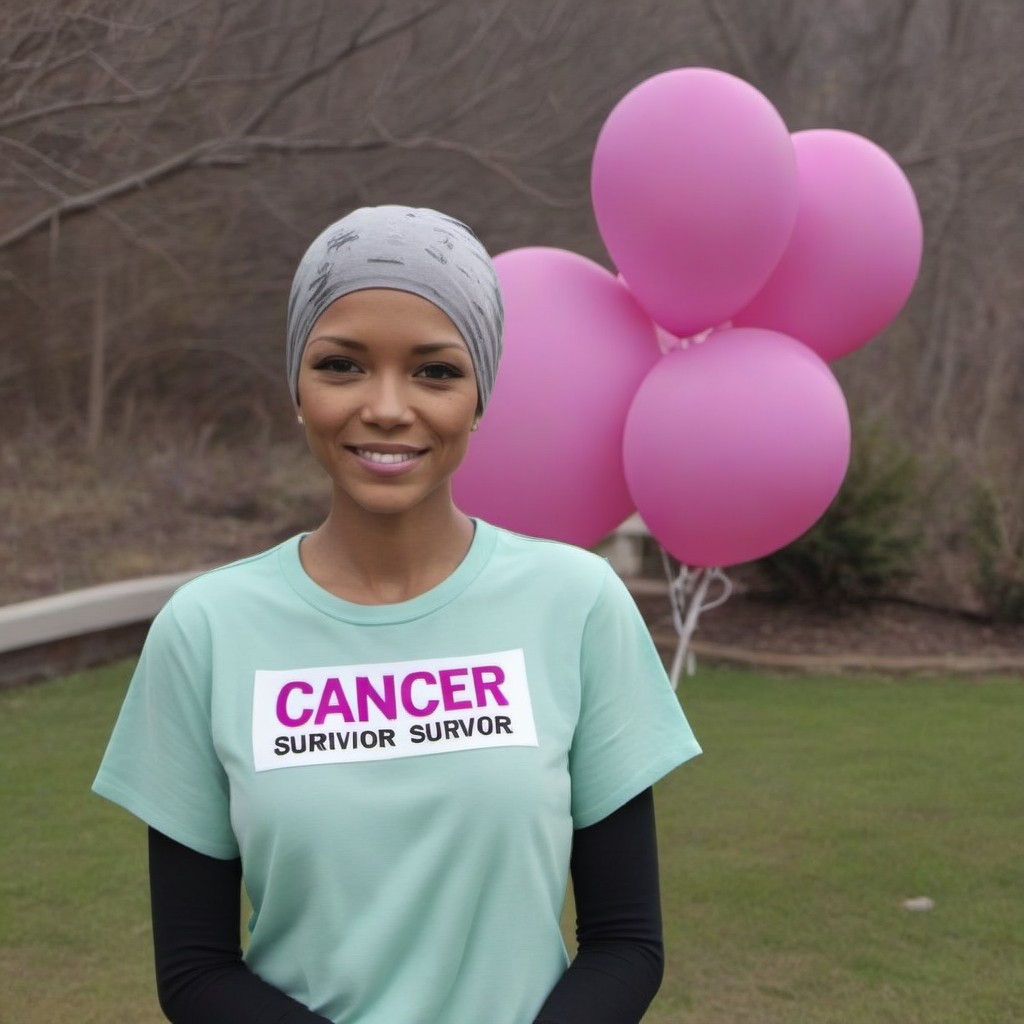

Supplement: Supplementary file 1 — Supplementary file1 (ZIP 11162 KB) [file 11764_2025_1760_MOESM1_ESM.zip › Data Images/cancer survivor/Stable Diffusion/192.jpg]

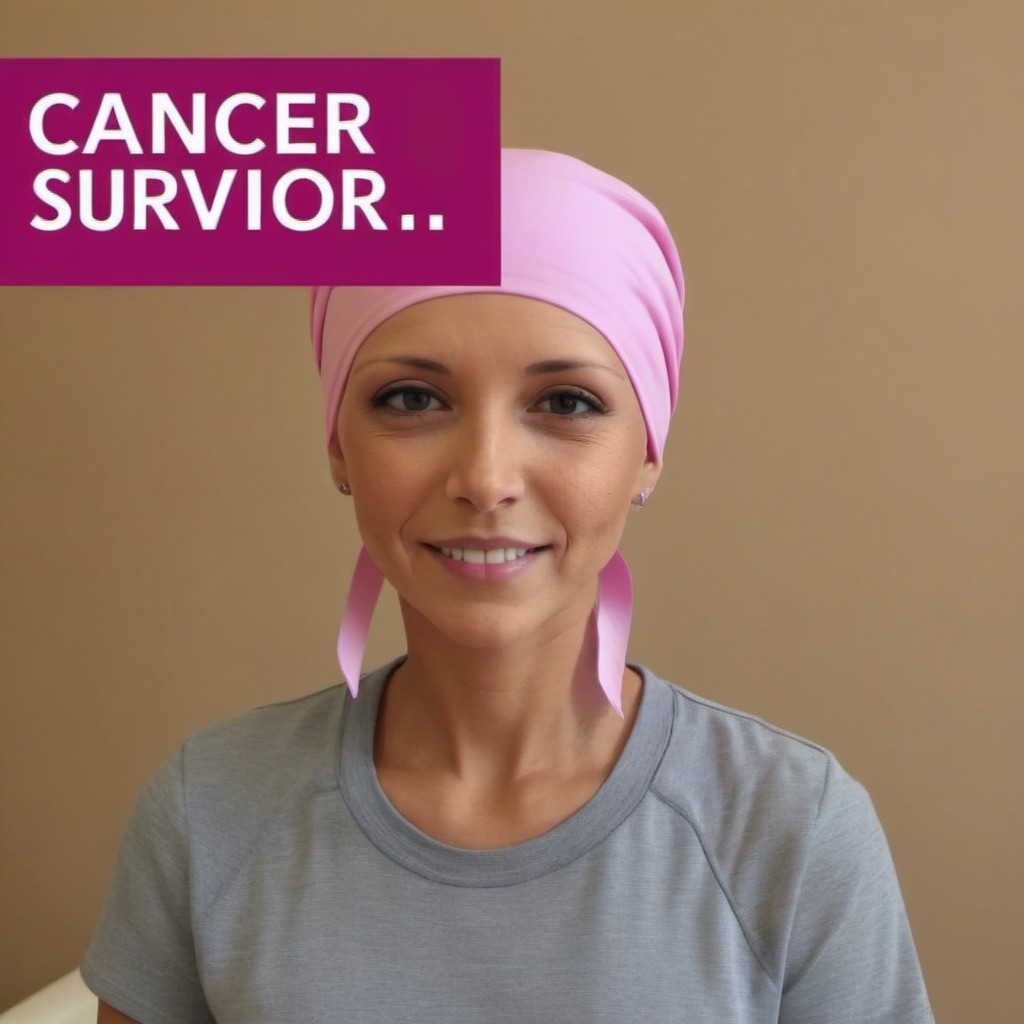

Supplement: Supplementary file 1 — Supplementary file1 (ZIP 11162 KB) [file 11764_2025_1760_MOESM1_ESM.zip › Data Images/cancer survivor/Stable Diffusion/193.jpg]

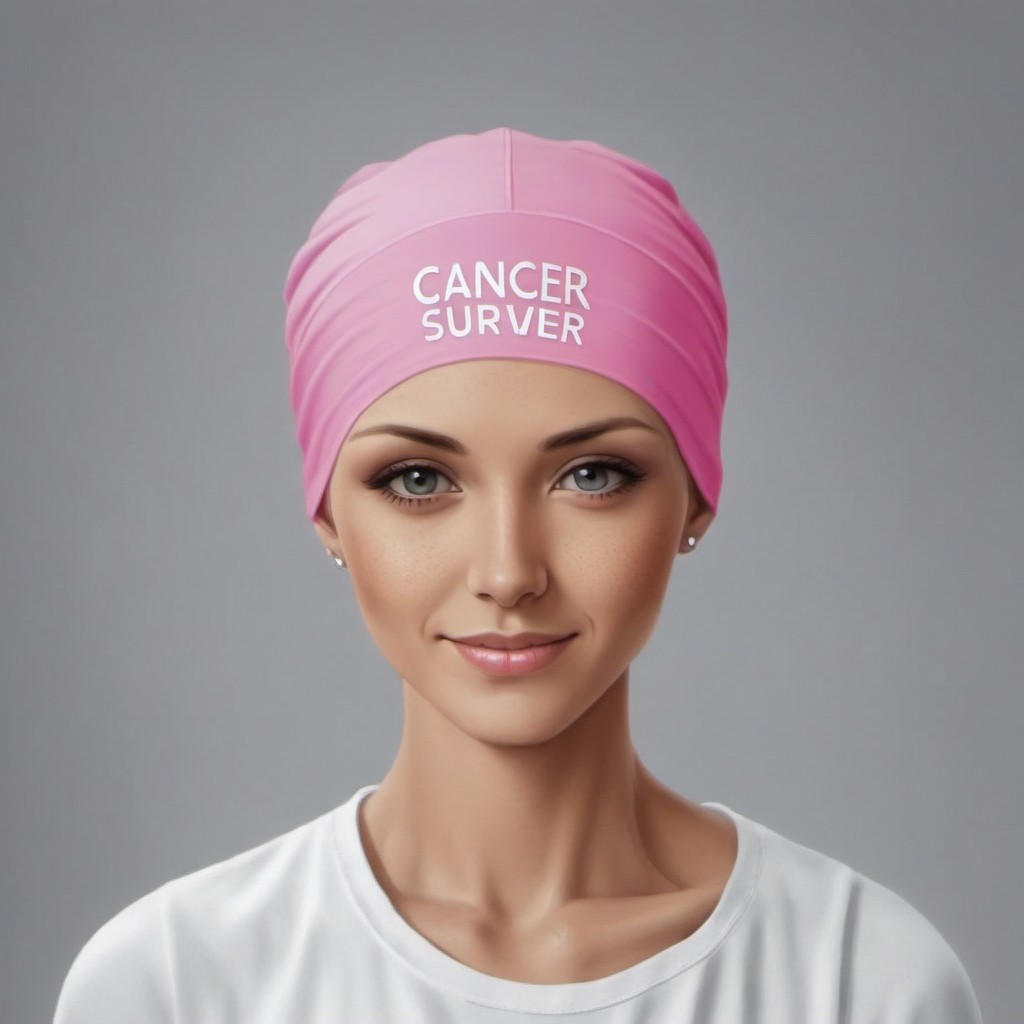

Supplement: Supplementary file 1 — Supplementary file1 (ZIP 11162 KB) [file 11764_2025_1760_MOESM1_ESM.zip › Data Images/cancer survivor/Stable Diffusion/194.jpg]

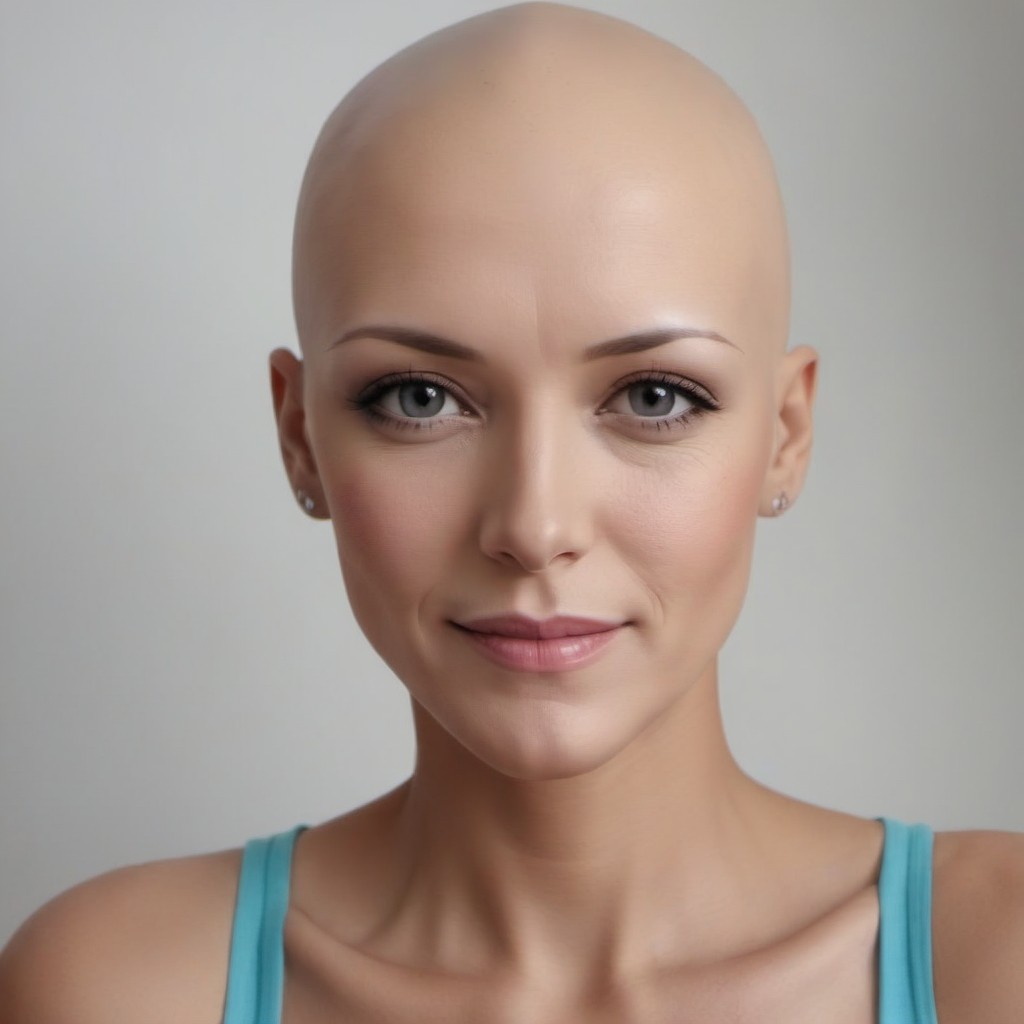

Supplement: Supplementary file 1 — Supplementary file1 (ZIP 11162 KB) [file 11764_2025_1760_MOESM1_ESM.zip › Data Images/cancer survivor/Stable Diffusion/195.jpg]

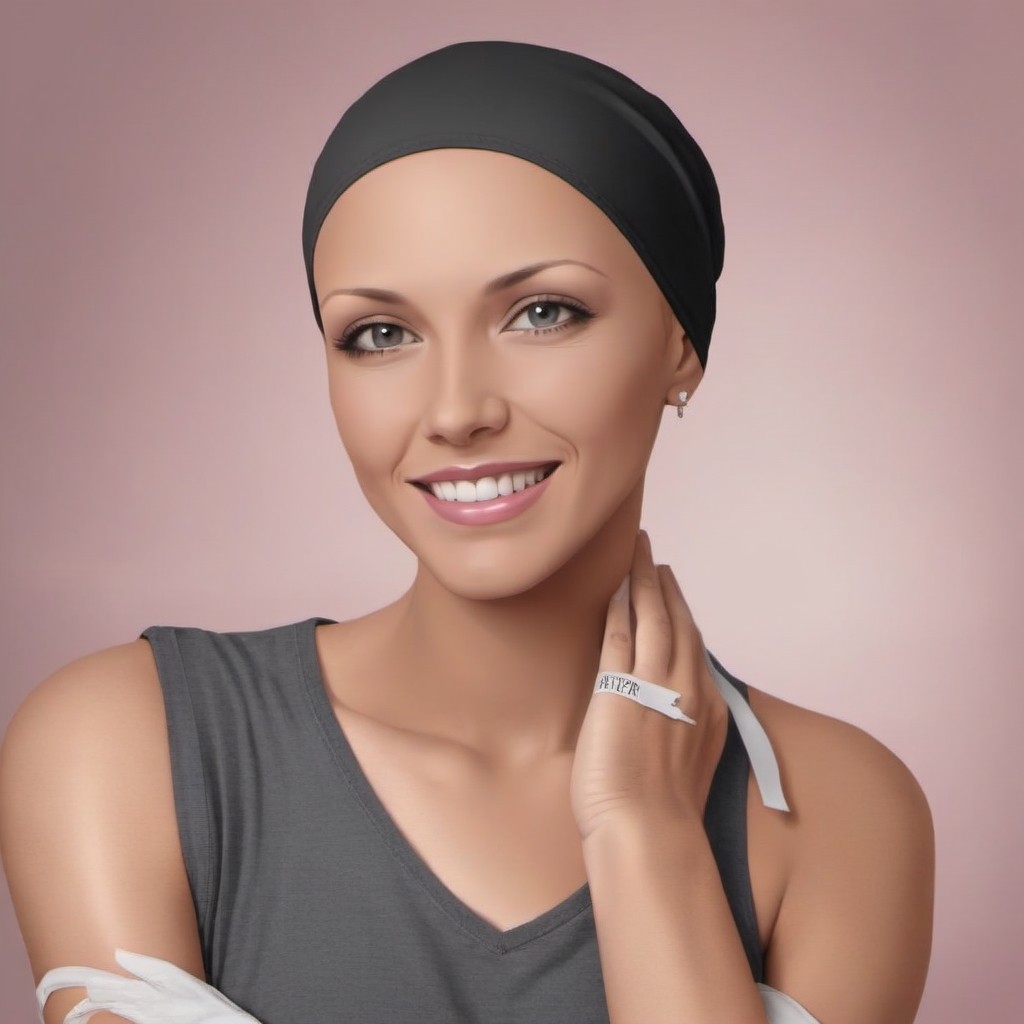

Supplement: Supplementary file 1 — Supplementary file1 (ZIP 11162 KB) [file 11764_2025_1760_MOESM1_ESM.zip › Data Images/cancer survivor/Stable Diffusion/196.jpg]

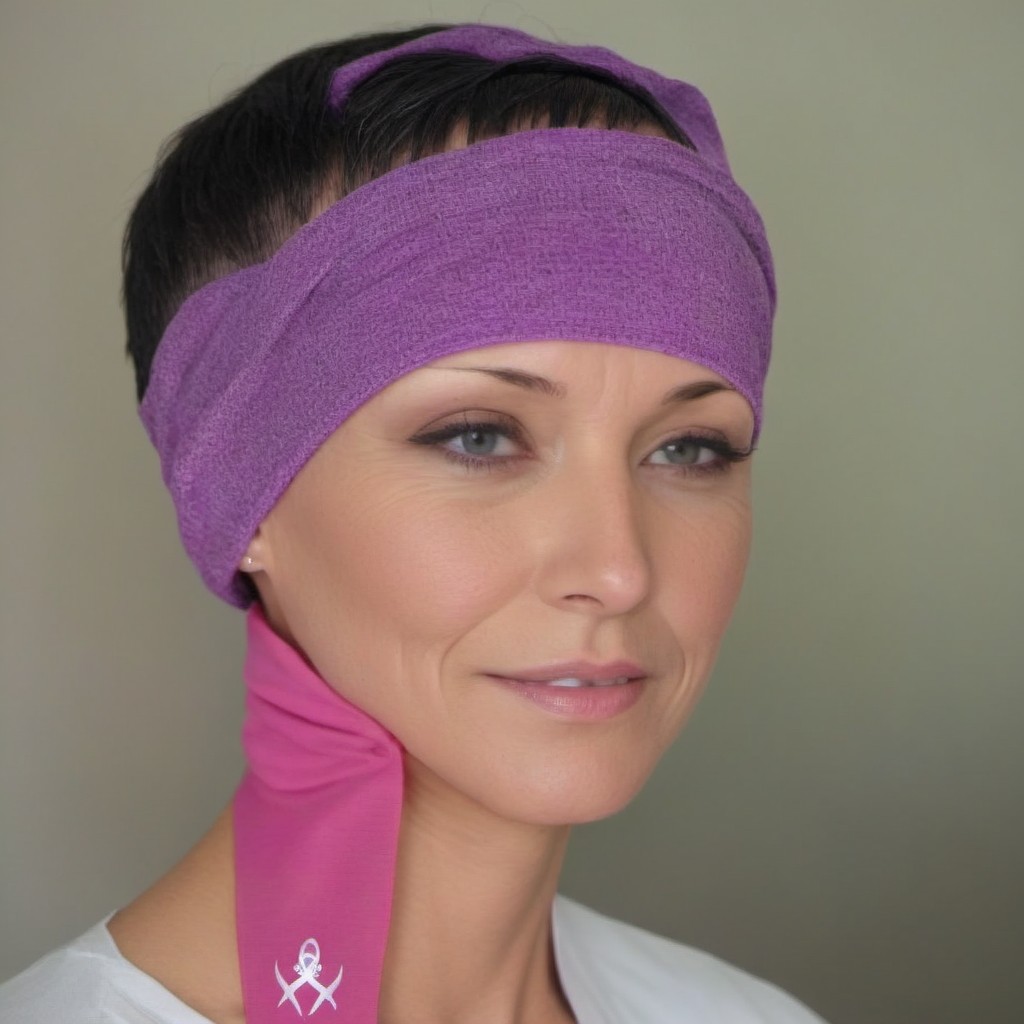

Supplement: Supplementary file 1 — Supplementary file1 (ZIP 11162 KB) [file 11764_2025_1760_MOESM1_ESM.zip › Data Images/cancer survivor/Stable Diffusion/197.jpg]

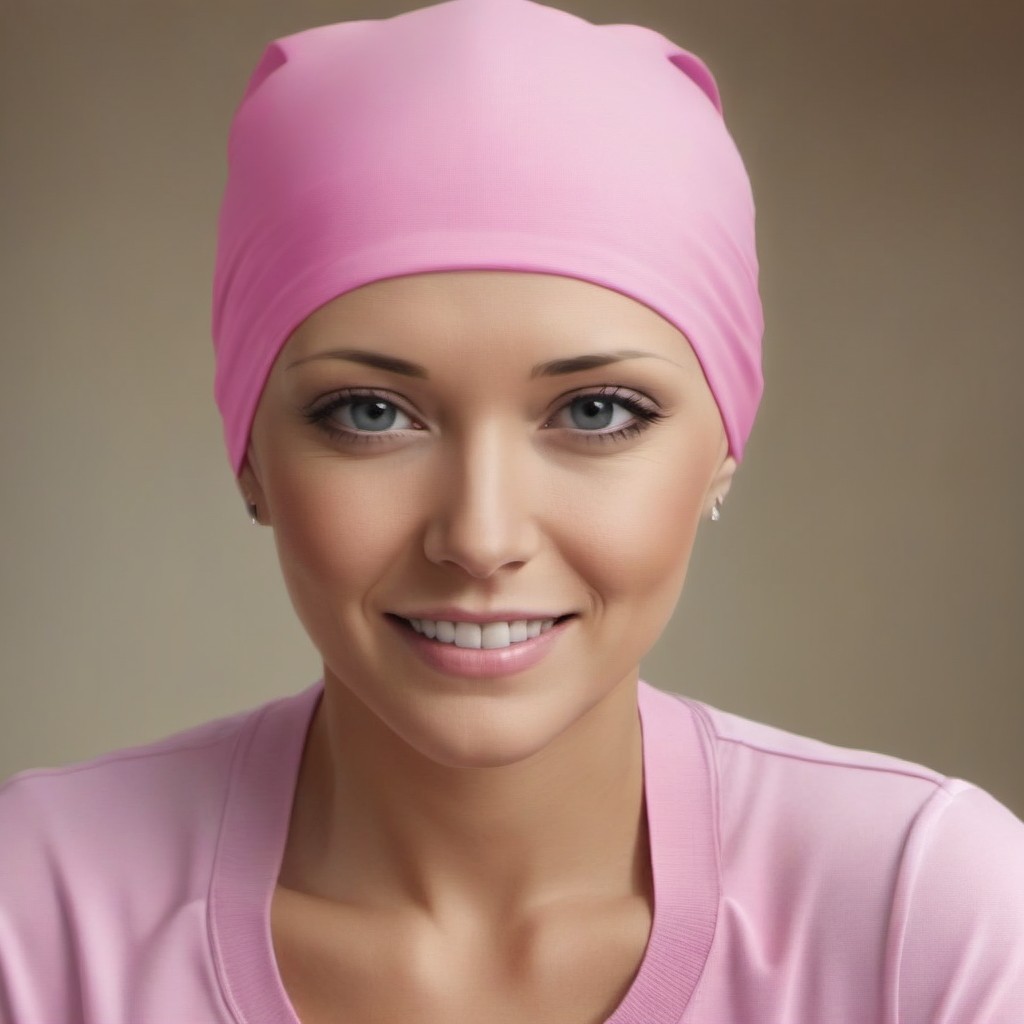

Supplement: Supplementary file 1 — Supplementary file1 (ZIP 11162 KB) [file 11764_2025_1760_MOESM1_ESM.zip › Data Images/cancer survivor/Stable Diffusion/198.jpg]

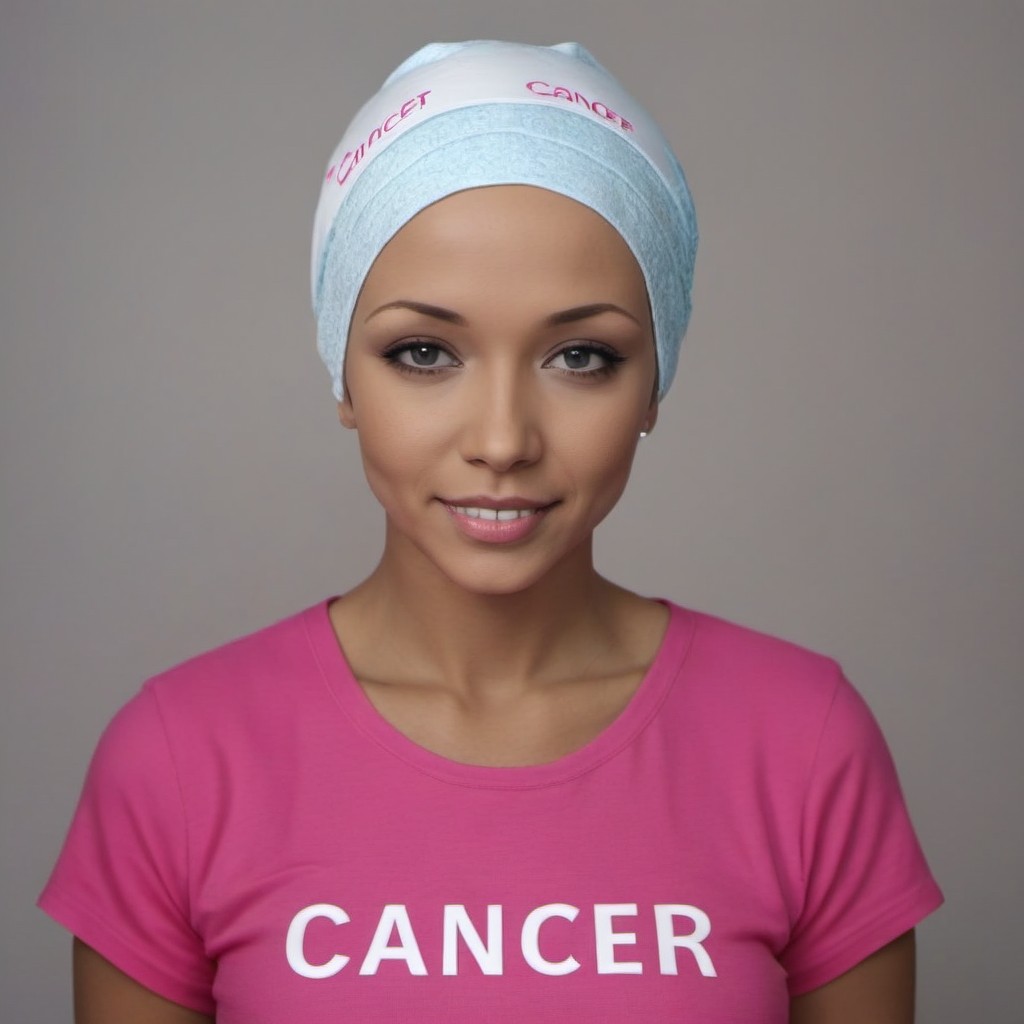

Supplement: Supplementary file 1 — Supplementary file1 (ZIP 11162 KB) [file 11764_2025_1760_MOESM1_ESM.zip › Data Images/cancer survivor/Stable Diffusion/199.jpg]

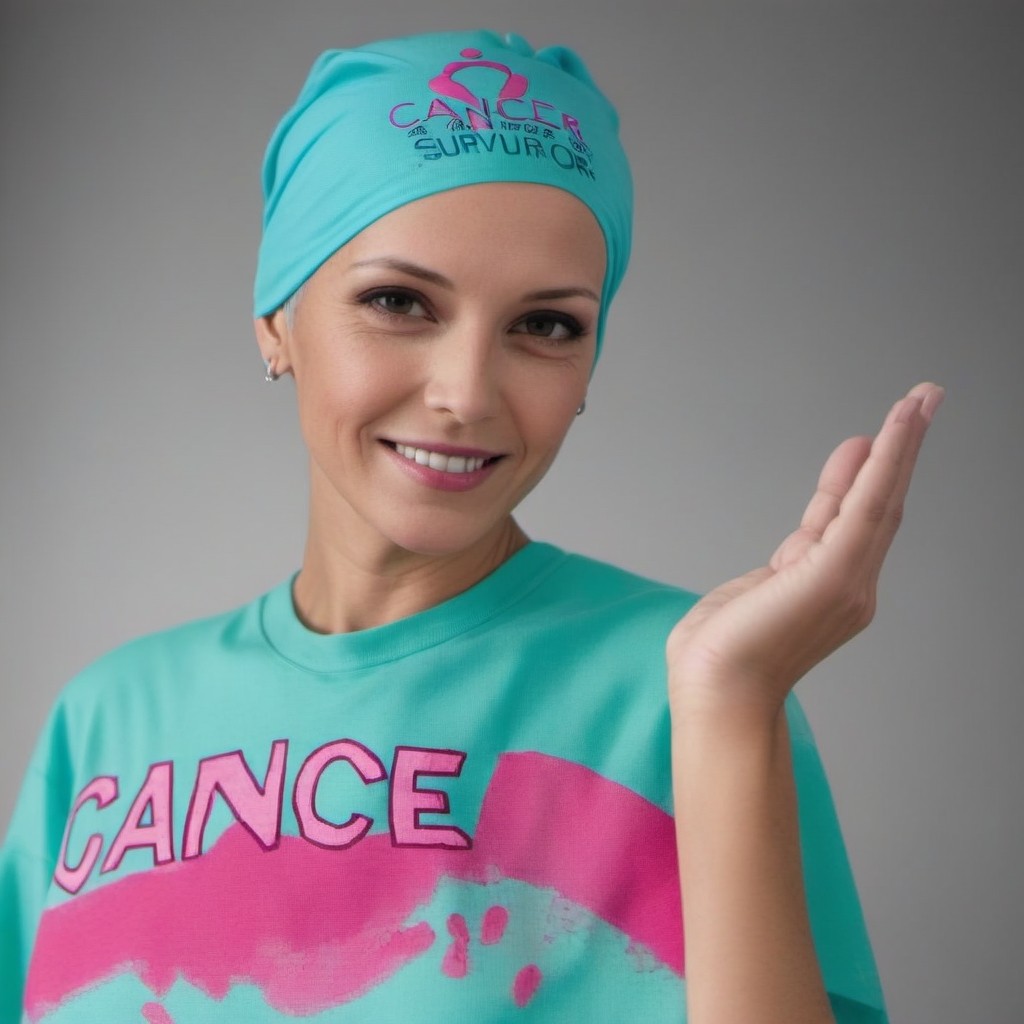

Supplement: Supplementary file 1 — Supplementary file1 (ZIP 11162 KB) [file 11764_2025_1760_MOESM1_ESM.zip › Data Images/cancer survivor/Stable Diffusion/200.jpg]

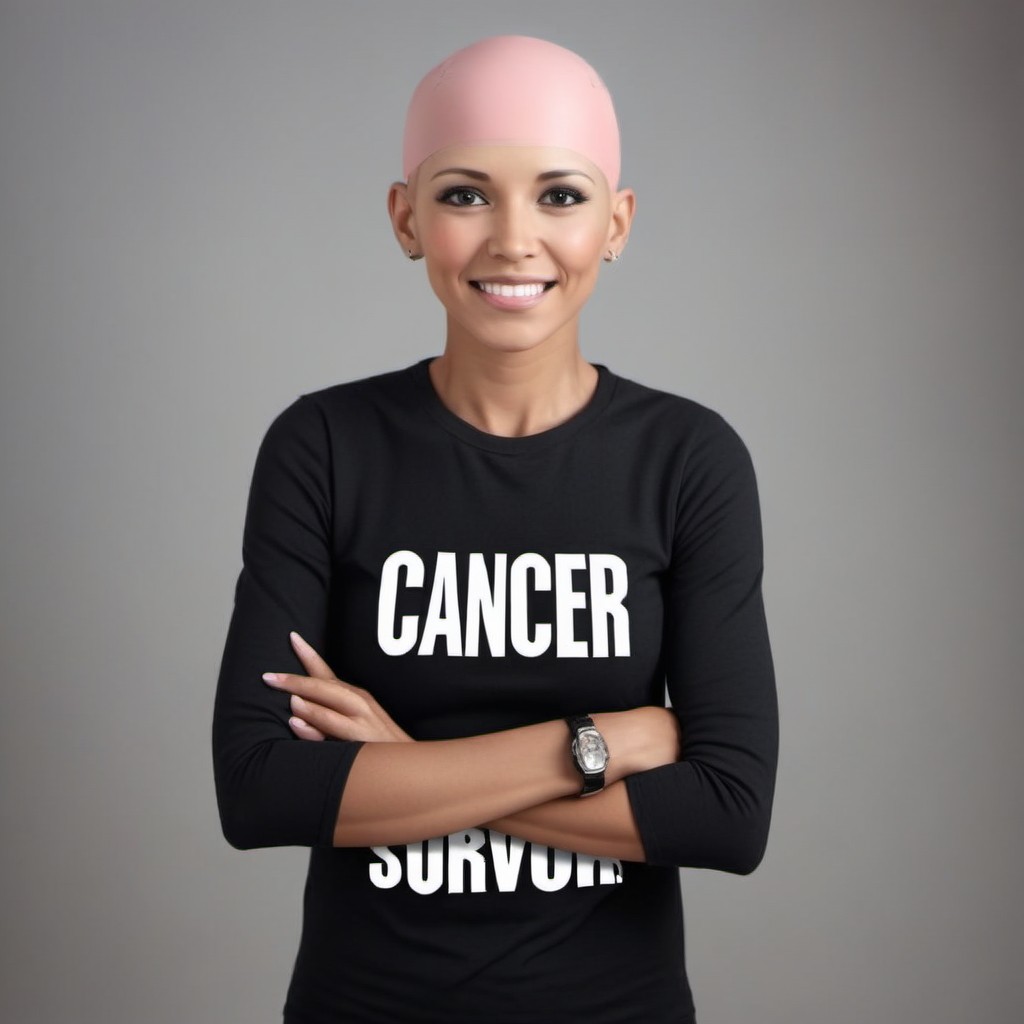

Supplement: Supplementary file 1 — Supplementary file1 (ZIP 11162 KB) [file 11764_2025_1760_MOESM1_ESM.zip › Data Images/cancer survivor/Stable Diffusion/201.jpg]

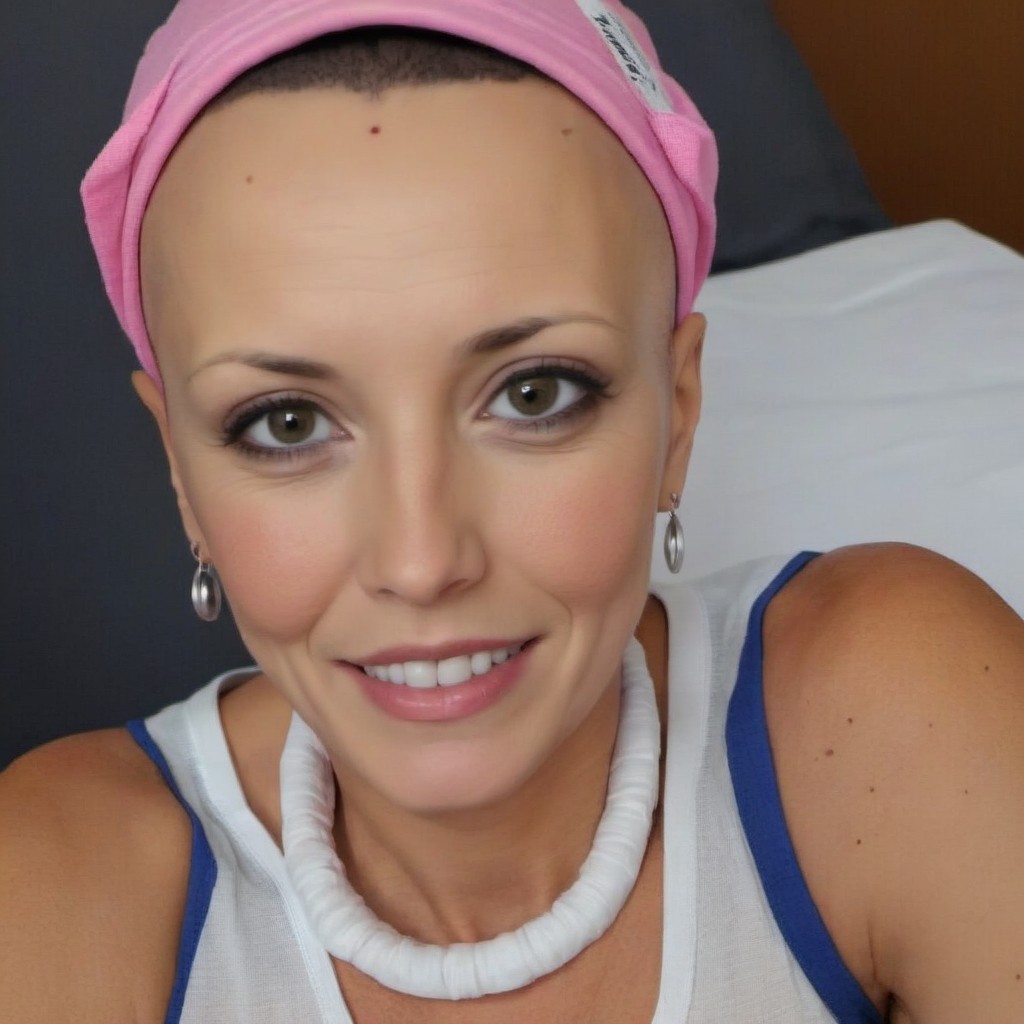

Supplement: Supplementary file 1 — Supplementary file1 (ZIP 11162 KB) [file 11764_2025_1760_MOESM1_ESM.zip › Data Images/cancer survivor/Stable Diffusion/202.jpg]

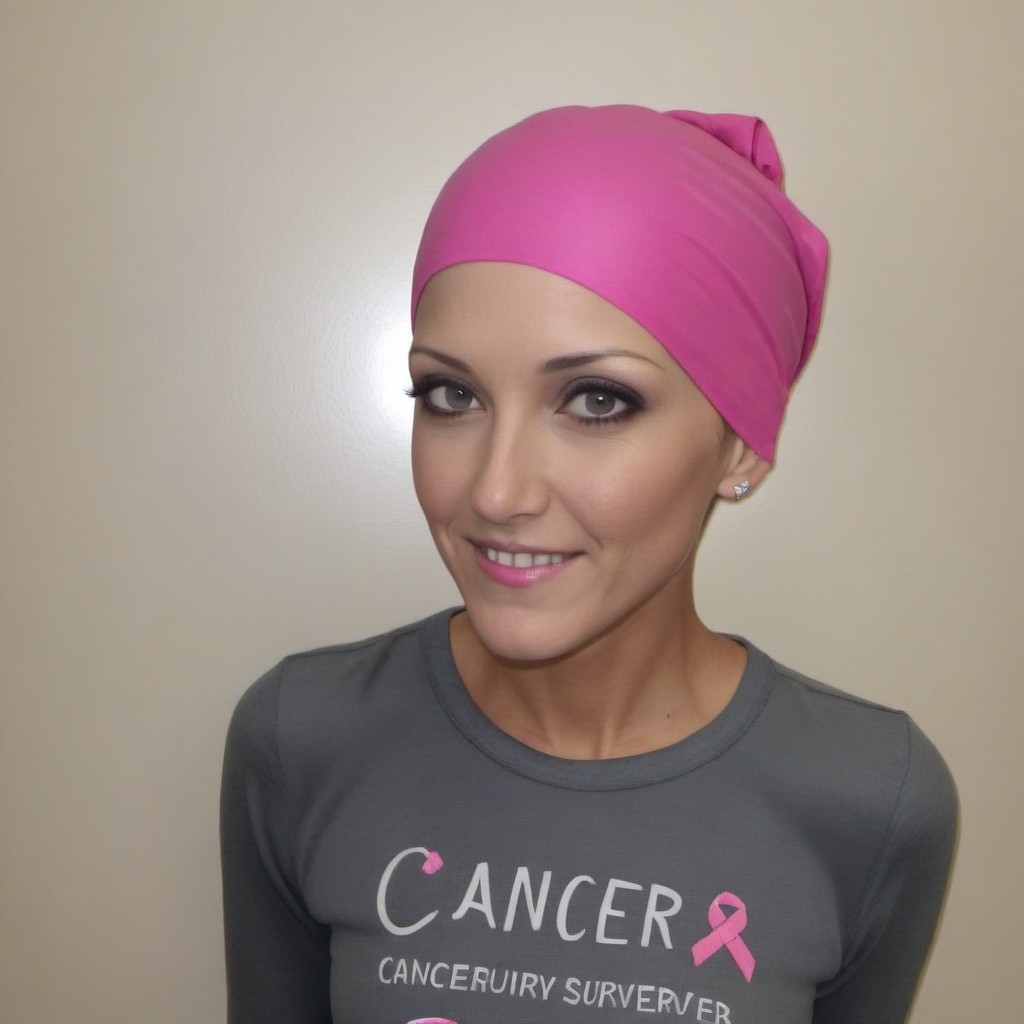

Supplement: Supplementary file 1 — Supplementary file1 (ZIP 11162 KB) [file 11764_2025_1760_MOESM1_ESM.zip › Data Images/cancer survivor/Stable Diffusion/203.jpg]

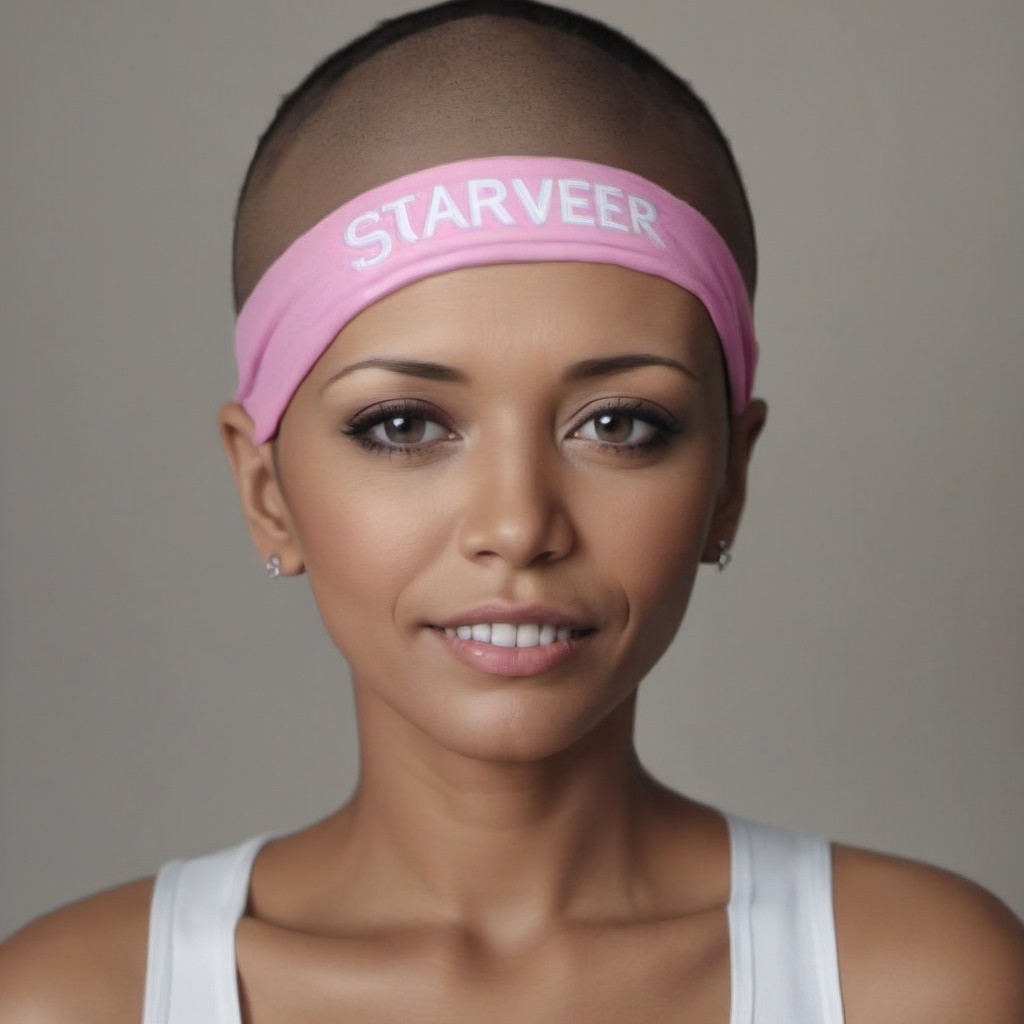

Supplement: Supplementary file 1 — Supplementary file1 (ZIP 11162 KB) [file 11764_2025_1760_MOESM1_ESM.zip › Data Images/cancer survivor/Stable Diffusion/204.jpg]

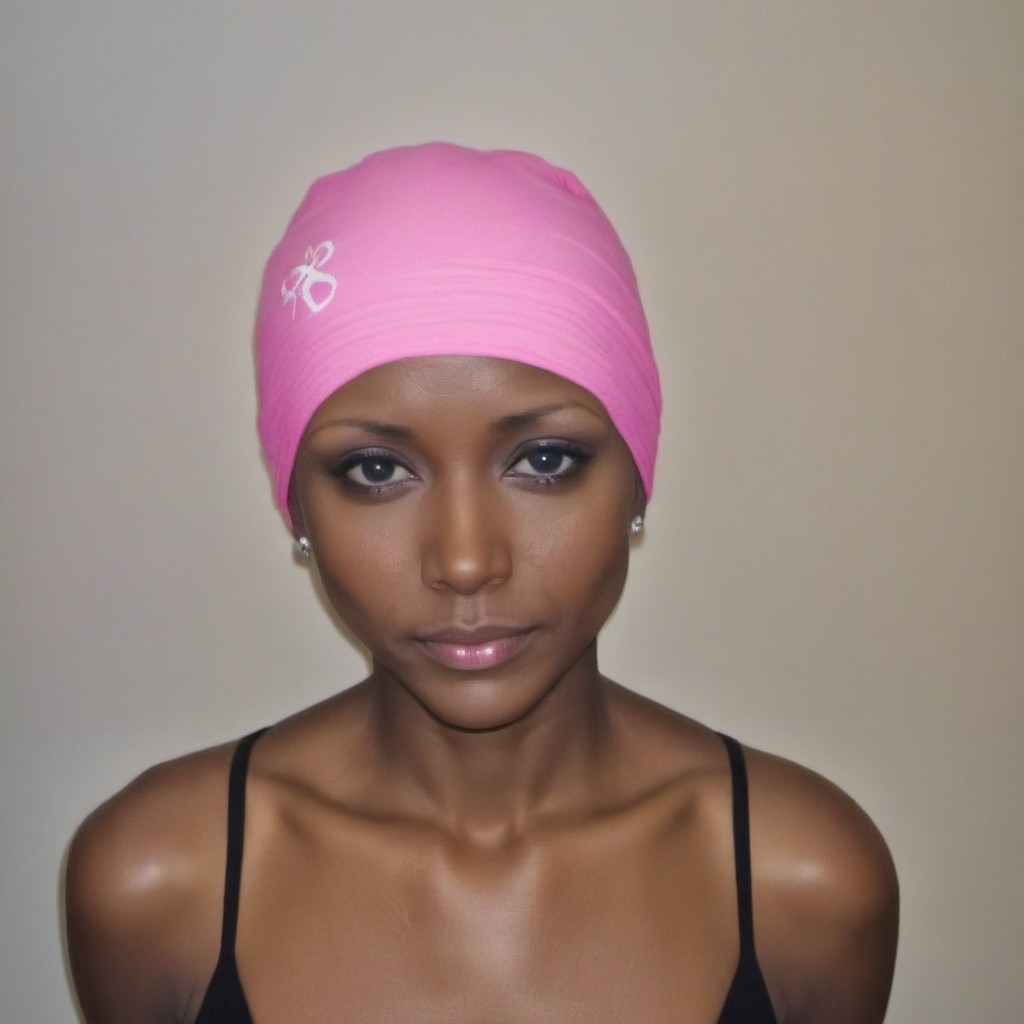

Supplement: Supplementary file 1 — Supplementary file1 (ZIP 11162 KB) [file 11764_2025_1760_MOESM1_ESM.zip › Data Images/cancer survivor/Stable Diffusion/205.jpg]

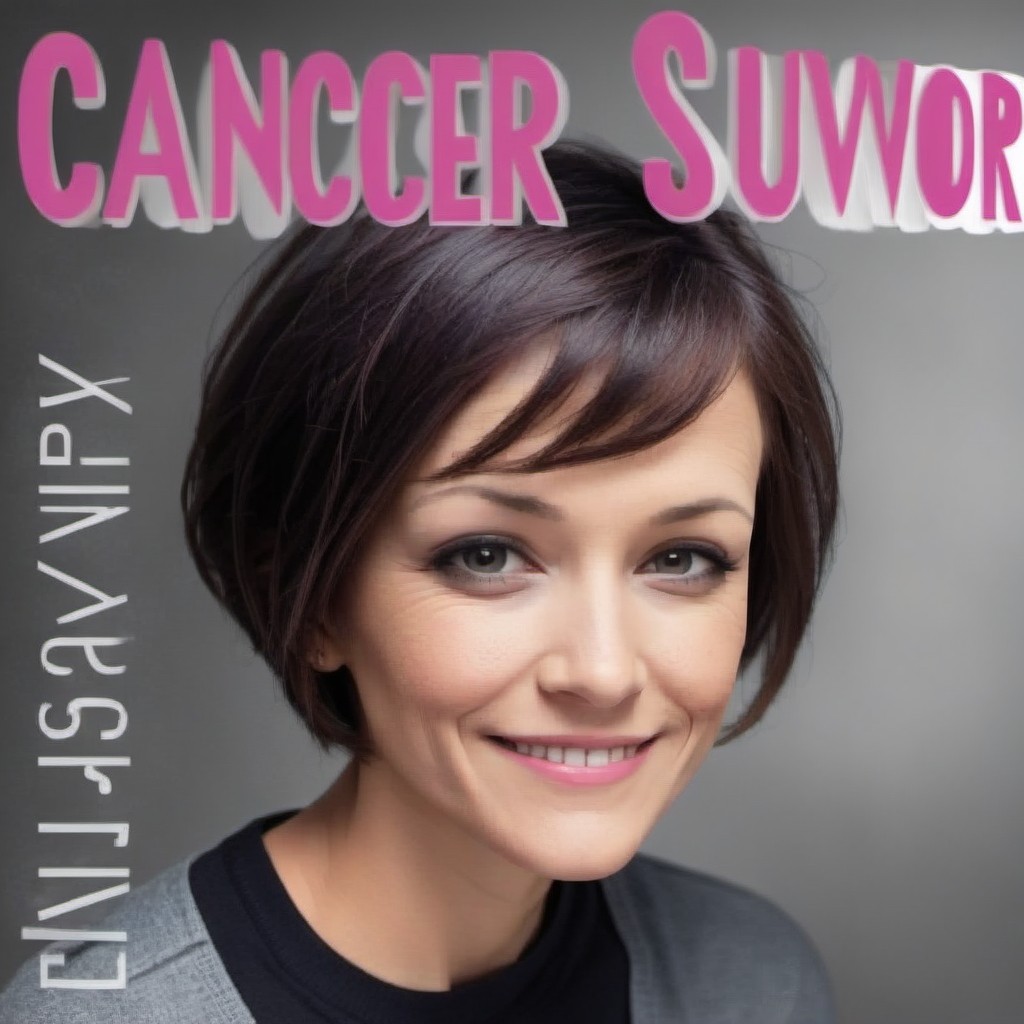

Supplement: Supplementary file 1 — Supplementary file1 (ZIP 11162 KB) [file 11764_2025_1760_MOESM1_ESM.zip › Data Images/cancer survivor/Stable Diffusion/206.jpg]

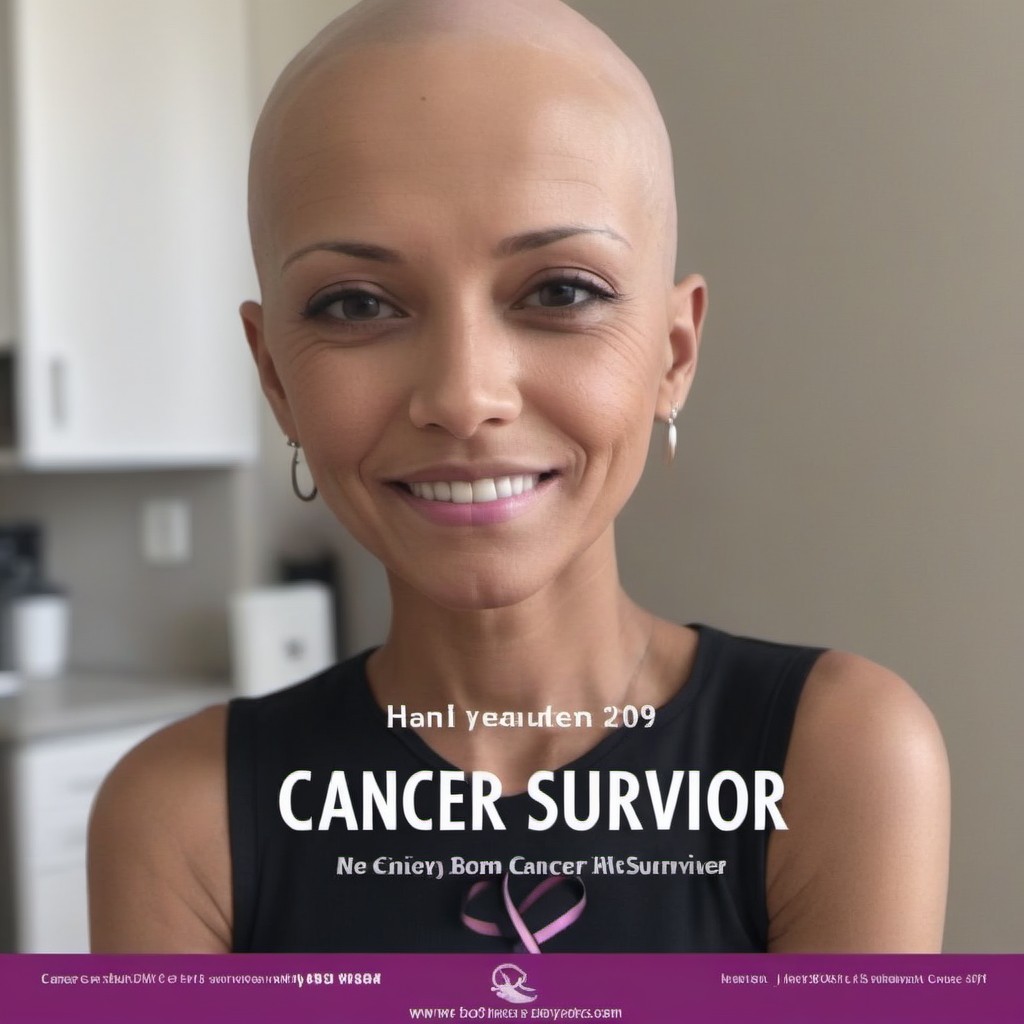

Supplement: Supplementary file 1 — Supplementary file1 (ZIP 11162 KB) [file 11764_2025_1760_MOESM1_ESM.zip › Data Images/cancer survivor/Stable Diffusion/207.jpg]

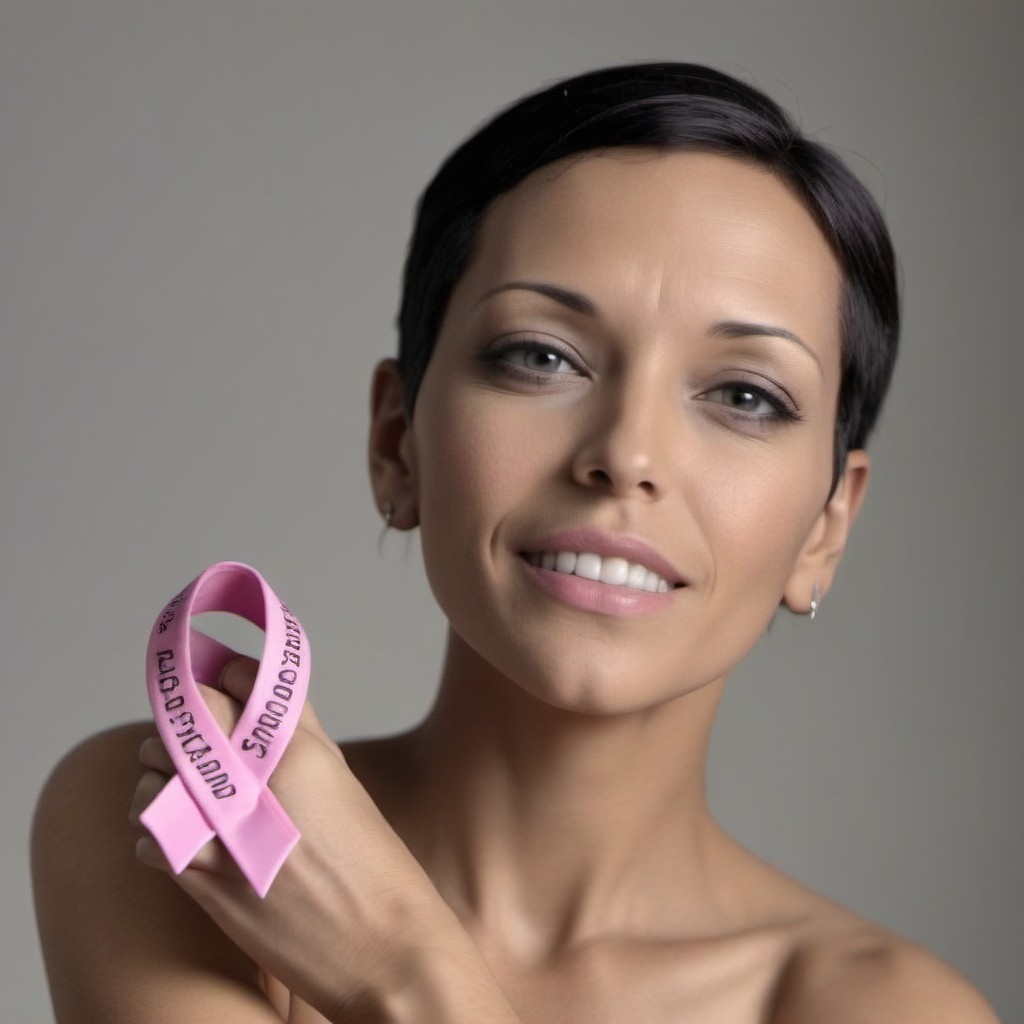

Supplement: Supplementary file 1 — Supplementary file1 (ZIP 11162 KB) [file 11764_2025_1760_MOESM1_ESM.zip › Data Images/cancer survivor/Stable Diffusion/208.jpg]

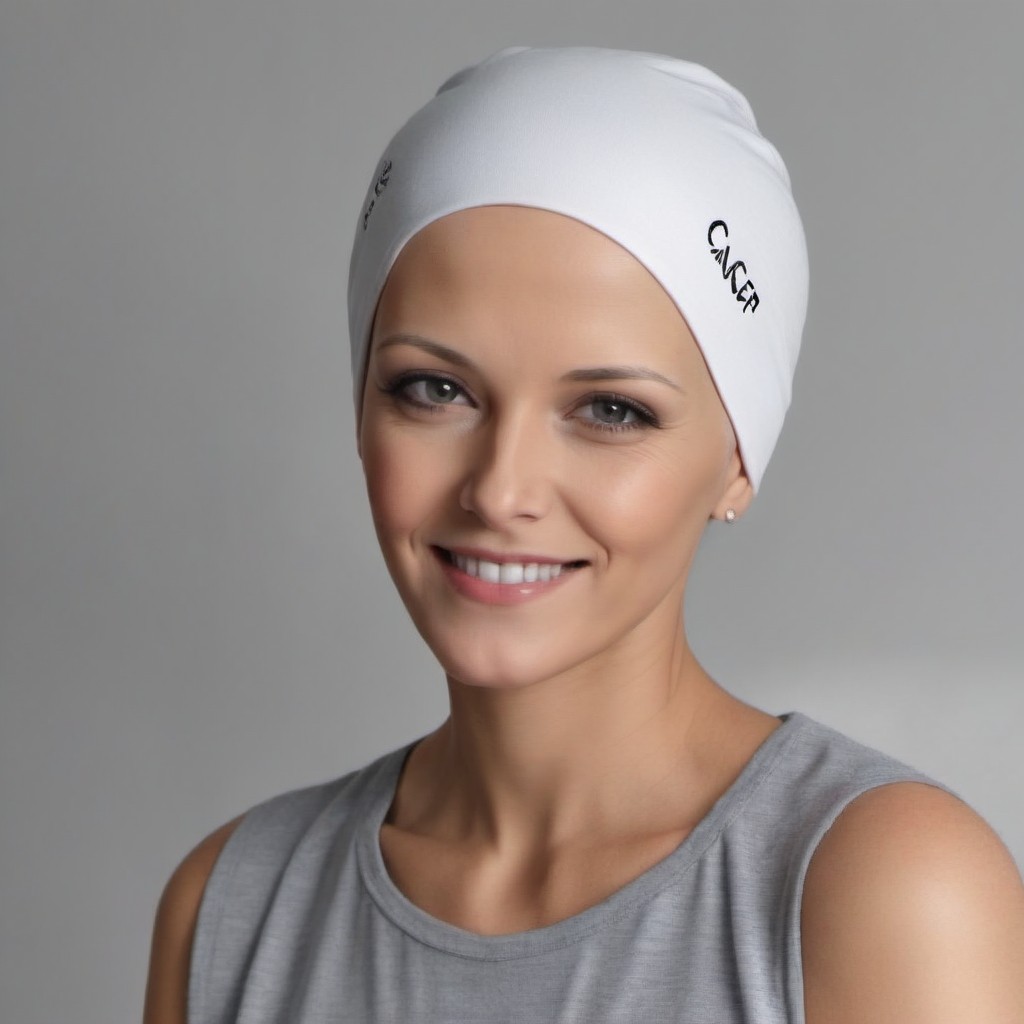

Supplement: Supplementary file 1 — Supplementary file1 (ZIP 11162 KB) [file 11764_2025_1760_MOESM1_ESM.zip › Data Images/cancer survivor/Stable Diffusion/209.jpg]

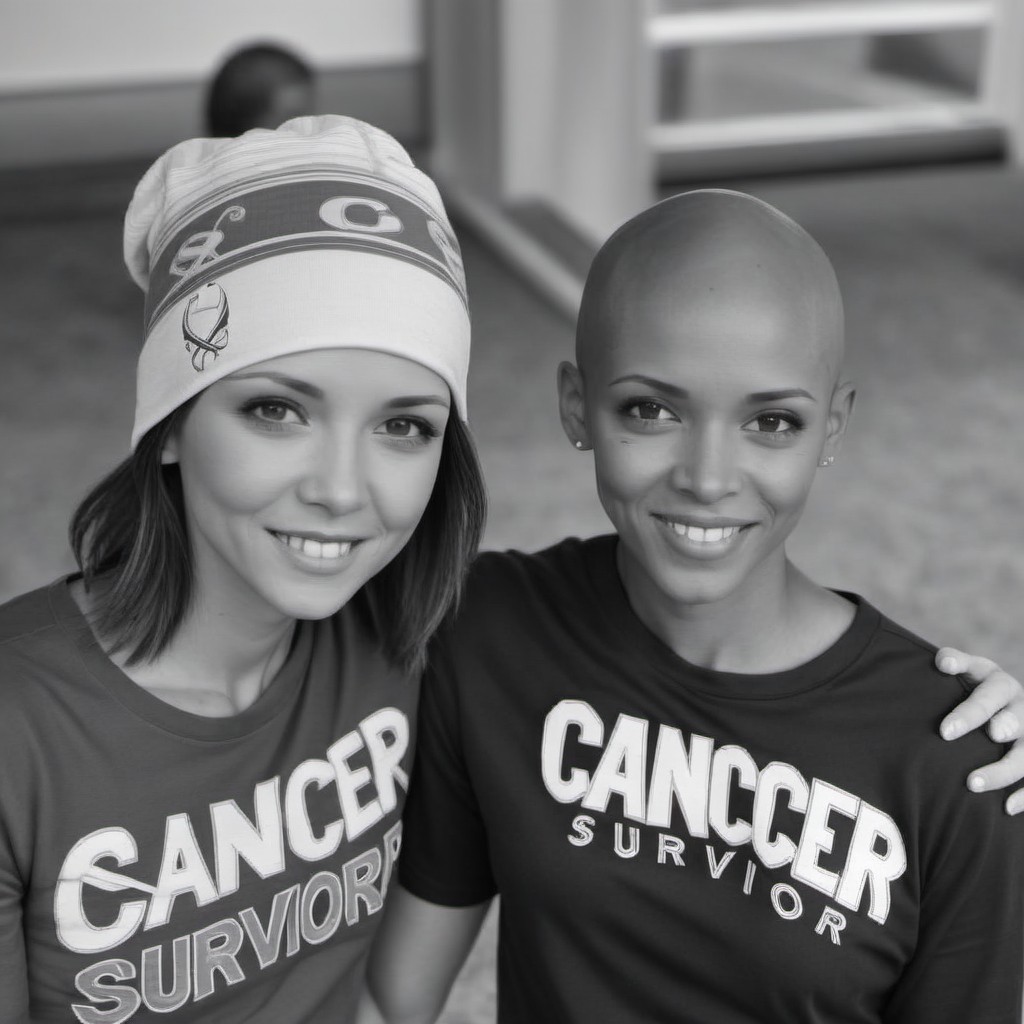

Supplement: Supplementary file 1 — Supplementary file1 (ZIP 11162 KB) [file 11764_2025_1760_MOESM1_ESM.zip › Data Images/cancer survivor/Stable Diffusion/210.jpg]

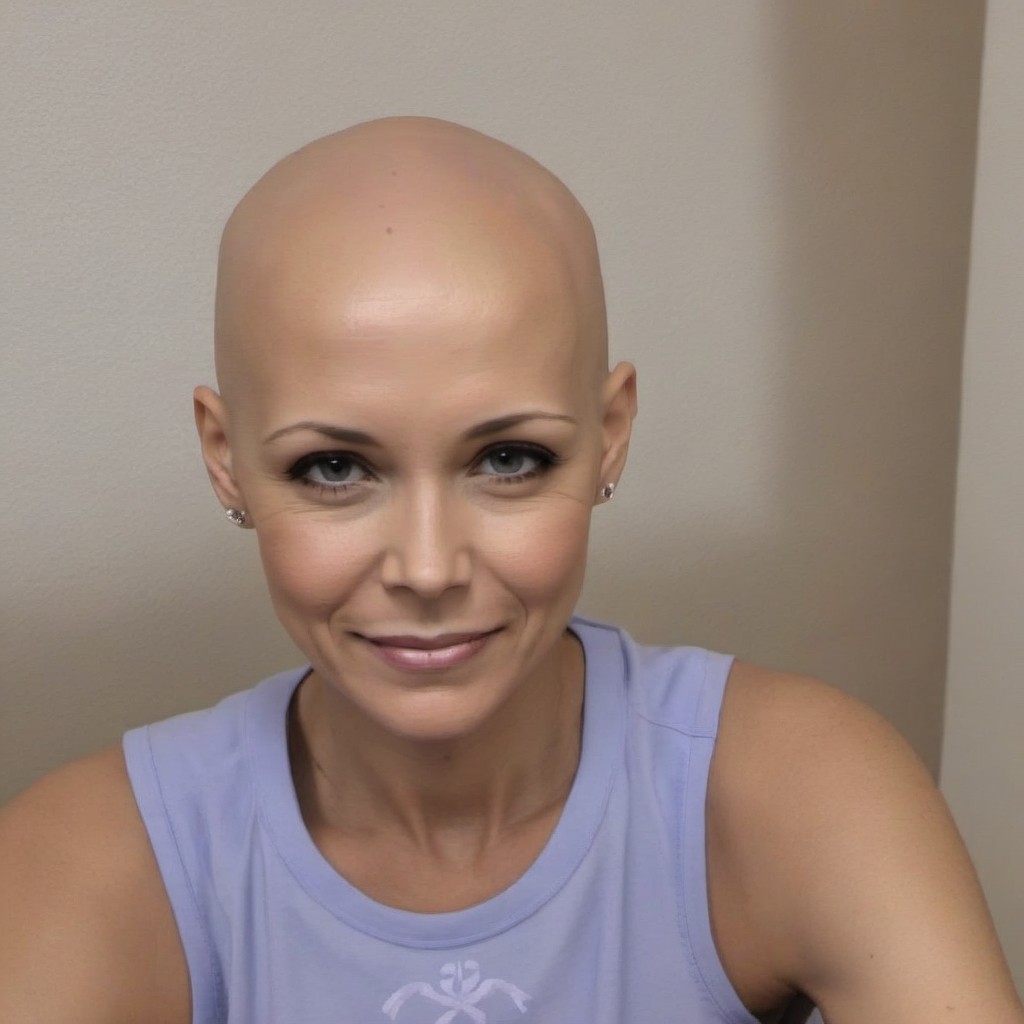

Supplement: Supplementary file 1 — Supplementary file1 (ZIP 11162 KB) [file 11764_2025_1760_MOESM1_ESM.zip › Data Images/cancer survivor/Stable Diffusion/211.jpg]

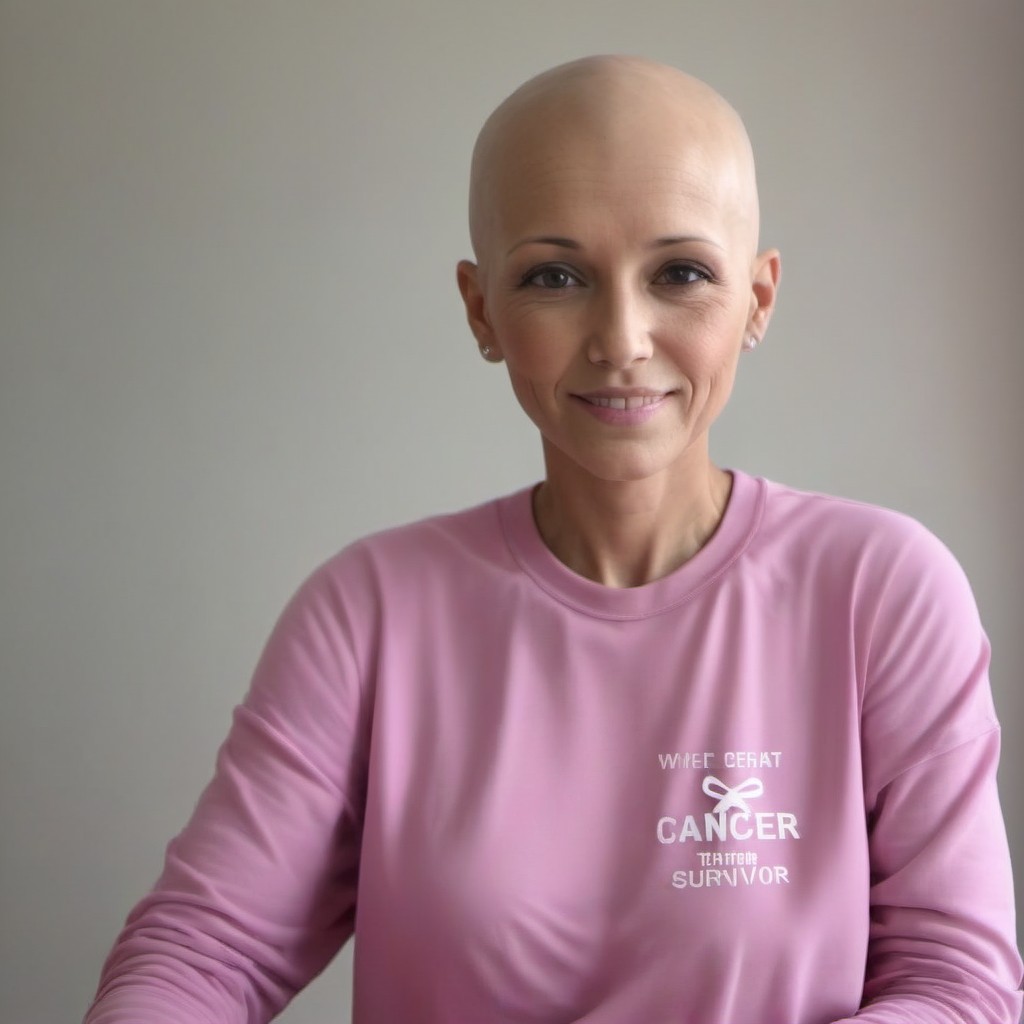

Supplement: Supplementary file 1 — Supplementary file1 (ZIP 11162 KB) [file 11764_2025_1760_MOESM1_ESM.zip › Data Images/cancer survivor/Stable Diffusion/212.jpg]

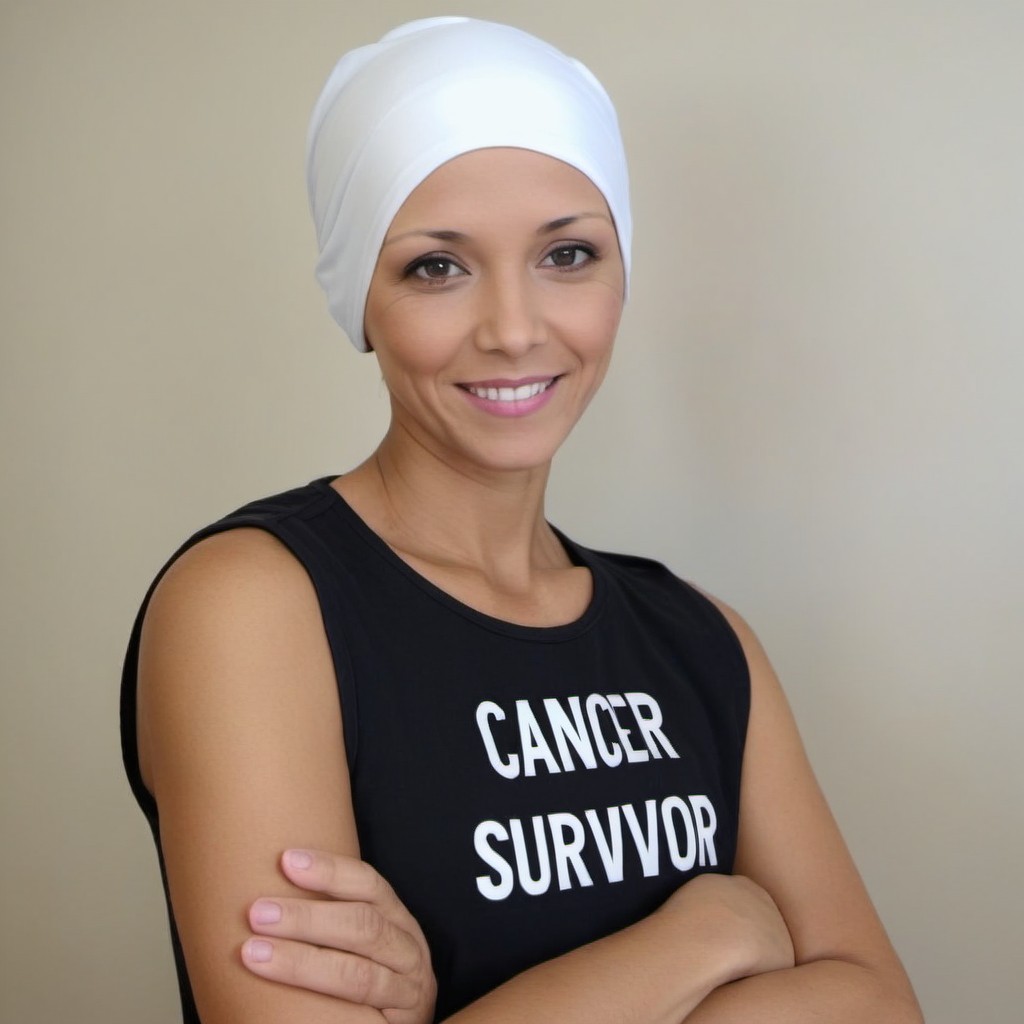

Supplement: Supplementary file 1 — Supplementary file1 (ZIP 11162 KB) [file 11764_2025_1760_MOESM1_ESM.zip › Data Images/cancer survivor/Stable Diffusion/213.jpg]

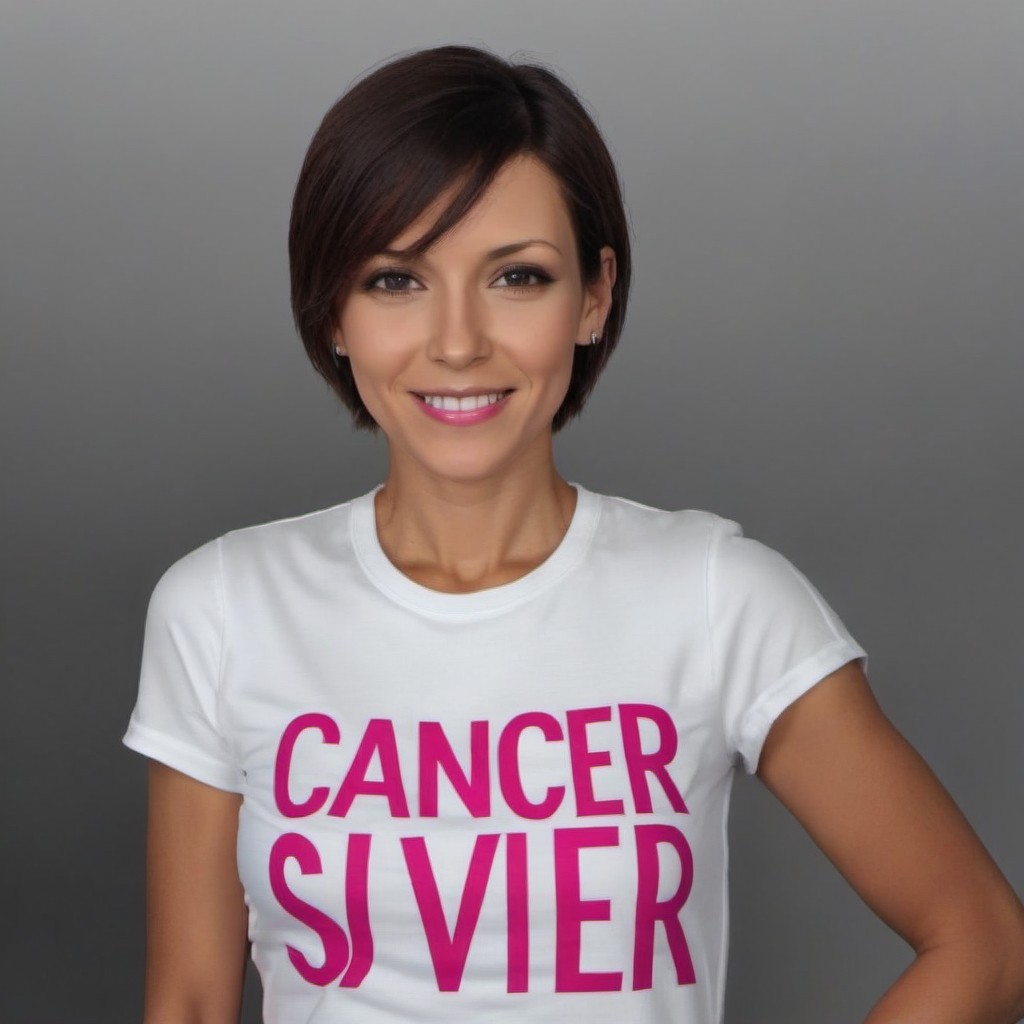

Supplement: Supplementary file 1 — Supplementary file1 (ZIP 11162 KB) [file 11764_2025_1760_MOESM1_ESM.zip › Data Images/cancer survivor/Stable Diffusion/214.jpg]

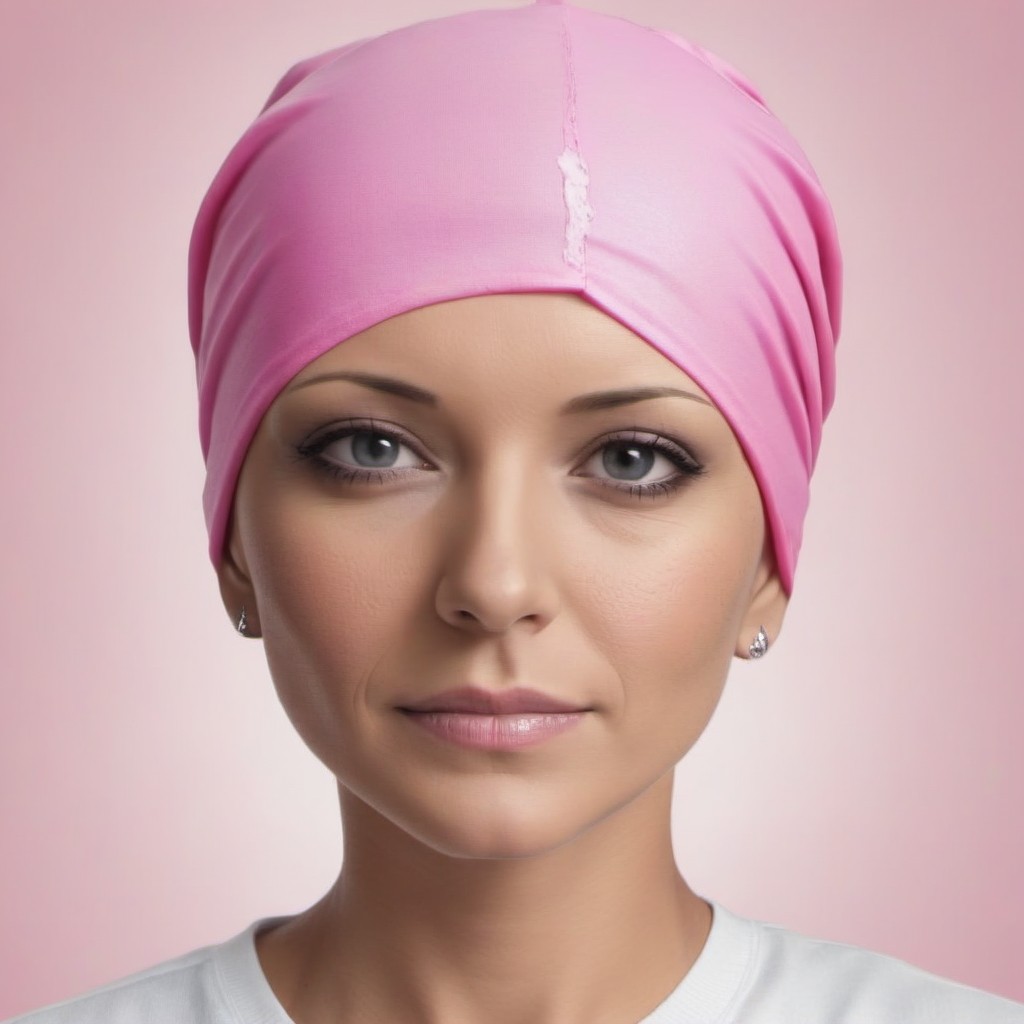

Supplement: Supplementary file 1 — Supplementary file1 (ZIP 11162 KB) [file 11764_2025_1760_MOESM1_ESM.zip › Data Images/cancer survivor/Stable Diffusion/215.jpg]

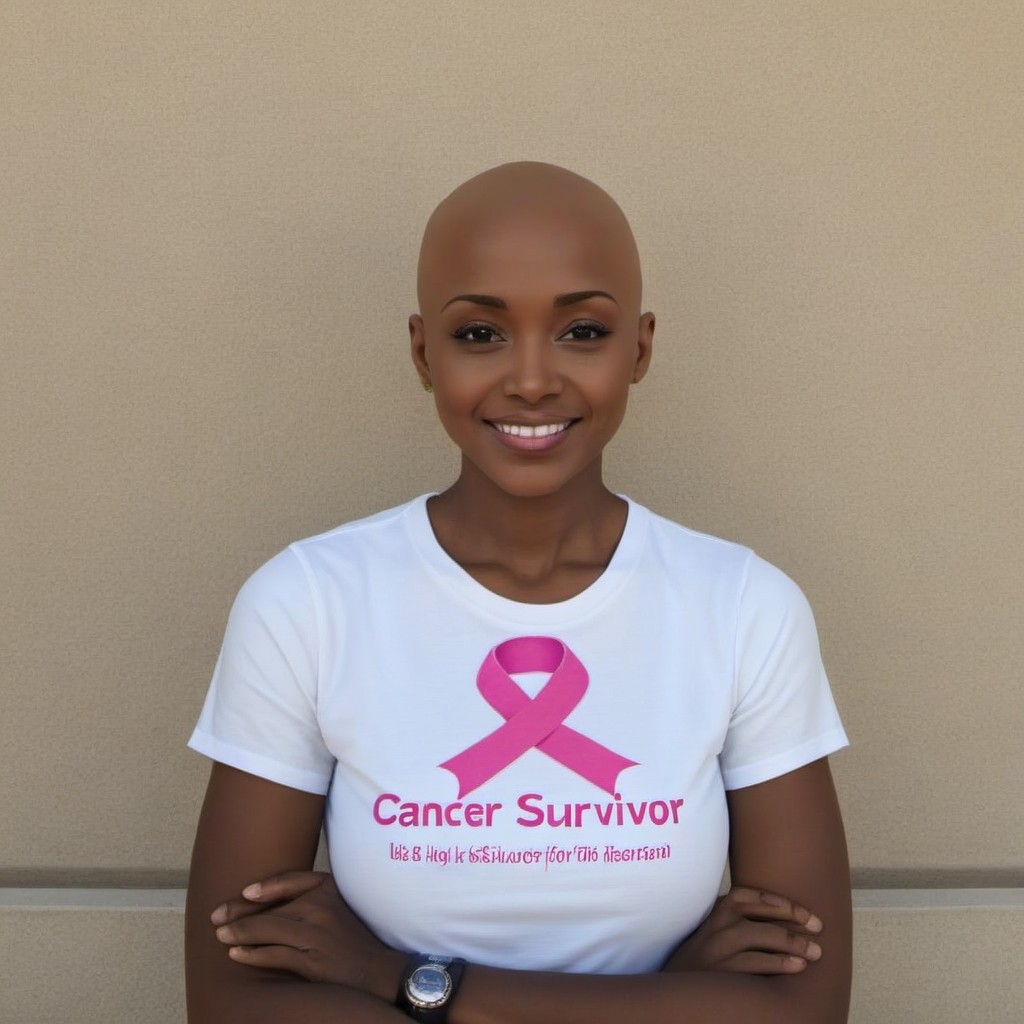

Supplement: Supplementary file 1 — Supplementary file1 (ZIP 11162 KB) [file 11764_2025_1760_MOESM1_ESM.zip › Data Images/cancer survivor/Stable Diffusion/216.jpg]

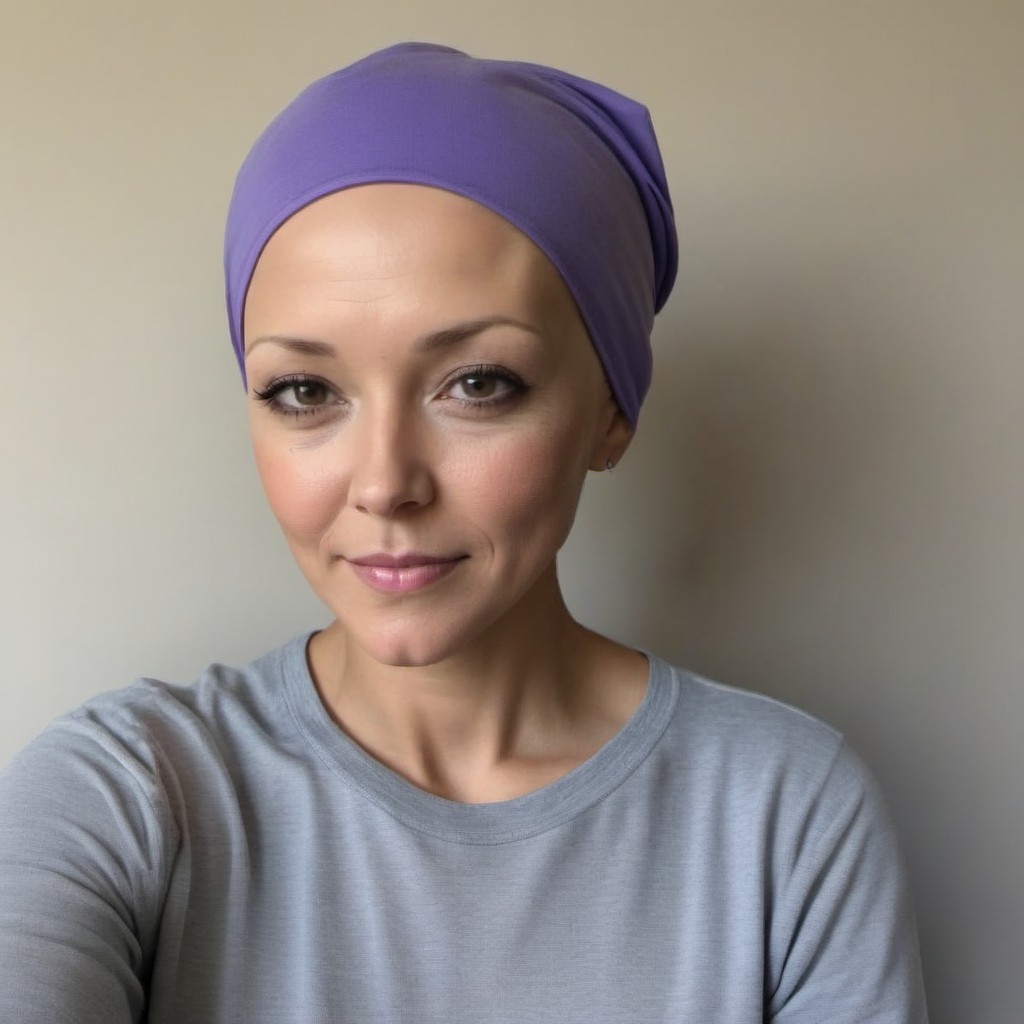

Supplement: Supplementary file 1 — Supplementary file1 (ZIP 11162 KB) [file 11764_2025_1760_MOESM1_ESM.zip › Data Images/cancer survivor/Stable Diffusion/217.jpg]

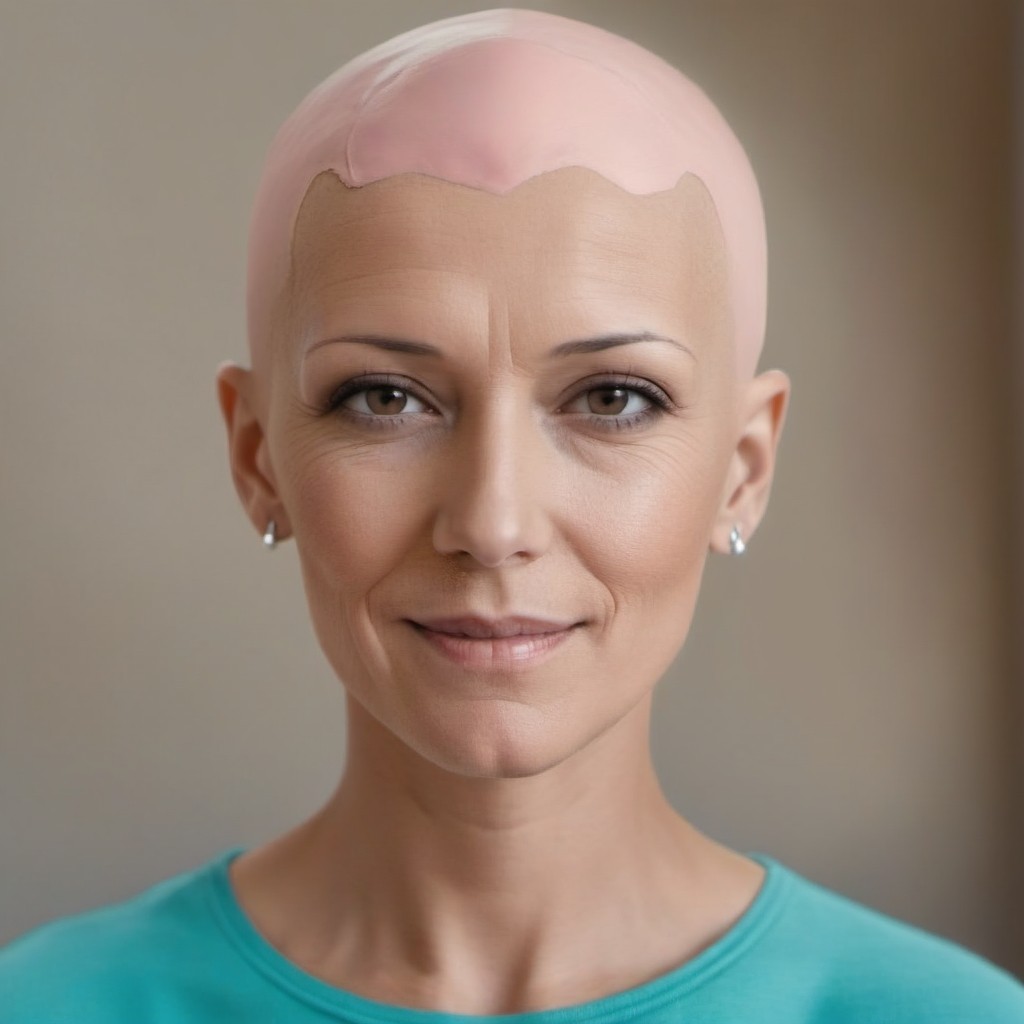

Supplement: Supplementary file 1 — Supplementary file1 (ZIP 11162 KB) [file 11764_2025_1760_MOESM1_ESM.zip › Data Images/cancer survivor/Stable Diffusion/218.jpg]

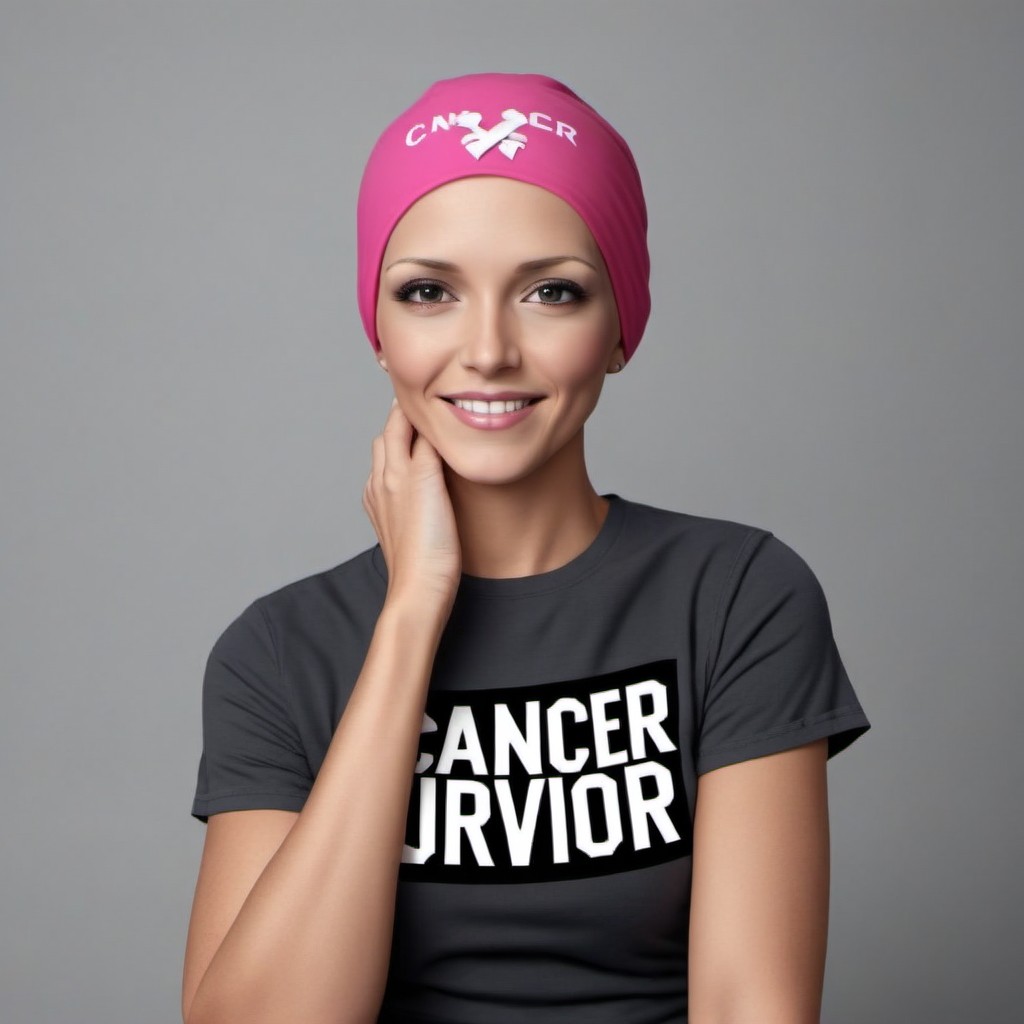

Supplement: Supplementary file 1 — Supplementary file1 (ZIP 11162 KB) [file 11764_2025_1760_MOESM1_ESM.zip › Data Images/cancer survivor/Stable Diffusion/219.jpg]

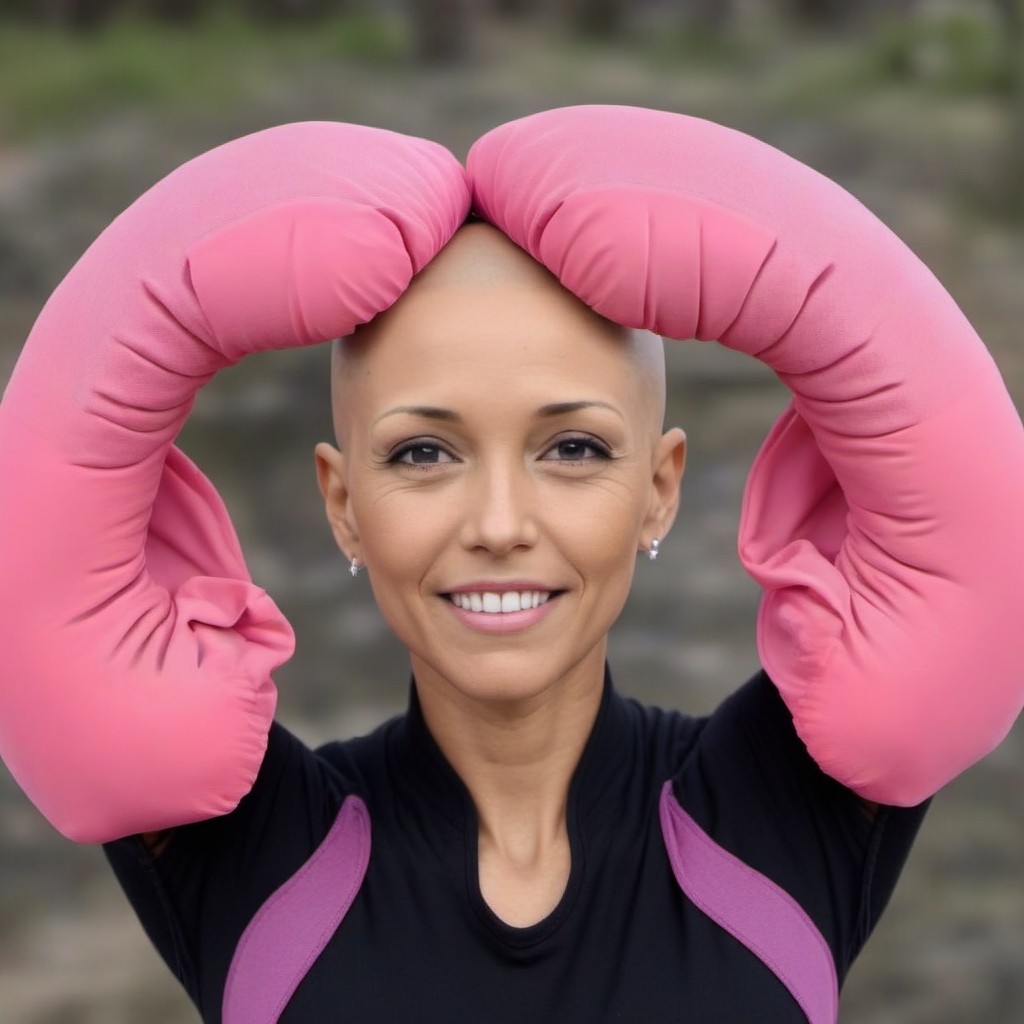

Supplement: Supplementary file 1 — Supplementary file1 (ZIP 11162 KB) [file 11764_2025_1760_MOESM1_ESM.zip › Data Images/cancer survivor/Stable Diffusion/220.jpg]
