# Supplementary figures and images for: What does an AI-generated “cancer survivor” look like? An analysis of images generated by text-to-image tools (part 1 of 2)
Source: J Cancer Surviv. 2025 Mar 1;20(4):1612–21. doi: 10.1007/s11764-025-01760-1 (PMC13375691; doi:10.1007/s11764-025-01760-1)

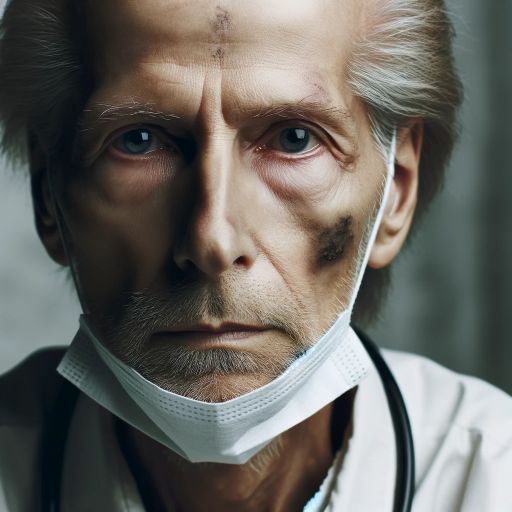

Supplement: Supplementary file 1 — Supplementary file1 (ZIP 11162 KB) [file 11764_2025_1760_MOESM1_ESM.zip › Data Images/cancer patient/ChatGPT/341.jpg]

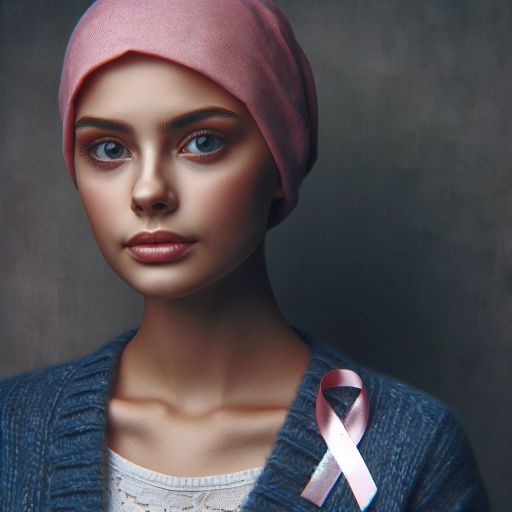

Supplement: Supplementary file 1 — Supplementary file1 (ZIP 11162 KB) [file 11764_2025_1760_MOESM1_ESM.zip › Data Images/cancer patient/ChatGPT/342.jpg]

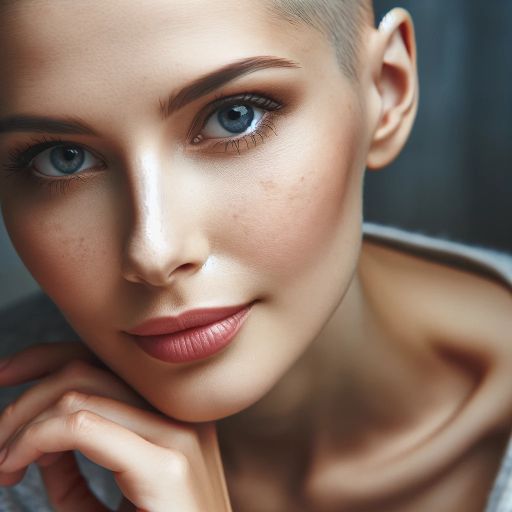

Supplement: Supplementary file 1 — Supplementary file1 (ZIP 11162 KB) [file 11764_2025_1760_MOESM1_ESM.zip › Data Images/cancer patient/ChatGPT/343.jpg]

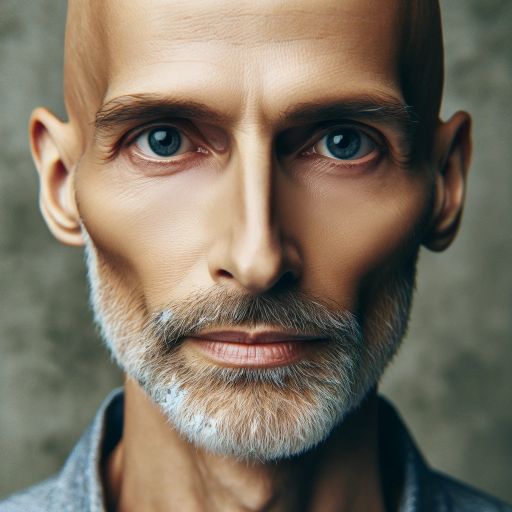

Supplement: Supplementary file 1 — Supplementary file1 (ZIP 11162 KB) [file 11764_2025_1760_MOESM1_ESM.zip › Data Images/cancer patient/ChatGPT/344.jpg]

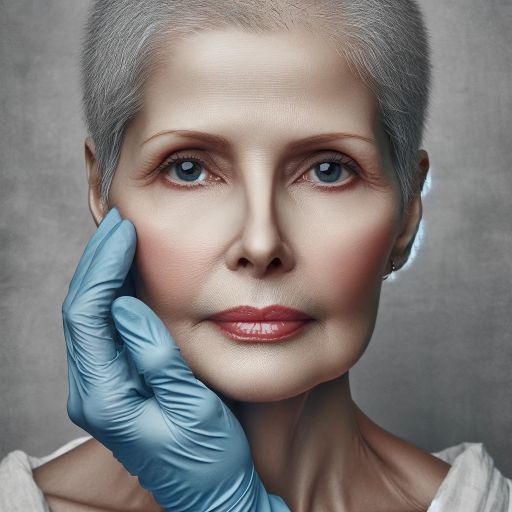

Supplement: Supplementary file 1 — Supplementary file1 (ZIP 11162 KB) [file 11764_2025_1760_MOESM1_ESM.zip › Data Images/cancer patient/ChatGPT/345.jpg]

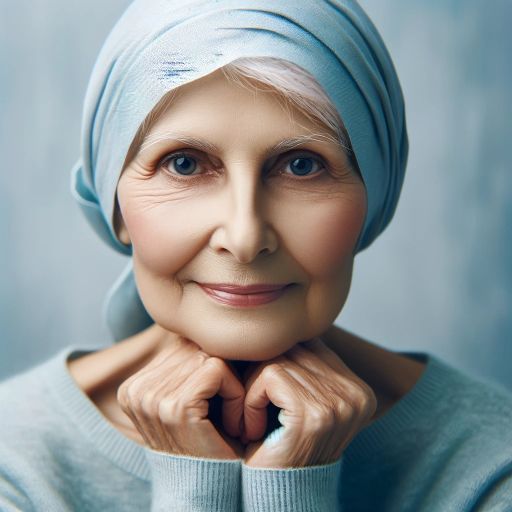

Supplement: Supplementary file 1 — Supplementary file1 (ZIP 11162 KB) [file 11764_2025_1760_MOESM1_ESM.zip › Data Images/cancer patient/ChatGPT/346.jpg]

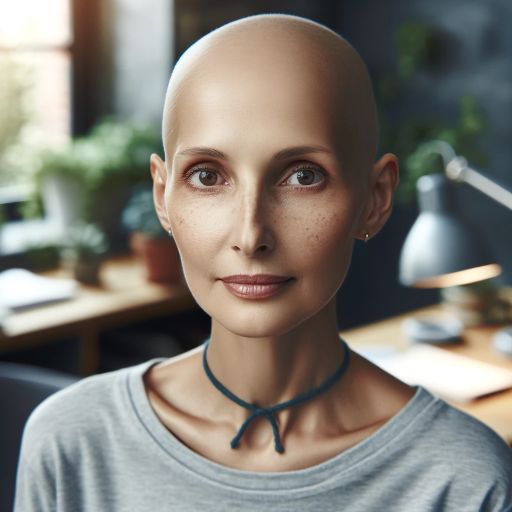

Supplement: Supplementary file 1 — Supplementary file1 (ZIP 11162 KB) [file 11764_2025_1760_MOESM1_ESM.zip › Data Images/cancer patient/ChatGPT/347.jpg]

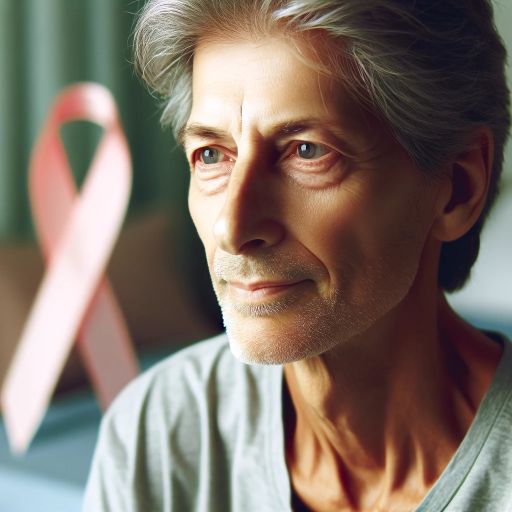

Supplement: Supplementary file 1 — Supplementary file1 (ZIP 11162 KB) [file 11764_2025_1760_MOESM1_ESM.zip › Data Images/cancer patient/ChatGPT/348.jpg]

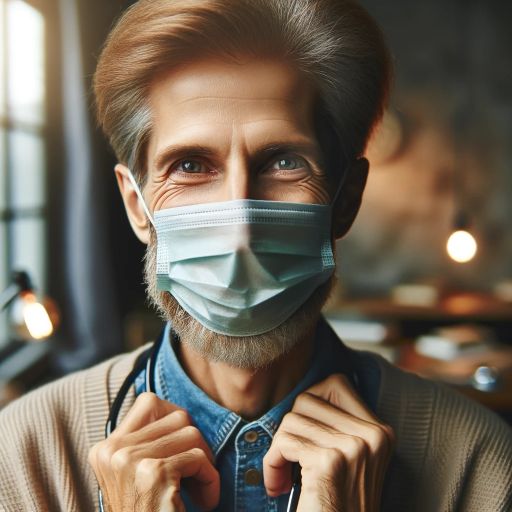

Supplement: Supplementary file 1 — Supplementary file1 (ZIP 11162 KB) [file 11764_2025_1760_MOESM1_ESM.zip › Data Images/cancer patient/ChatGPT/349.jpg]

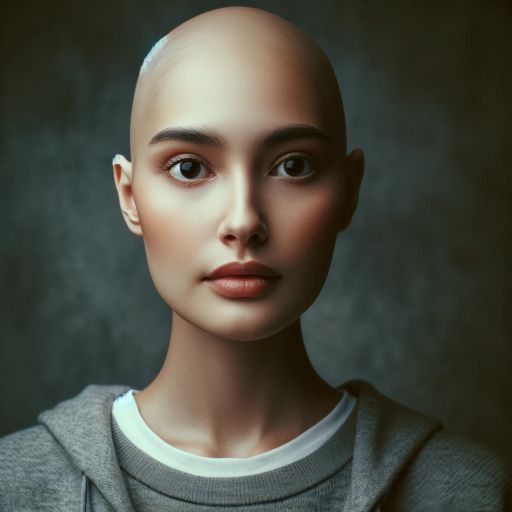

Supplement: Supplementary file 1 — Supplementary file1 (ZIP 11162 KB) [file 11764_2025_1760_MOESM1_ESM.zip › Data Images/cancer patient/ChatGPT/350.jpg]

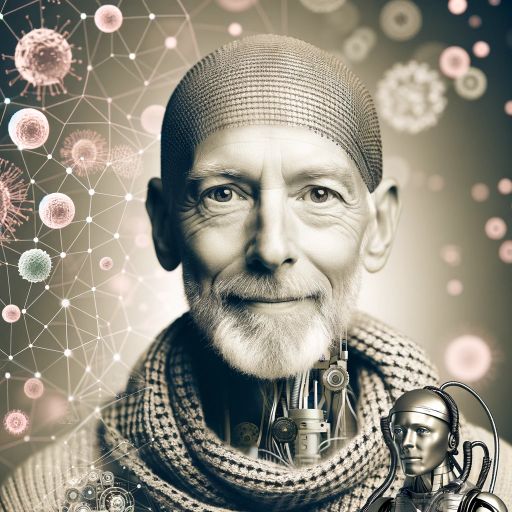

Supplement: Supplementary file 1 — Supplementary file1 (ZIP 11162 KB) [file 11764_2025_1760_MOESM1_ESM.zip › Data Images/cancer patient/ChatGPT/351.jpg]

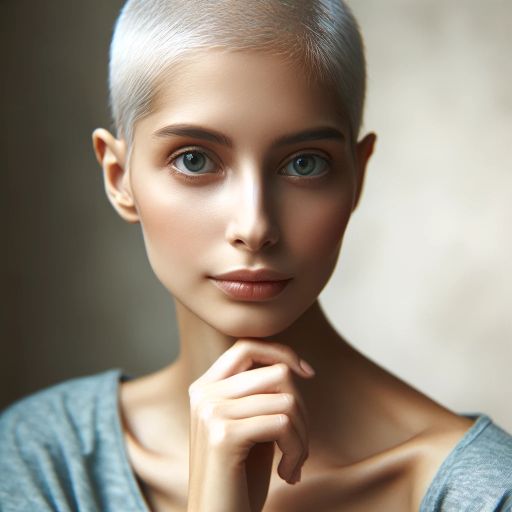

Supplement: Supplementary file 1 — Supplementary file1 (ZIP 11162 KB) [file 11764_2025_1760_MOESM1_ESM.zip › Data Images/cancer patient/ChatGPT/352.jpg]

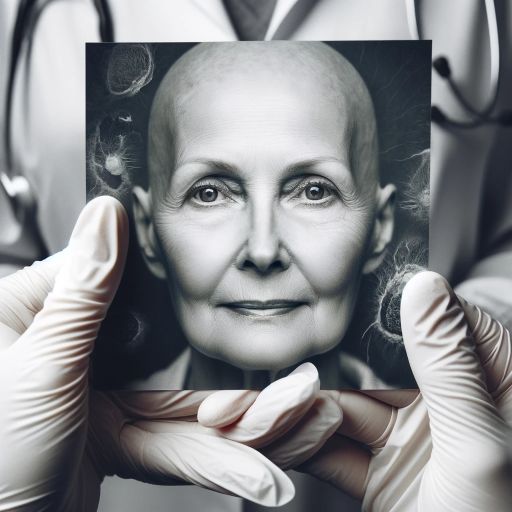

Supplement: Supplementary file 1 — Supplementary file1 (ZIP 11162 KB) [file 11764_2025_1760_MOESM1_ESM.zip › Data Images/cancer patient/ChatGPT/353.jpg]

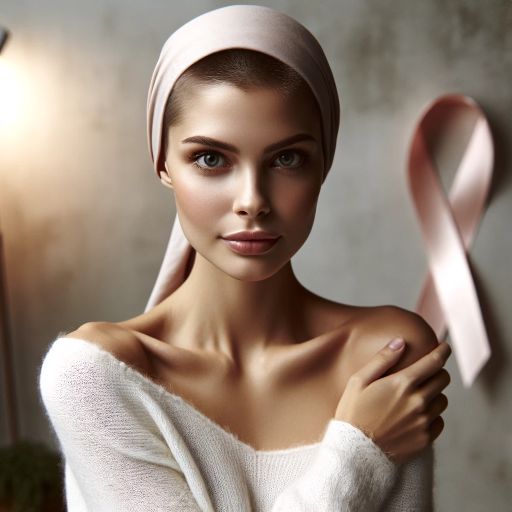

Supplement: Supplementary file 1 — Supplementary file1 (ZIP 11162 KB) [file 11764_2025_1760_MOESM1_ESM.zip › Data Images/cancer patient/ChatGPT/354.jpg]

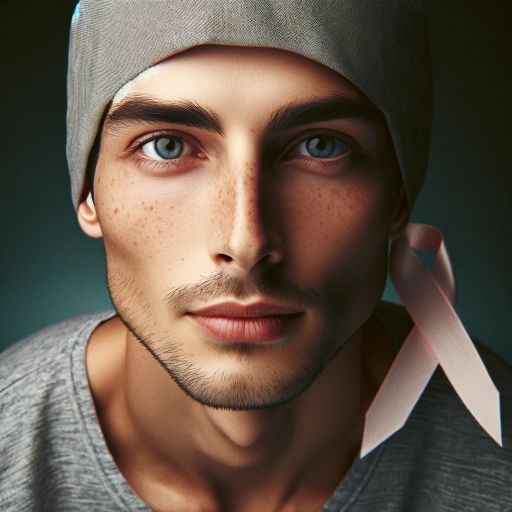

Supplement: Supplementary file 1 — Supplementary file1 (ZIP 11162 KB) [file 11764_2025_1760_MOESM1_ESM.zip › Data Images/cancer patient/ChatGPT/355.jpg]

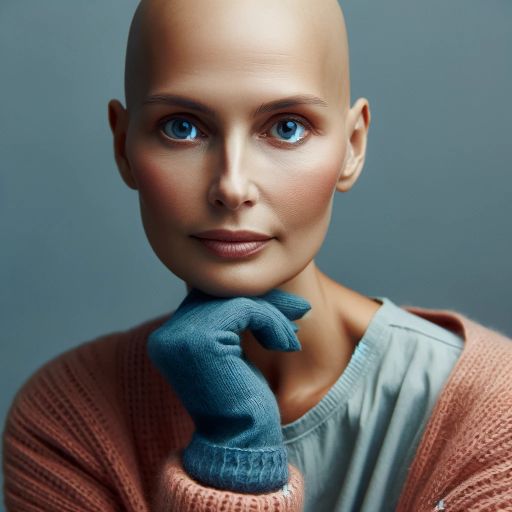

Supplement: Supplementary file 1 — Supplementary file1 (ZIP 11162 KB) [file 11764_2025_1760_MOESM1_ESM.zip › Data Images/cancer patient/ChatGPT/356.jpg]

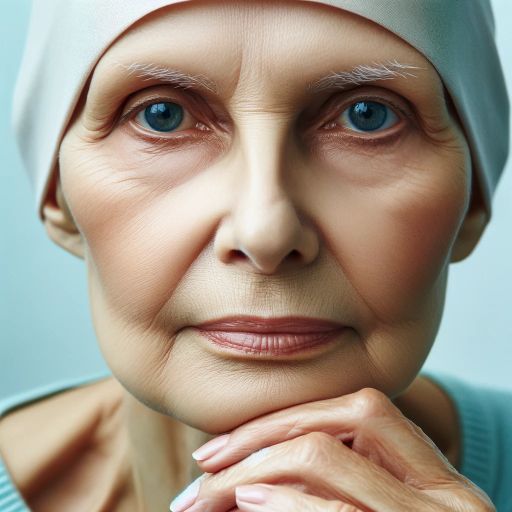

Supplement: Supplementary file 1 — Supplementary file1 (ZIP 11162 KB) [file 11764_2025_1760_MOESM1_ESM.zip › Data Images/cancer patient/ChatGPT/357.jpg]

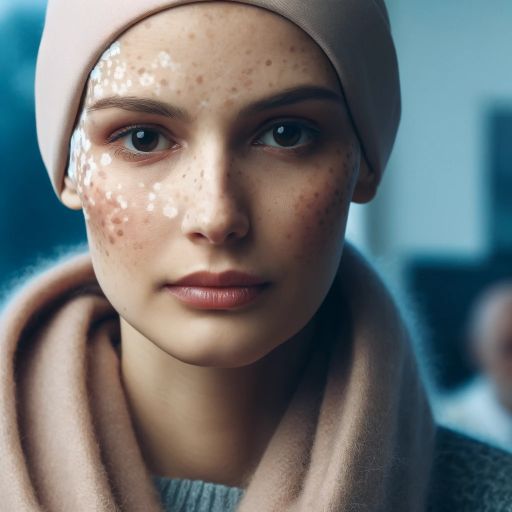

Supplement: Supplementary file 1 — Supplementary file1 (ZIP 11162 KB) [file 11764_2025_1760_MOESM1_ESM.zip › Data Images/cancer patient/ChatGPT/358.jpg]

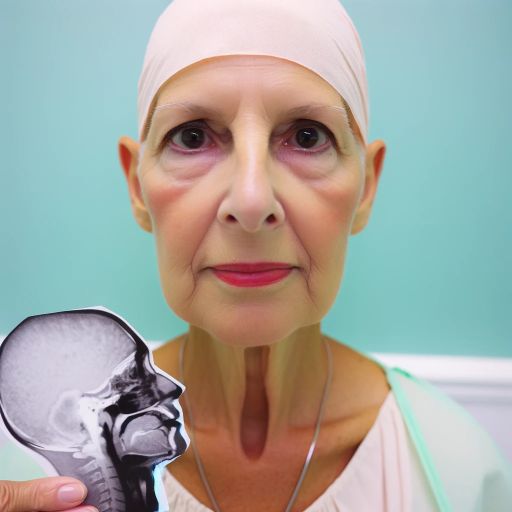

Supplement: Supplementary file 1 — Supplementary file1 (ZIP 11162 KB) [file 11764_2025_1760_MOESM1_ESM.zip › Data Images/cancer patient/ChatGPT/359.jpg]

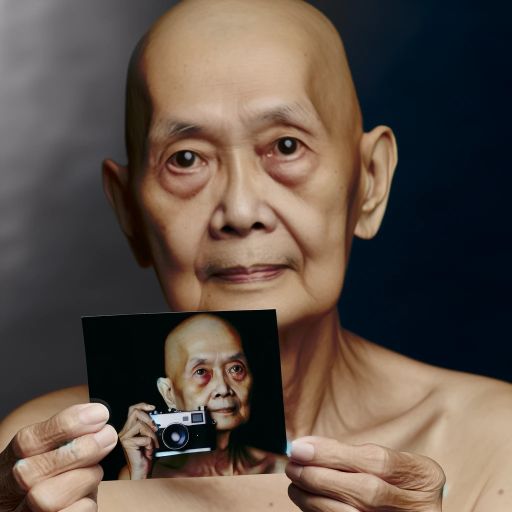

Supplement: Supplementary file 1 — Supplementary file1 (ZIP 11162 KB) [file 11764_2025_1760_MOESM1_ESM.zip › Data Images/cancer patient/ChatGPT/360.jpg]

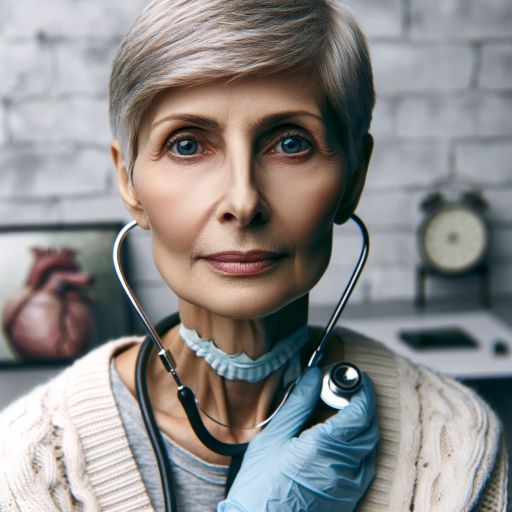

Supplement: Supplementary file 1 — Supplementary file1 (ZIP 11162 KB) [file 11764_2025_1760_MOESM1_ESM.zip › Data Images/cancer patient/ChatGPT/361.jpg]

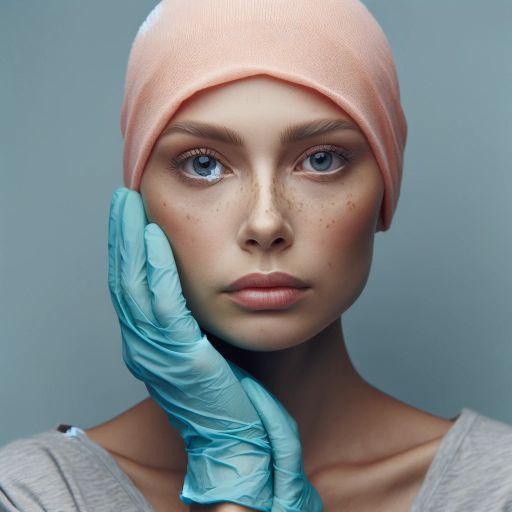

Supplement: Supplementary file 1 — Supplementary file1 (ZIP 11162 KB) [file 11764_2025_1760_MOESM1_ESM.zip › Data Images/cancer patient/ChatGPT/362.jpg]

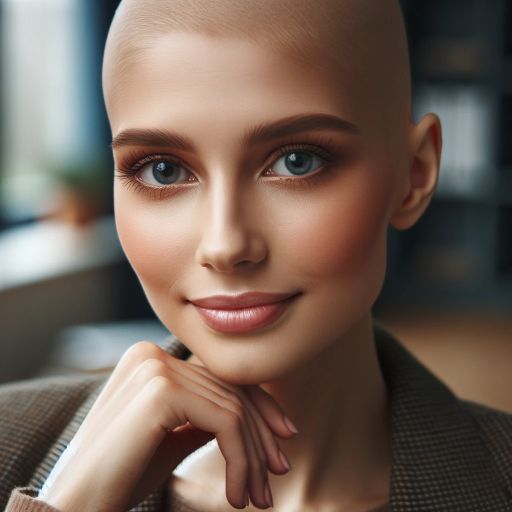

Supplement: Supplementary file 1 — Supplementary file1 (ZIP 11162 KB) [file 11764_2025_1760_MOESM1_ESM.zip › Data Images/cancer patient/ChatGPT/363.jpg]

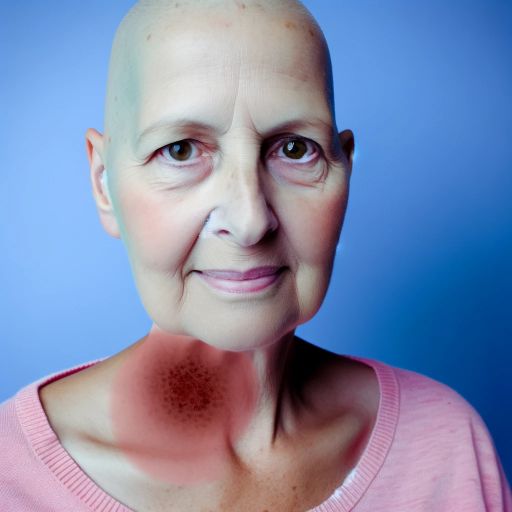

Supplement: Supplementary file 1 — Supplementary file1 (ZIP 11162 KB) [file 11764_2025_1760_MOESM1_ESM.zip › Data Images/cancer patient/ChatGPT/364.jpg]

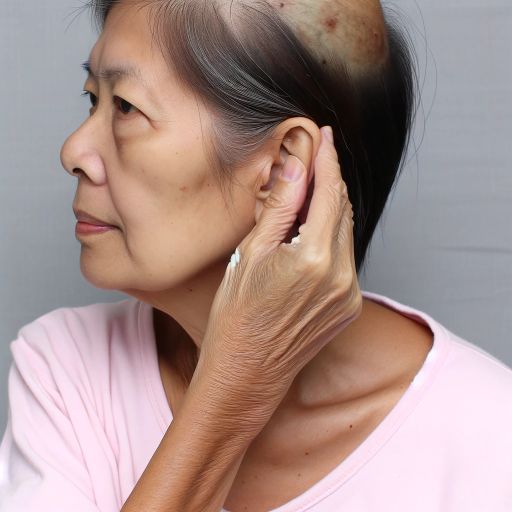

Supplement: Supplementary file 1 — Supplementary file1 (ZIP 11162 KB) [file 11764_2025_1760_MOESM1_ESM.zip › Data Images/cancer patient/ChatGPT/365.jpg]

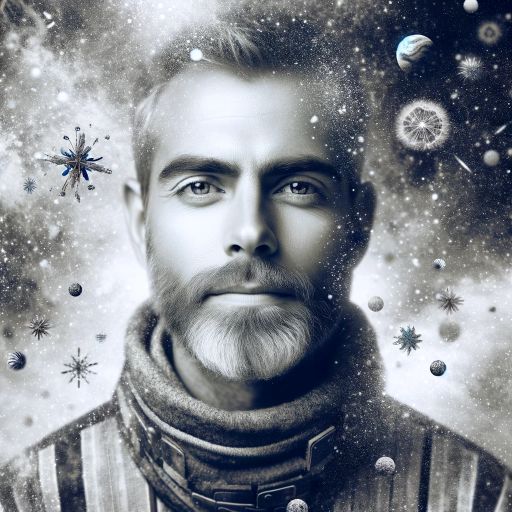

Supplement: Supplementary file 1 — Supplementary file1 (ZIP 11162 KB) [file 11764_2025_1760_MOESM1_ESM.zip › Data Images/cancer patient/ChatGPT/366.jpg]

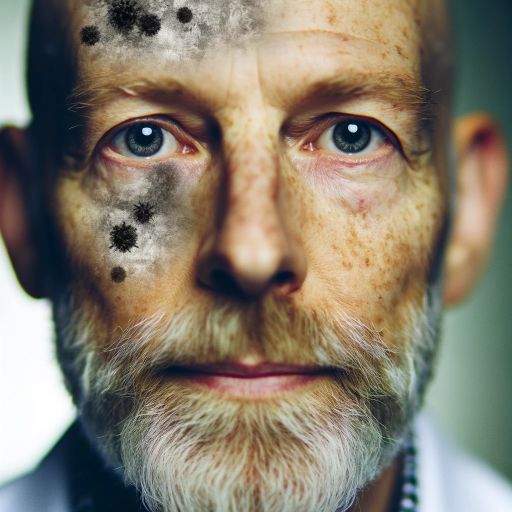

Supplement: Supplementary file 1 — Supplementary file1 (ZIP 11162 KB) [file 11764_2025_1760_MOESM1_ESM.zip › Data Images/cancer patient/ChatGPT/367.jpg]

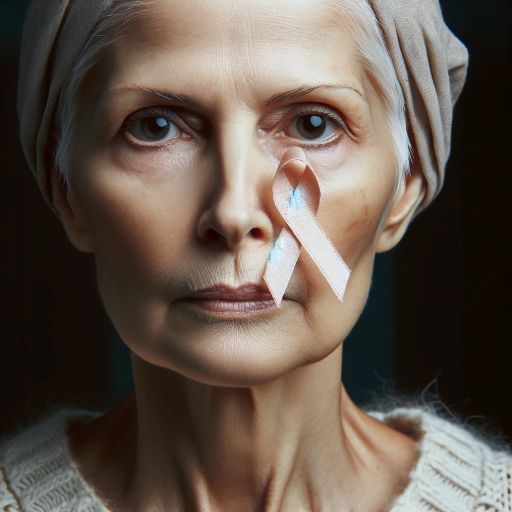

Supplement: Supplementary file 1 — Supplementary file1 (ZIP 11162 KB) [file 11764_2025_1760_MOESM1_ESM.zip › Data Images/cancer patient/ChatGPT/368.jpg]

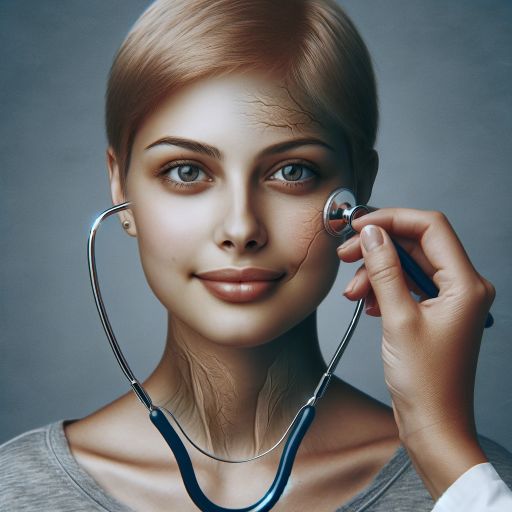

Supplement: Supplementary file 1 — Supplementary file1 (ZIP 11162 KB) [file 11764_2025_1760_MOESM1_ESM.zip › Data Images/cancer patient/ChatGPT/369.jpg]

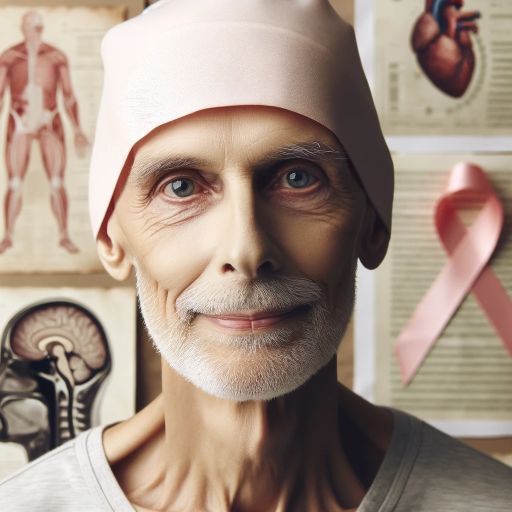

Supplement: Supplementary file 1 — Supplementary file1 (ZIP 11162 KB) [file 11764_2025_1760_MOESM1_ESM.zip › Data Images/cancer patient/ChatGPT/370.jpg]

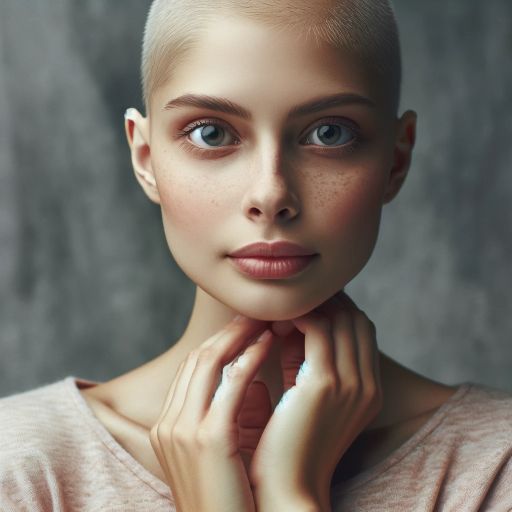

Supplement: Supplementary file 1 — Supplementary file1 (ZIP 11162 KB) [file 11764_2025_1760_MOESM1_ESM.zip › Data Images/cancer patient/ChatGPT/371.jpg]

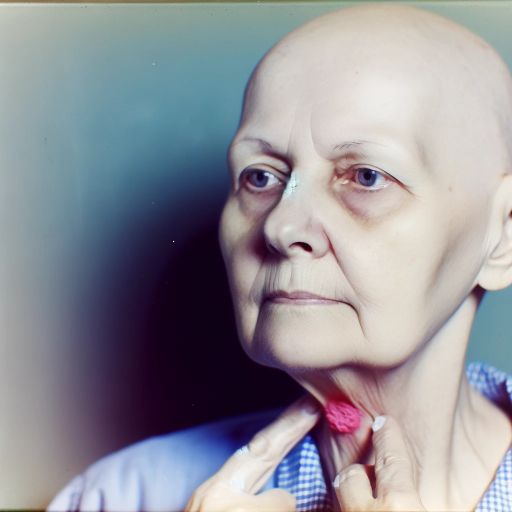

Supplement: Supplementary file 1 — Supplementary file1 (ZIP 11162 KB) [file 11764_2025_1760_MOESM1_ESM.zip › Data Images/cancer patient/ChatGPT/372.jpg]

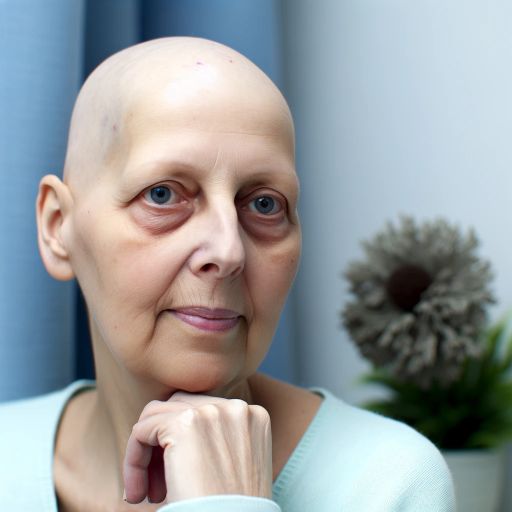

Supplement: Supplementary file 1 — Supplementary file1 (ZIP 11162 KB) [file 11764_2025_1760_MOESM1_ESM.zip › Data Images/cancer patient/ChatGPT/373.jpg]

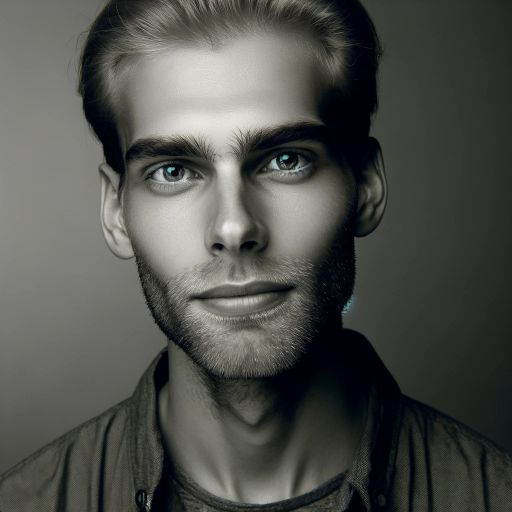

Supplement: Supplementary file 1 — Supplementary file1 (ZIP 11162 KB) [file 11764_2025_1760_MOESM1_ESM.zip › Data Images/cancer patient/ChatGPT/374.jpg]

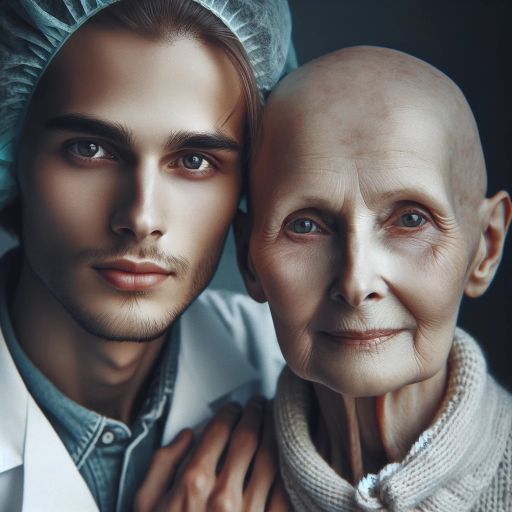

Supplement: Supplementary file 1 — Supplementary file1 (ZIP 11162 KB) [file 11764_2025_1760_MOESM1_ESM.zip › Data Images/cancer patient/ChatGPT/375.jpg]

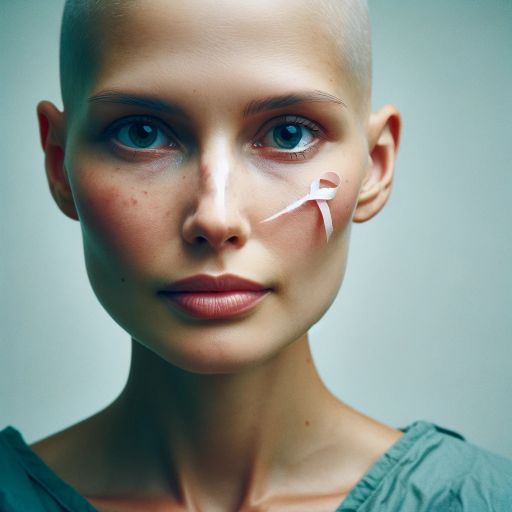

Supplement: Supplementary file 1 — Supplementary file1 (ZIP 11162 KB) [file 11764_2025_1760_MOESM1_ESM.zip › Data Images/cancer patient/ChatGPT/376.jpg]

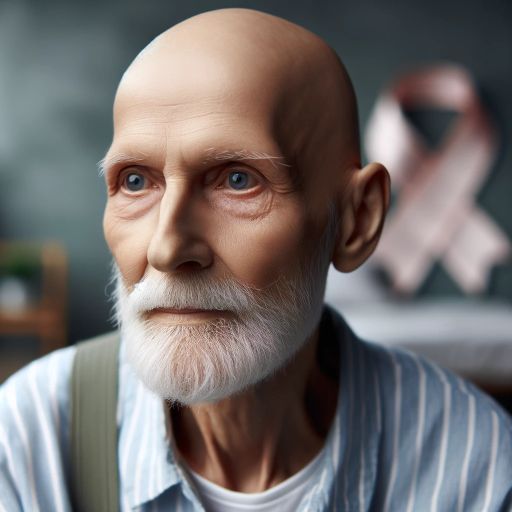

Supplement: Supplementary file 1 — Supplementary file1 (ZIP 11162 KB) [file 11764_2025_1760_MOESM1_ESM.zip › Data Images/cancer patient/ChatGPT/377.jpg]

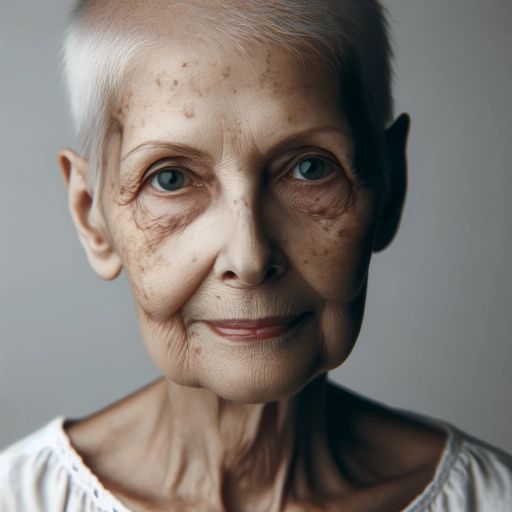

Supplement: Supplementary file 1 — Supplementary file1 (ZIP 11162 KB) [file 11764_2025_1760_MOESM1_ESM.zip › Data Images/cancer patient/ChatGPT/378.jpg]

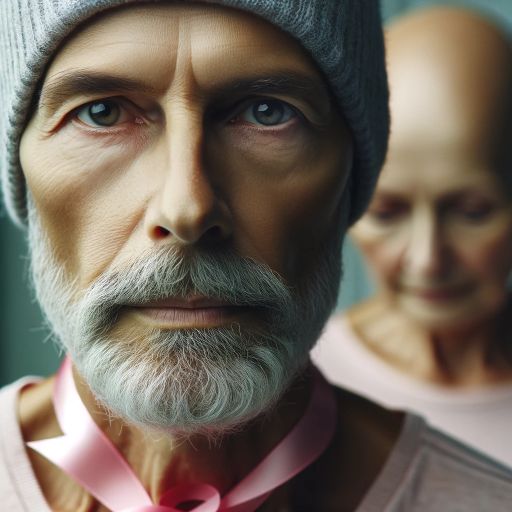

Supplement: Supplementary file 1 — Supplementary file1 (ZIP 11162 KB) [file 11764_2025_1760_MOESM1_ESM.zip › Data Images/cancer patient/ChatGPT/379.jpg]

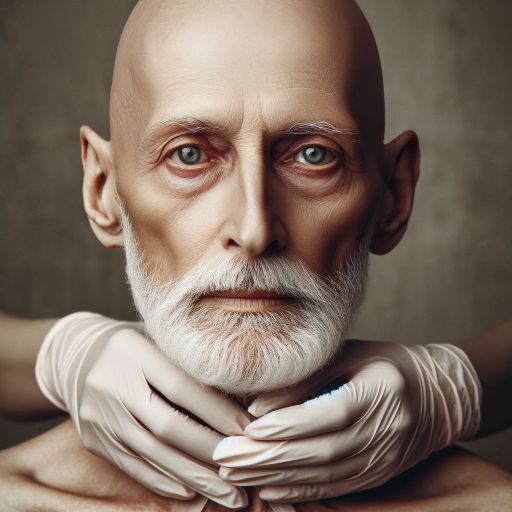

Supplement: Supplementary file 1 — Supplementary file1 (ZIP 11162 KB) [file 11764_2025_1760_MOESM1_ESM.zip › Data Images/cancer patient/ChatGPT/380.jpg]

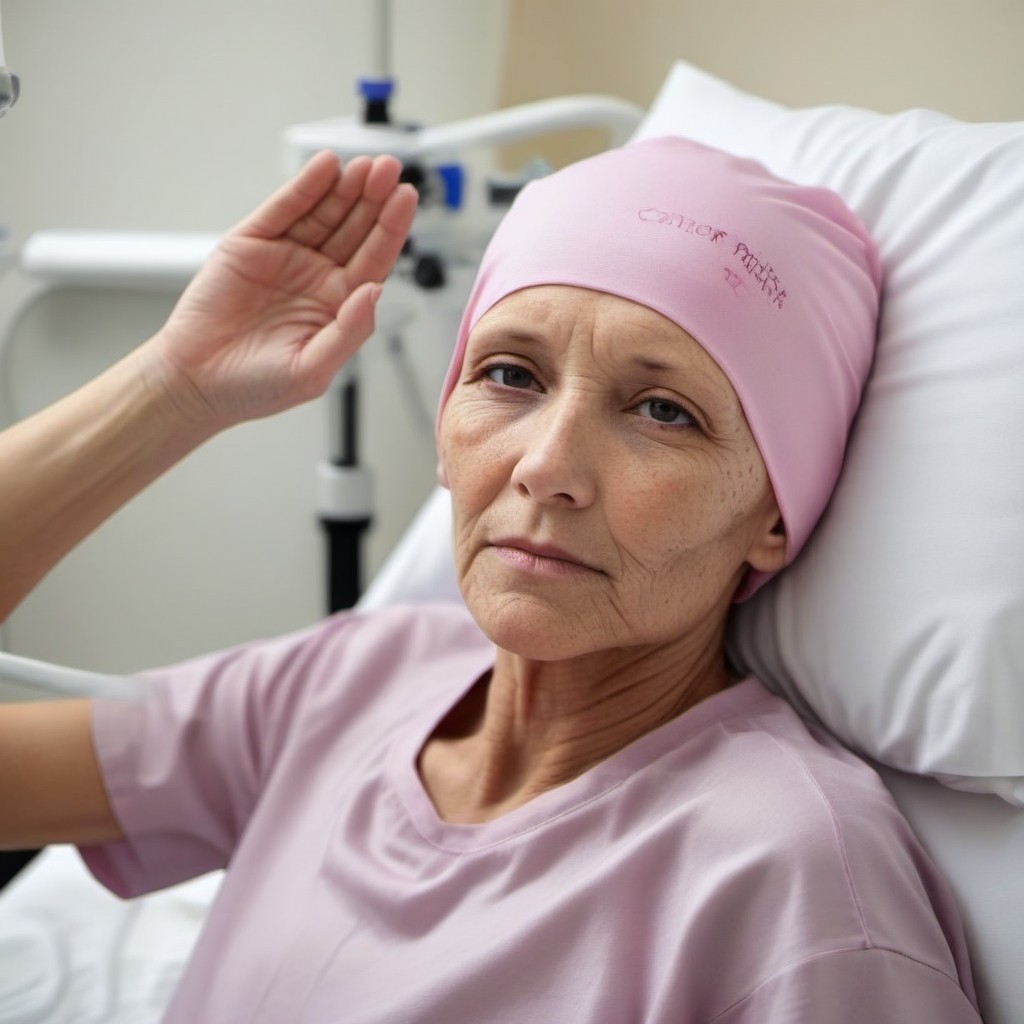

Supplement: Supplementary file 1 — Supplementary file1 (ZIP 11162 KB) [file 11764_2025_1760_MOESM1_ESM.zip › Data Images/cancer patient/Stable Diffusion/101.jpg]

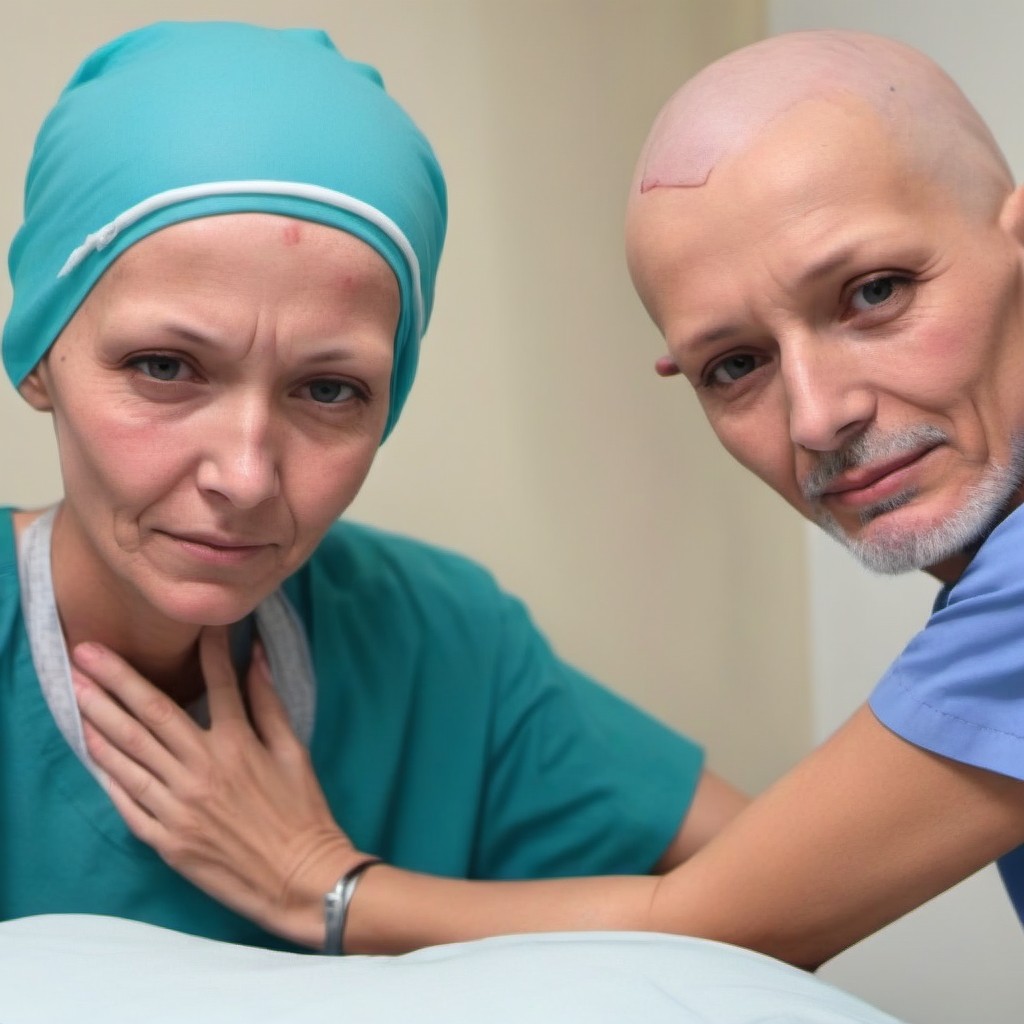

Supplement: Supplementary file 1 — Supplementary file1 (ZIP 11162 KB) [file 11764_2025_1760_MOESM1_ESM.zip › Data Images/cancer patient/Stable Diffusion/102.jpg]

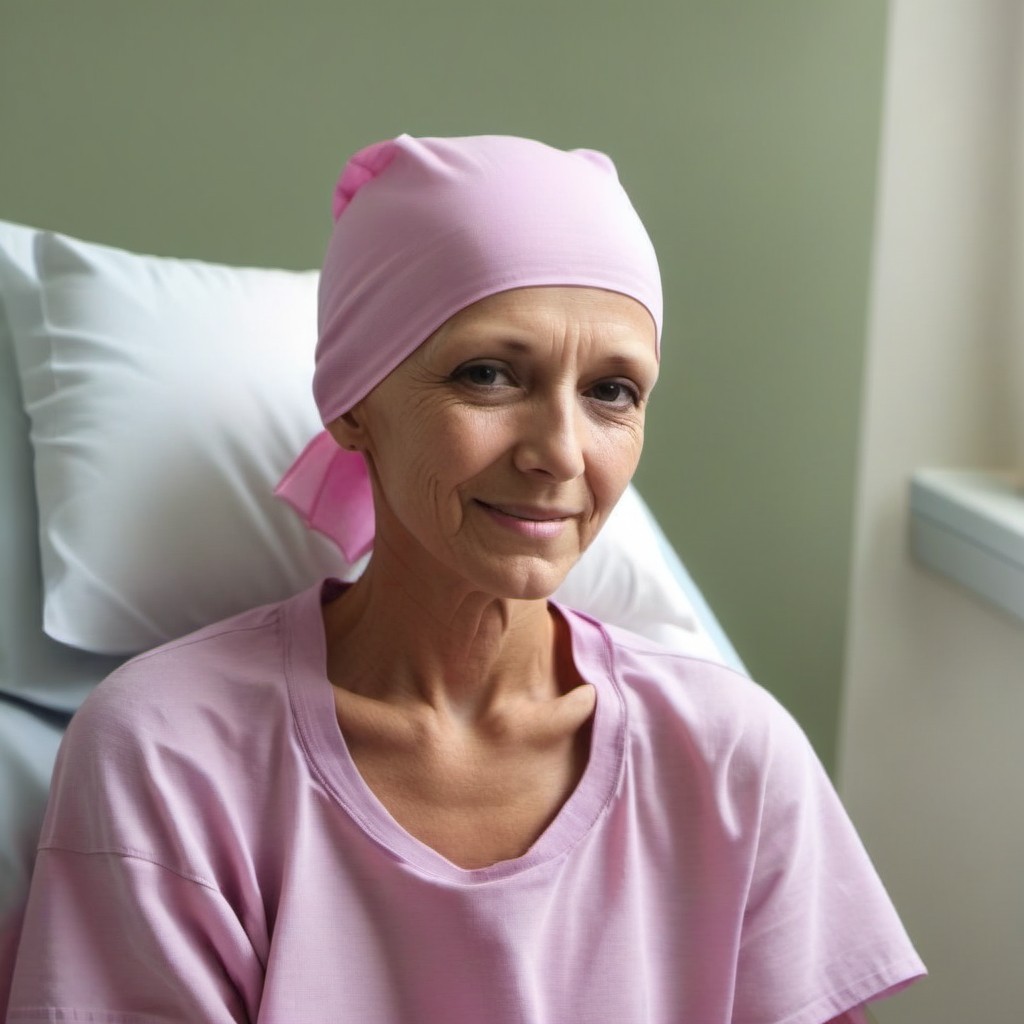

Supplement: Supplementary file 1 — Supplementary file1 (ZIP 11162 KB) [file 11764_2025_1760_MOESM1_ESM.zip › Data Images/cancer patient/Stable Diffusion/103.jpg]

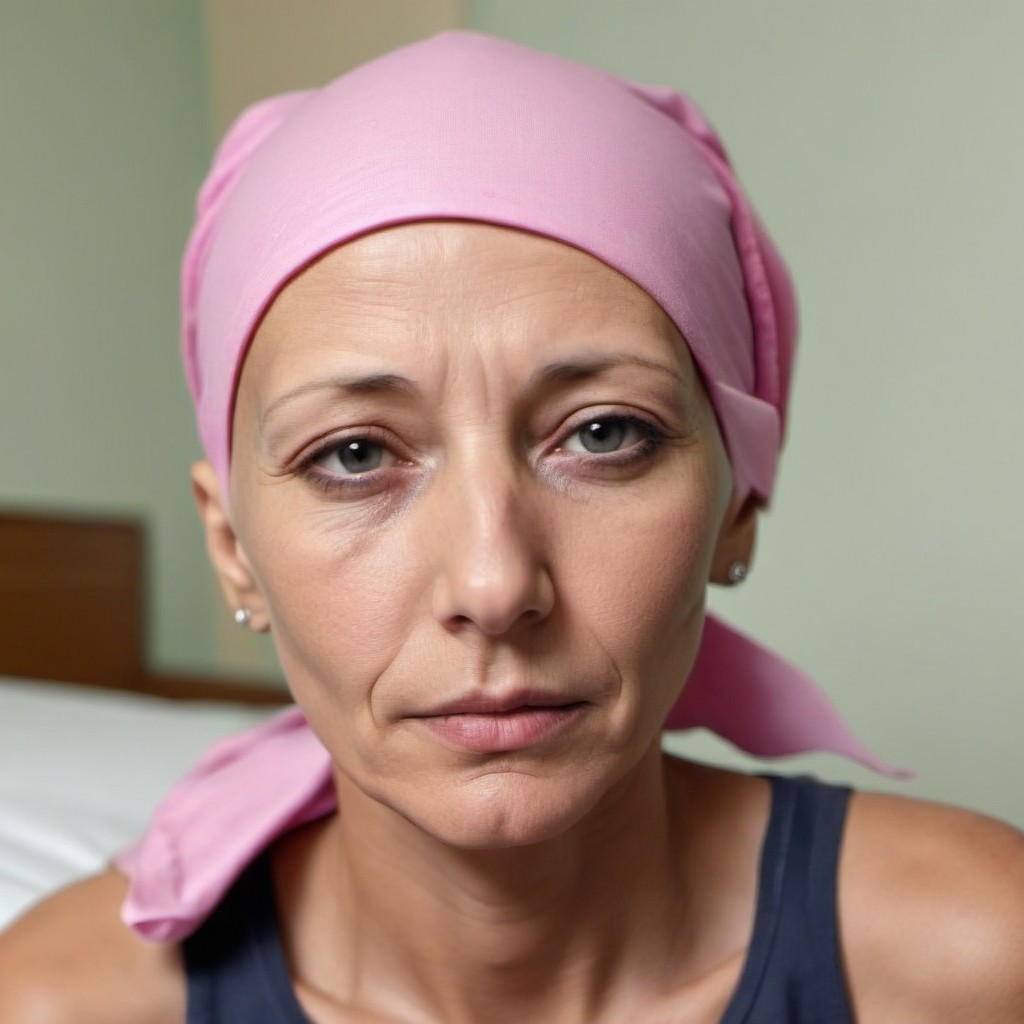

Supplement: Supplementary file 1 — Supplementary file1 (ZIP 11162 KB) [file 11764_2025_1760_MOESM1_ESM.zip › Data Images/cancer patient/Stable Diffusion/104.jpg]

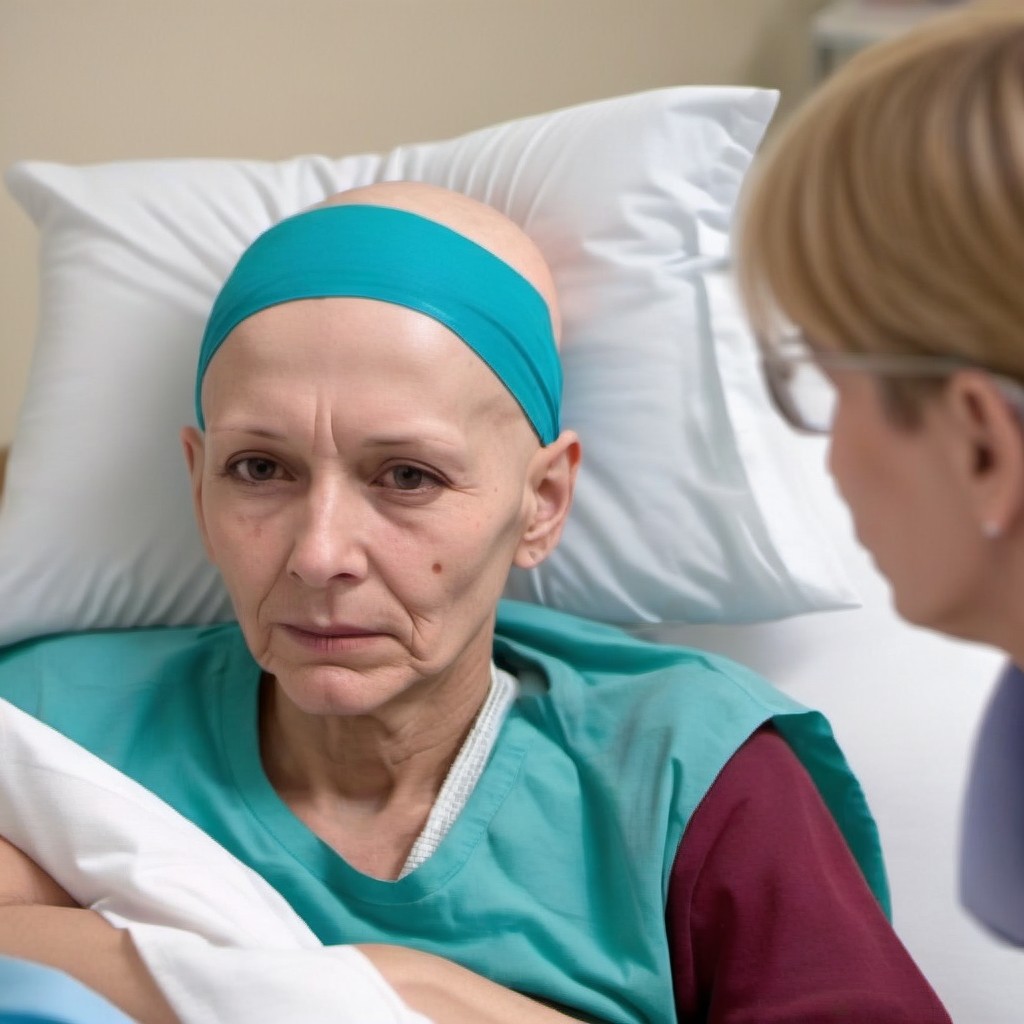

Supplement: Supplementary file 1 — Supplementary file1 (ZIP 11162 KB) [file 11764_2025_1760_MOESM1_ESM.zip › Data Images/cancer patient/Stable Diffusion/105.jpg]

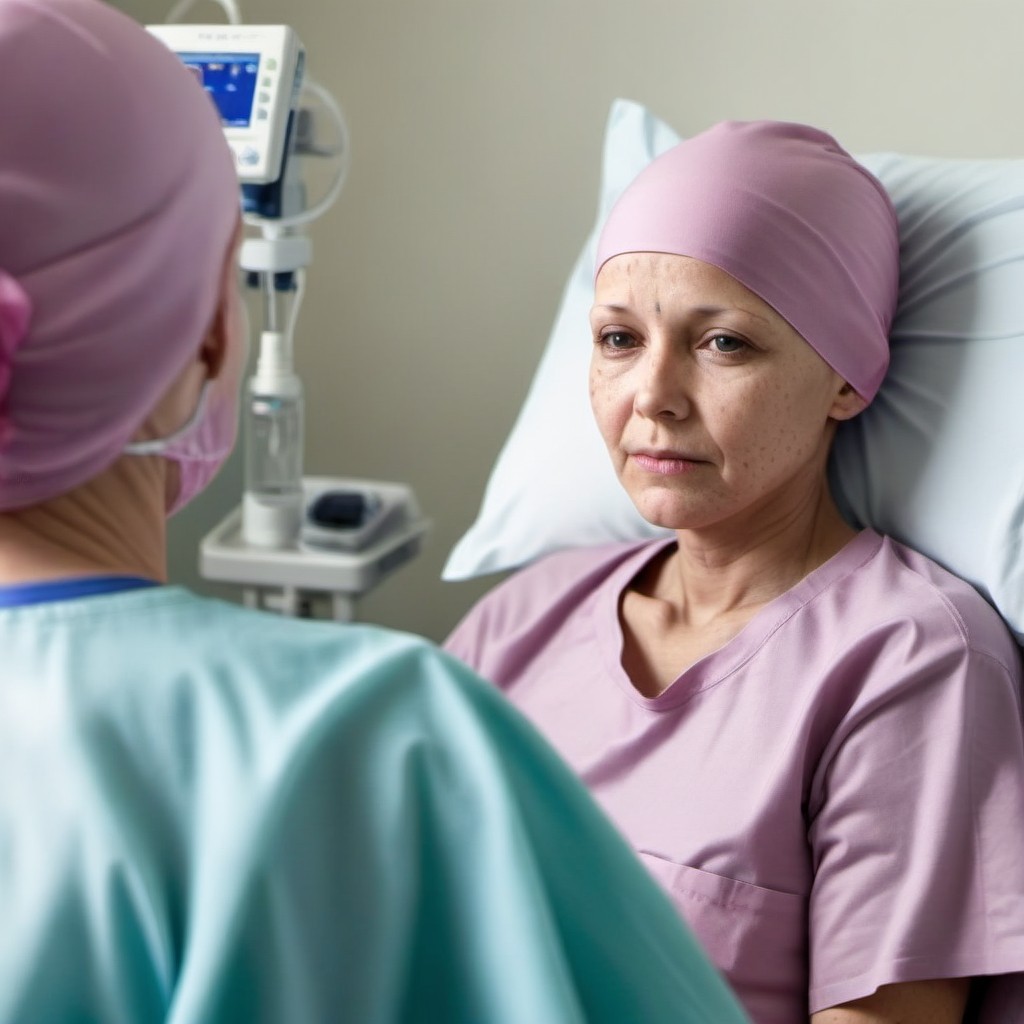

Supplement: Supplementary file 1 — Supplementary file1 (ZIP 11162 KB) [file 11764_2025_1760_MOESM1_ESM.zip › Data Images/cancer patient/Stable Diffusion/106.jpg]

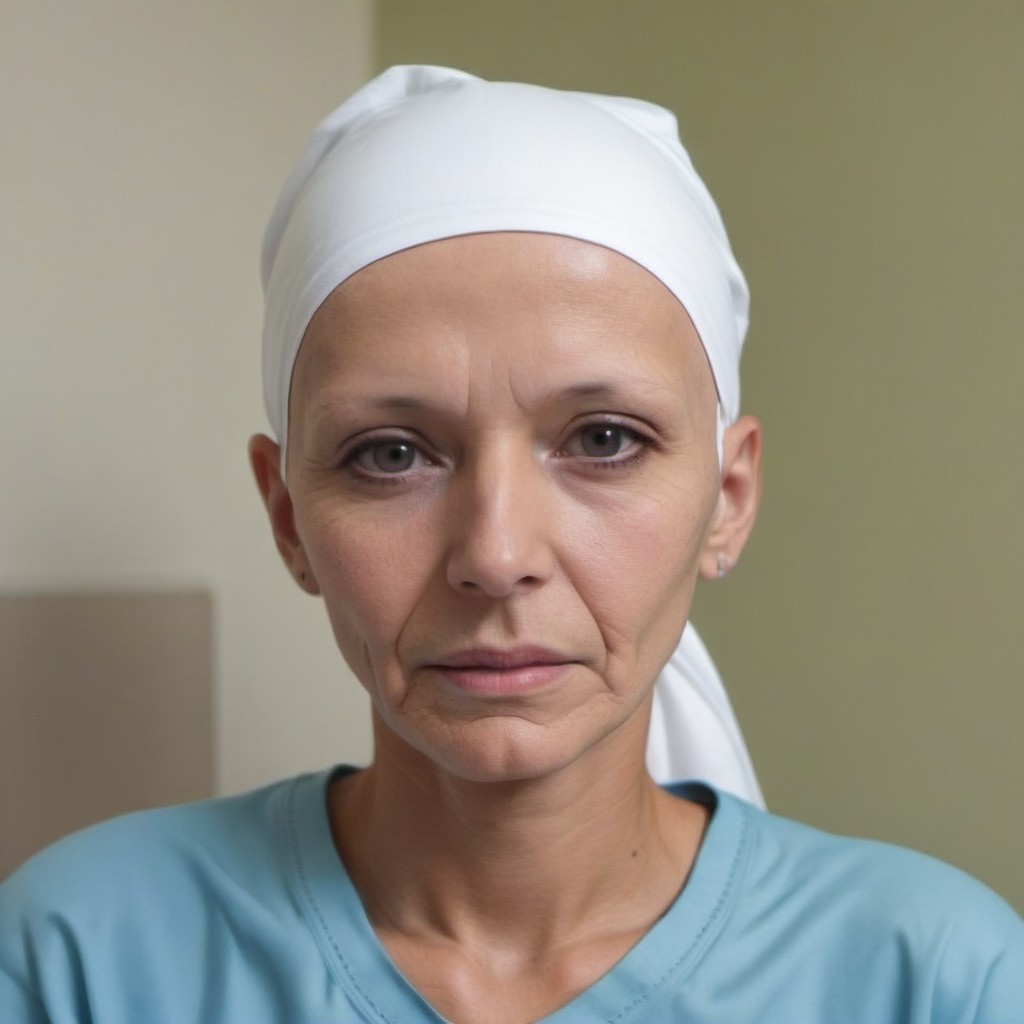

Supplement: Supplementary file 1 — Supplementary file1 (ZIP 11162 KB) [file 11764_2025_1760_MOESM1_ESM.zip › Data Images/cancer patient/Stable Diffusion/107.jpg]

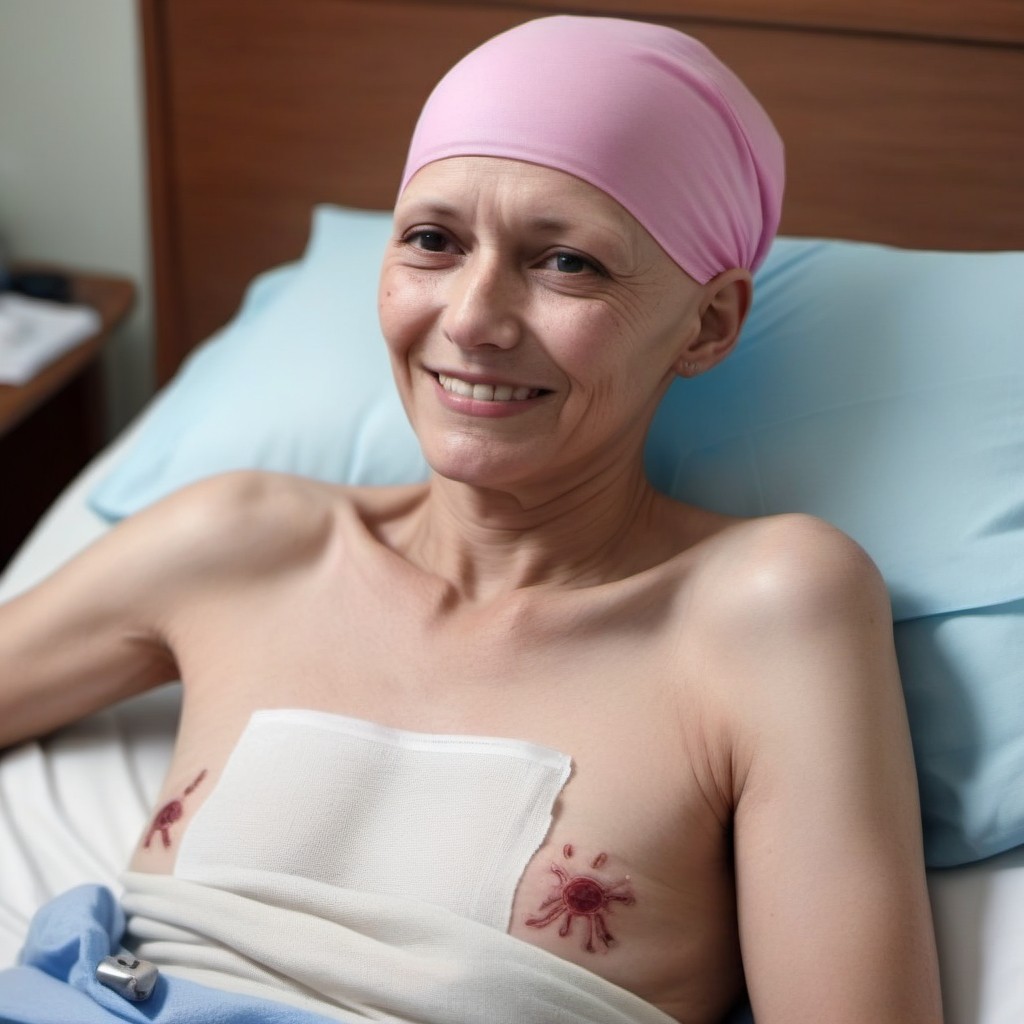

Supplement: Supplementary file 1 — Supplementary file1 (ZIP 11162 KB) [file 11764_2025_1760_MOESM1_ESM.zip › Data Images/cancer patient/Stable Diffusion/108.jpg]

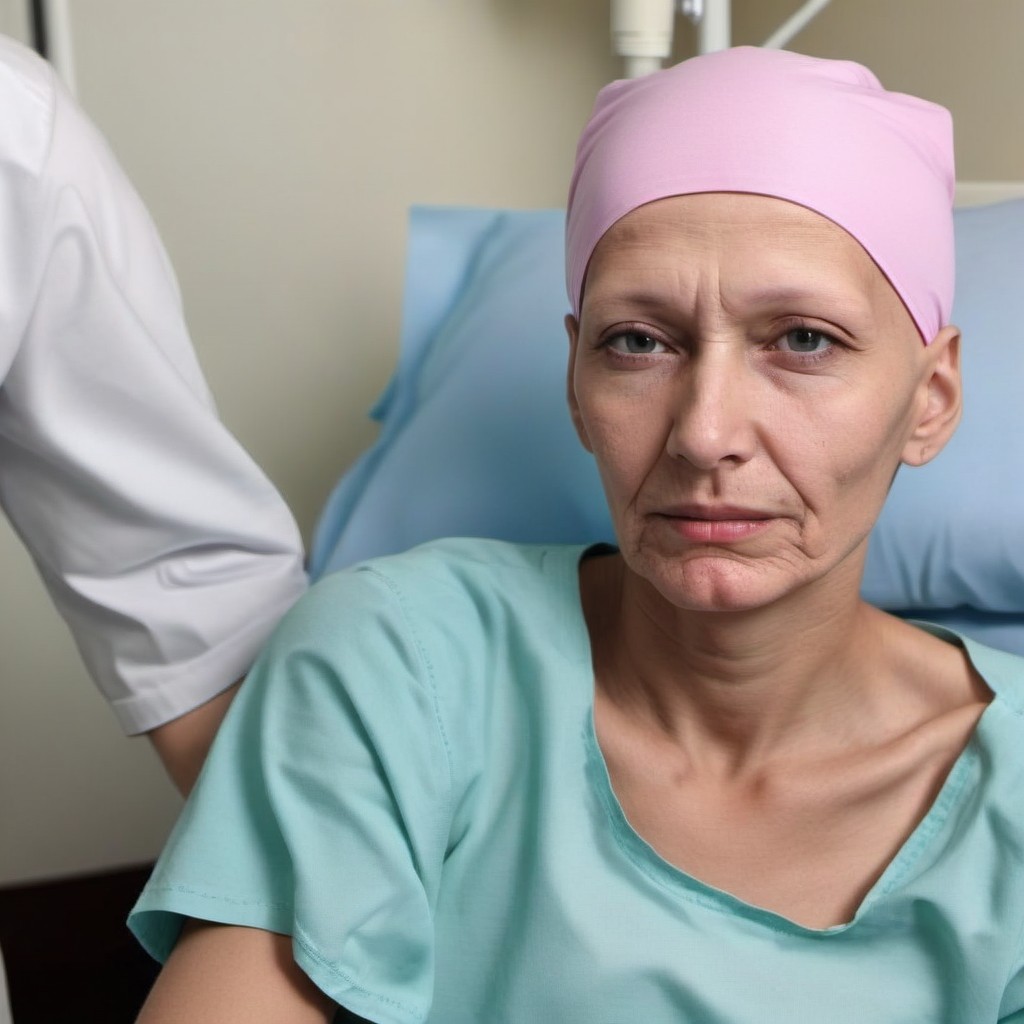

Supplement: Supplementary file 1 — Supplementary file1 (ZIP 11162 KB) [file 11764_2025_1760_MOESM1_ESM.zip › Data Images/cancer patient/Stable Diffusion/109.jpg]

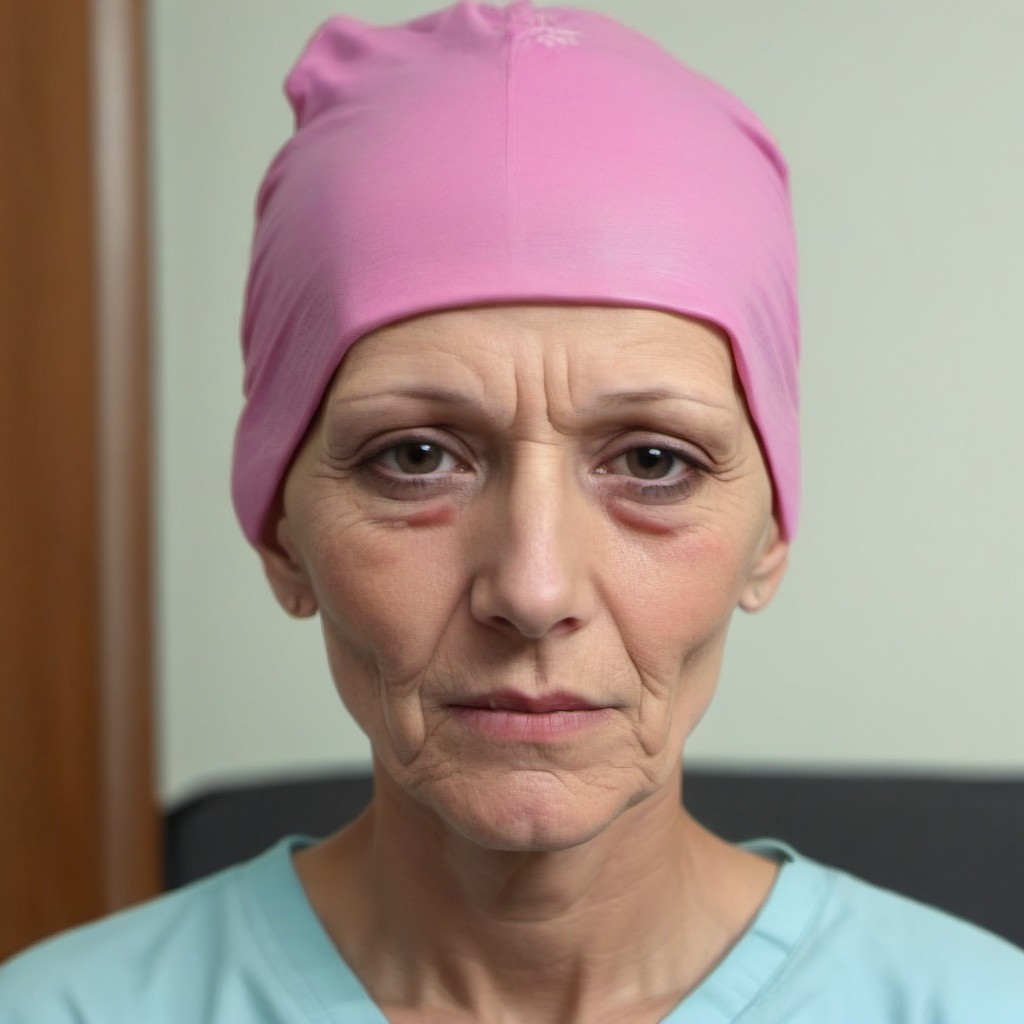

Supplement: Supplementary file 1 — Supplementary file1 (ZIP 11162 KB) [file 11764_2025_1760_MOESM1_ESM.zip › Data Images/cancer patient/Stable Diffusion/110.jpg]

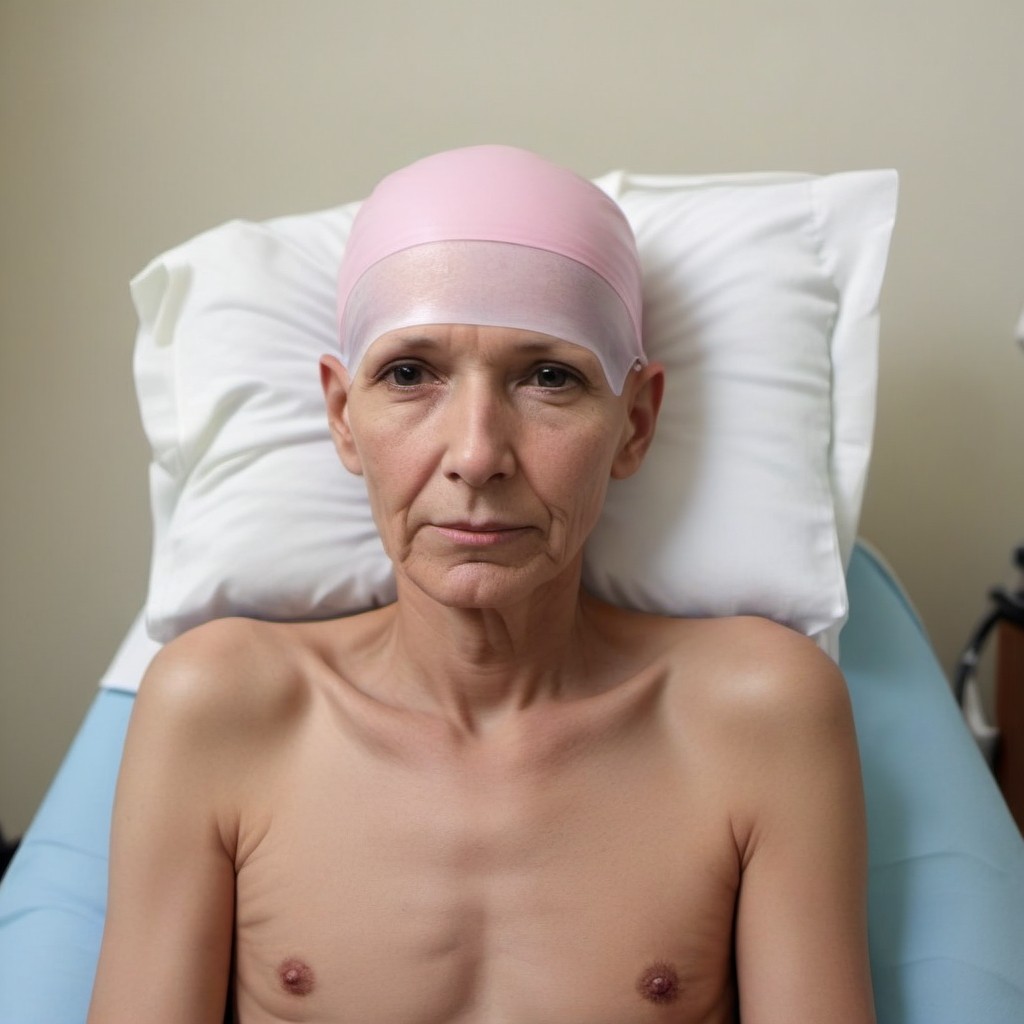

Supplement: Supplementary file 1 — Supplementary file1 (ZIP 11162 KB) [file 11764_2025_1760_MOESM1_ESM.zip › Data Images/cancer patient/Stable Diffusion/111.jpg]

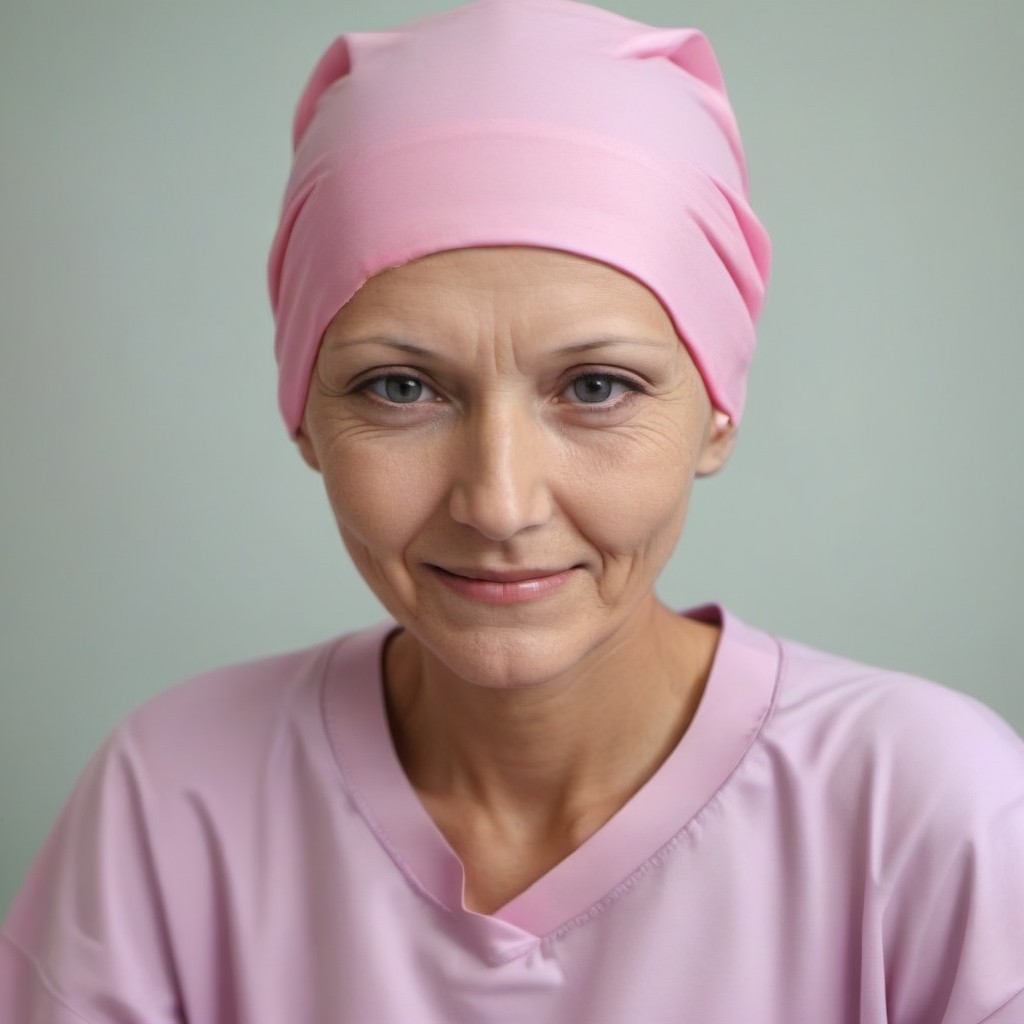

Supplement: Supplementary file 1 — Supplementary file1 (ZIP 11162 KB) [file 11764_2025_1760_MOESM1_ESM.zip › Data Images/cancer patient/Stable Diffusion/112.jpg]

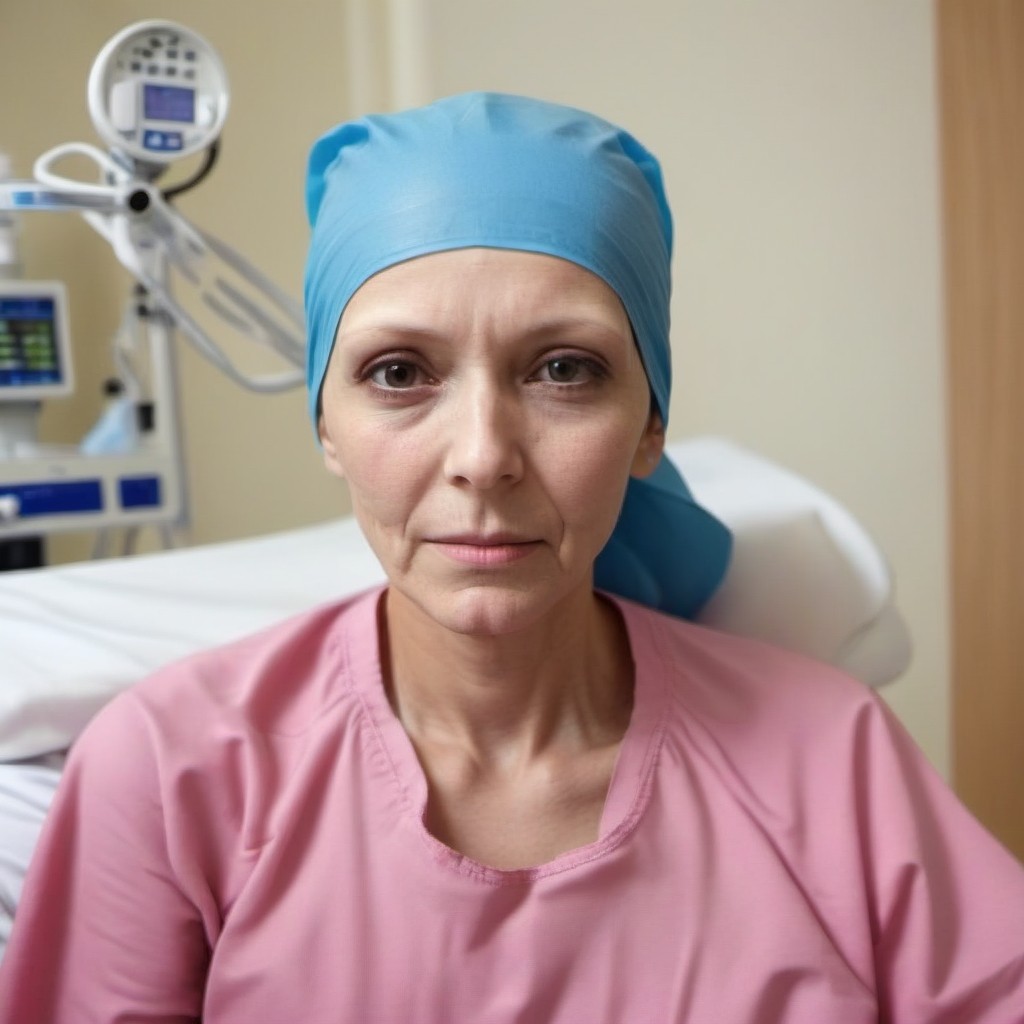

Supplement: Supplementary file 1 — Supplementary file1 (ZIP 11162 KB) [file 11764_2025_1760_MOESM1_ESM.zip › Data Images/cancer patient/Stable Diffusion/113.jpg]

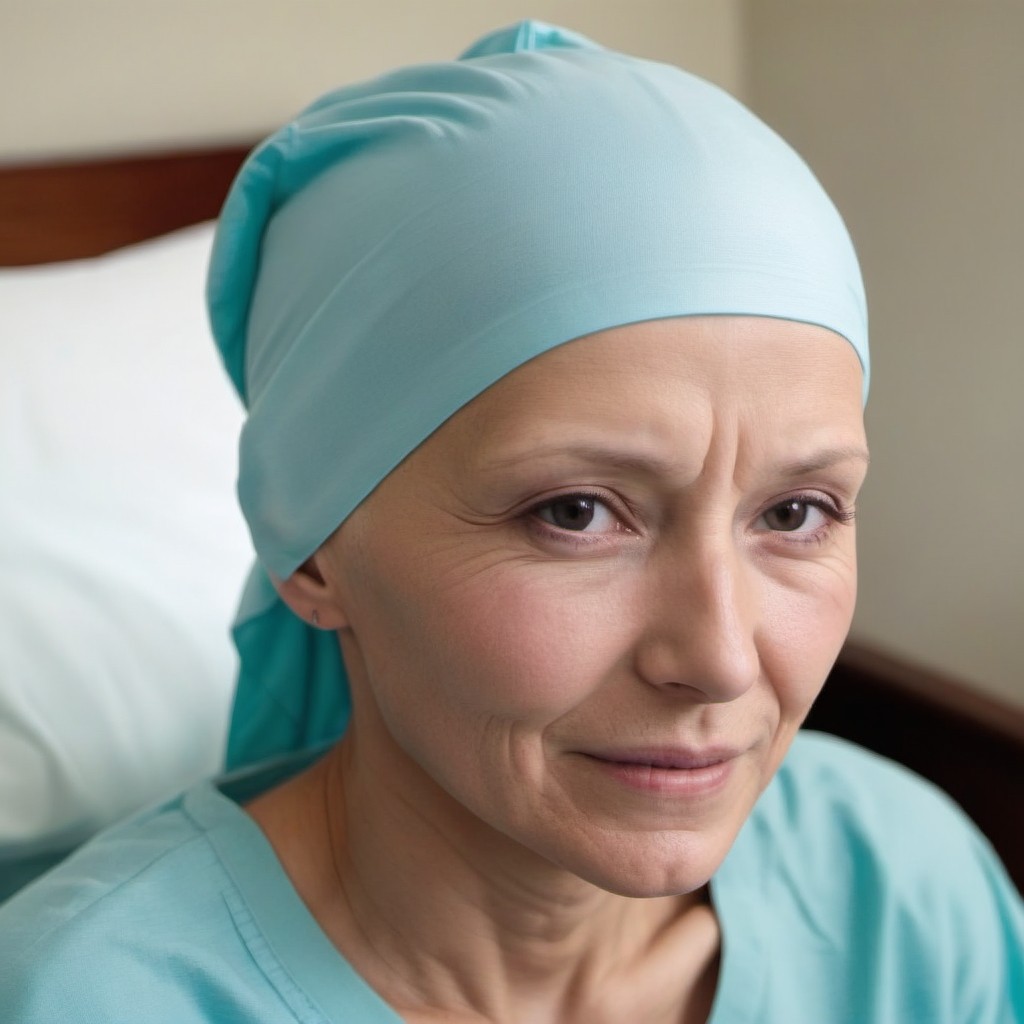

Supplement: Supplementary file 1 — Supplementary file1 (ZIP 11162 KB) [file 11764_2025_1760_MOESM1_ESM.zip › Data Images/cancer patient/Stable Diffusion/114.jpg]

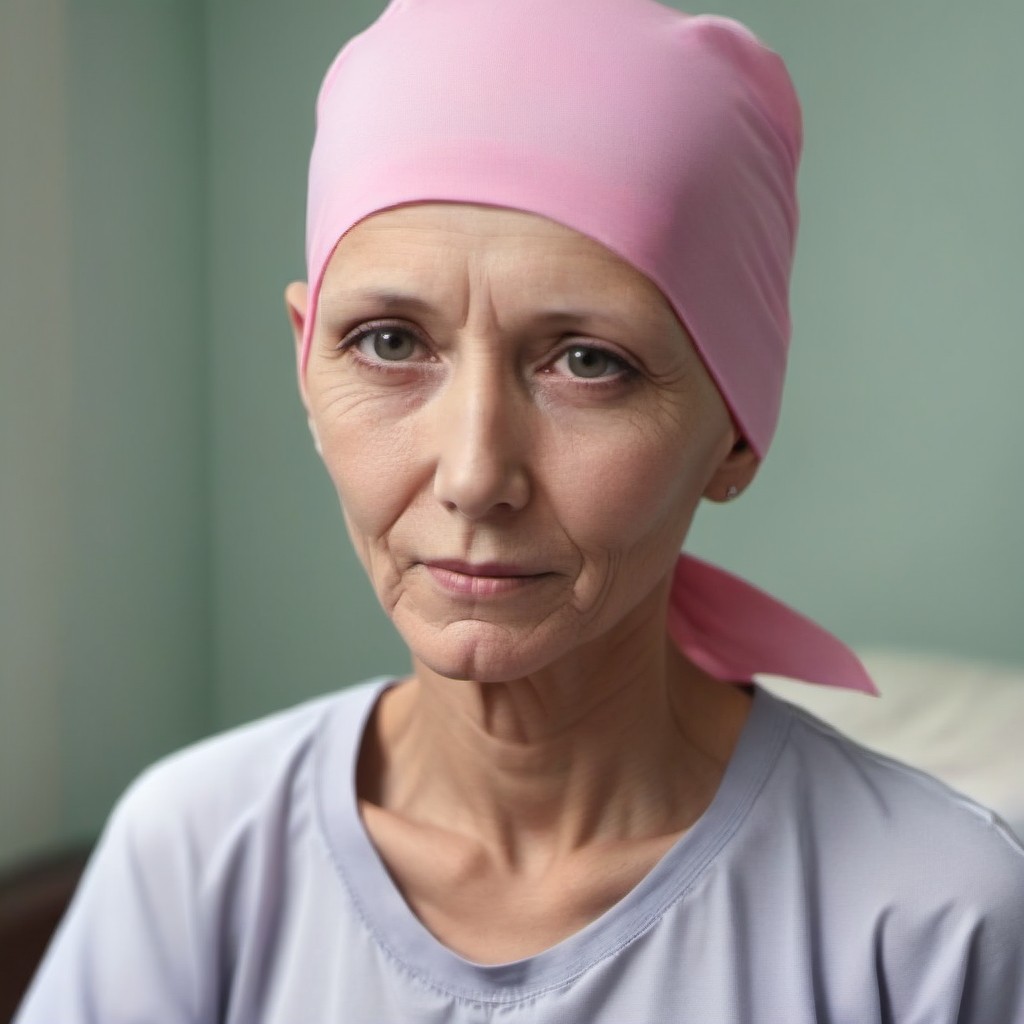

Supplement: Supplementary file 1 — Supplementary file1 (ZIP 11162 KB) [file 11764_2025_1760_MOESM1_ESM.zip › Data Images/cancer patient/Stable Diffusion/115.jpg]

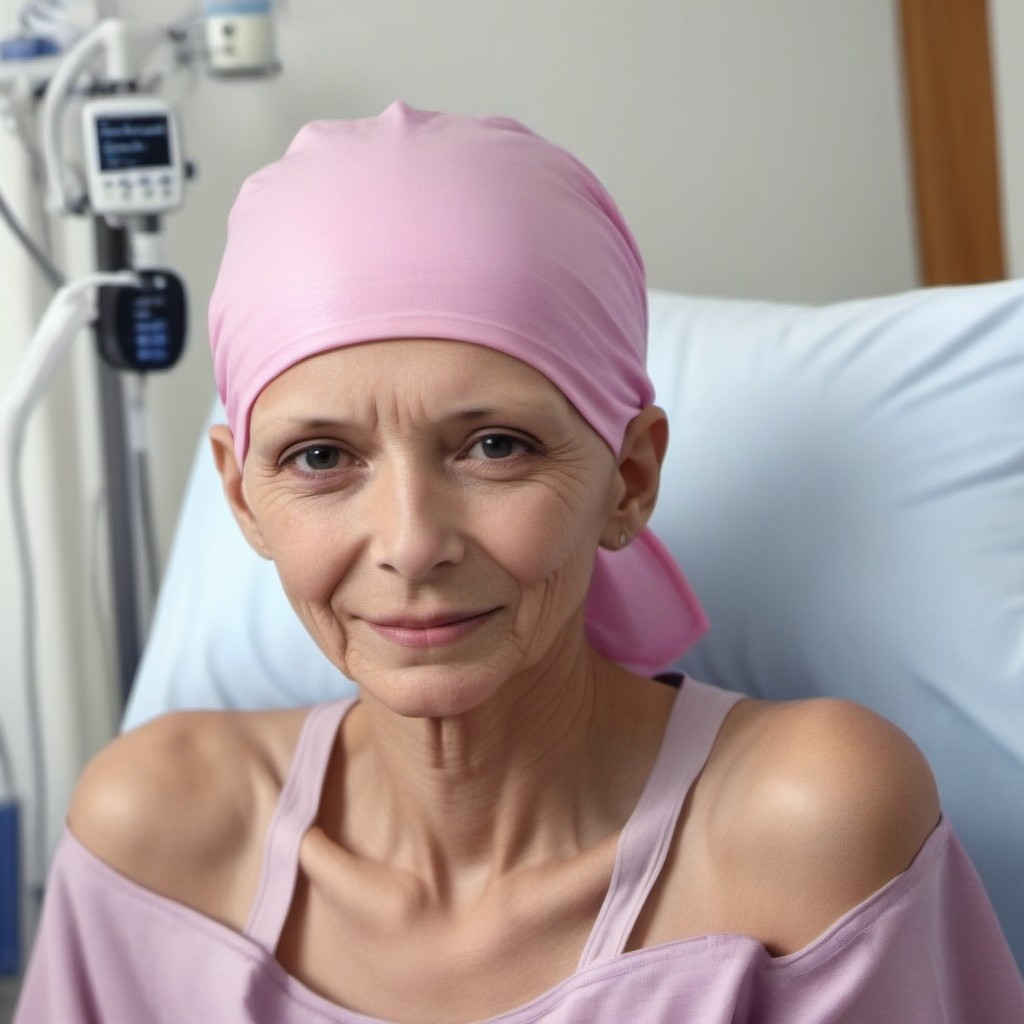

Supplement: Supplementary file 1 — Supplementary file1 (ZIP 11162 KB) [file 11764_2025_1760_MOESM1_ESM.zip › Data Images/cancer patient/Stable Diffusion/116.jpg]

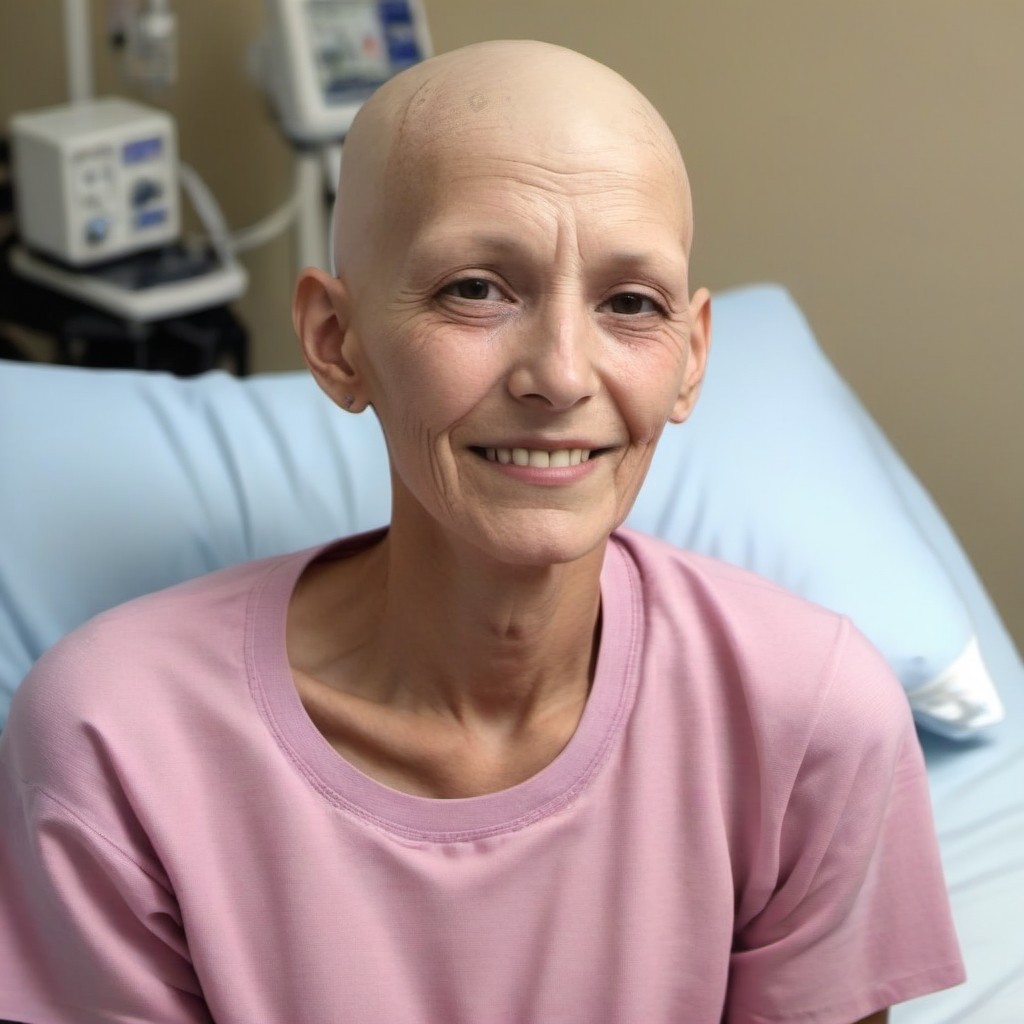

Supplement: Supplementary file 1 — Supplementary file1 (ZIP 11162 KB) [file 11764_2025_1760_MOESM1_ESM.zip › Data Images/cancer patient/Stable Diffusion/117.jpg]

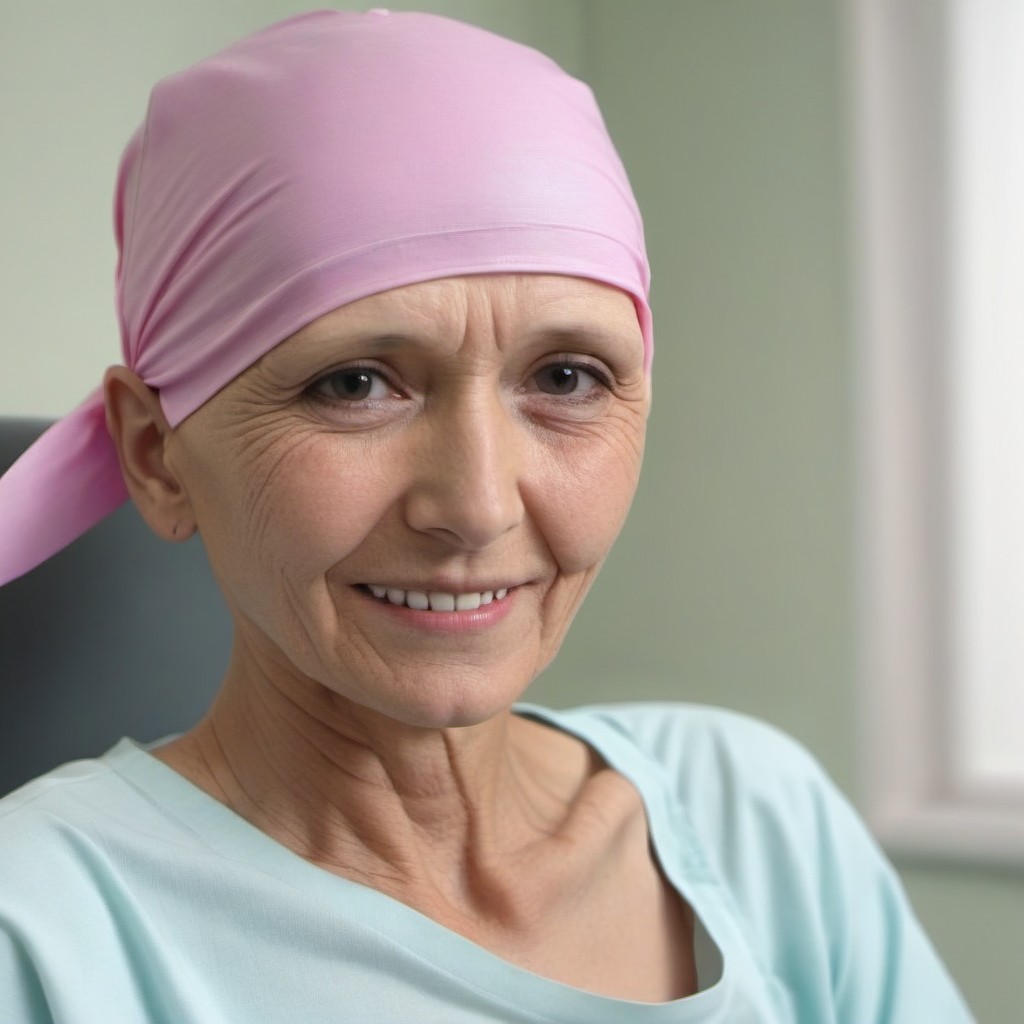

Supplement: Supplementary file 1 — Supplementary file1 (ZIP 11162 KB) [file 11764_2025_1760_MOESM1_ESM.zip › Data Images/cancer patient/Stable Diffusion/118.jpg]

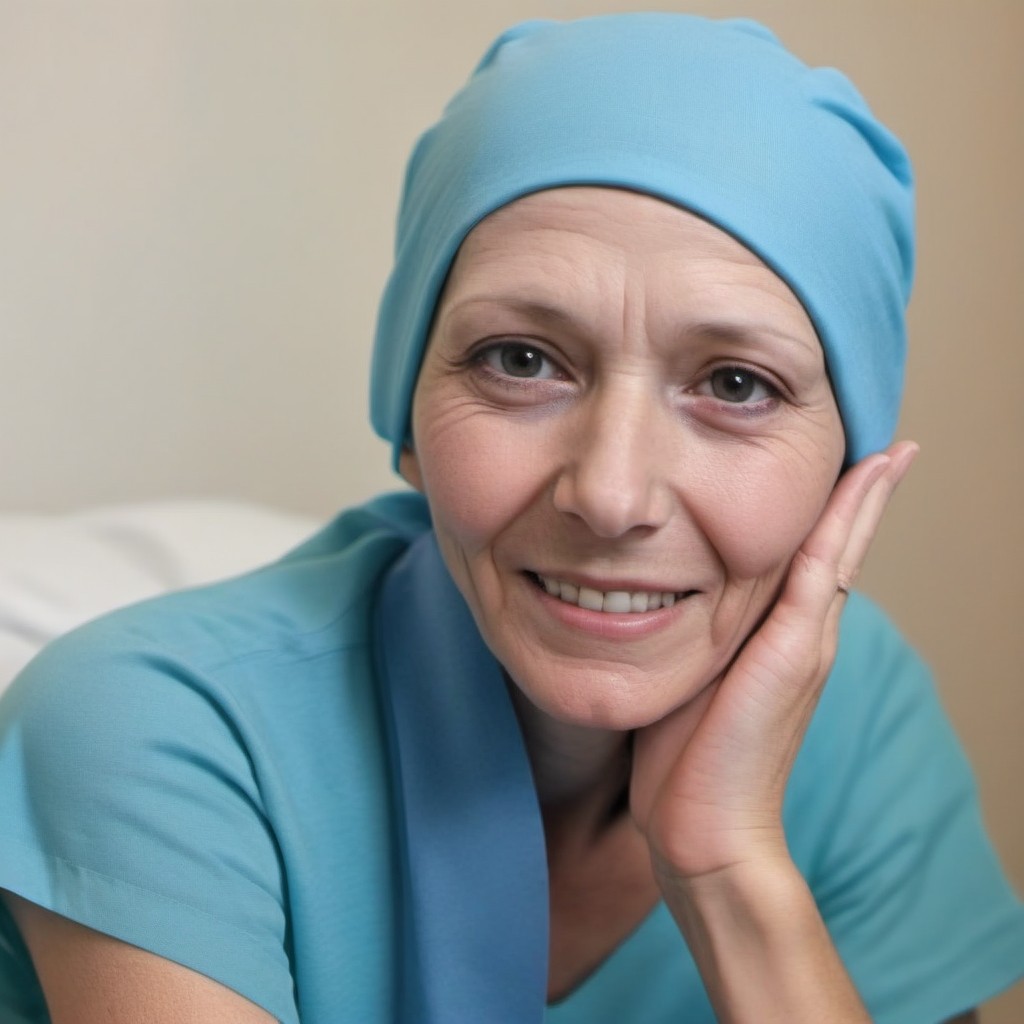

Supplement: Supplementary file 1 — Supplementary file1 (ZIP 11162 KB) [file 11764_2025_1760_MOESM1_ESM.zip › Data Images/cancer patient/Stable Diffusion/119.jpg]

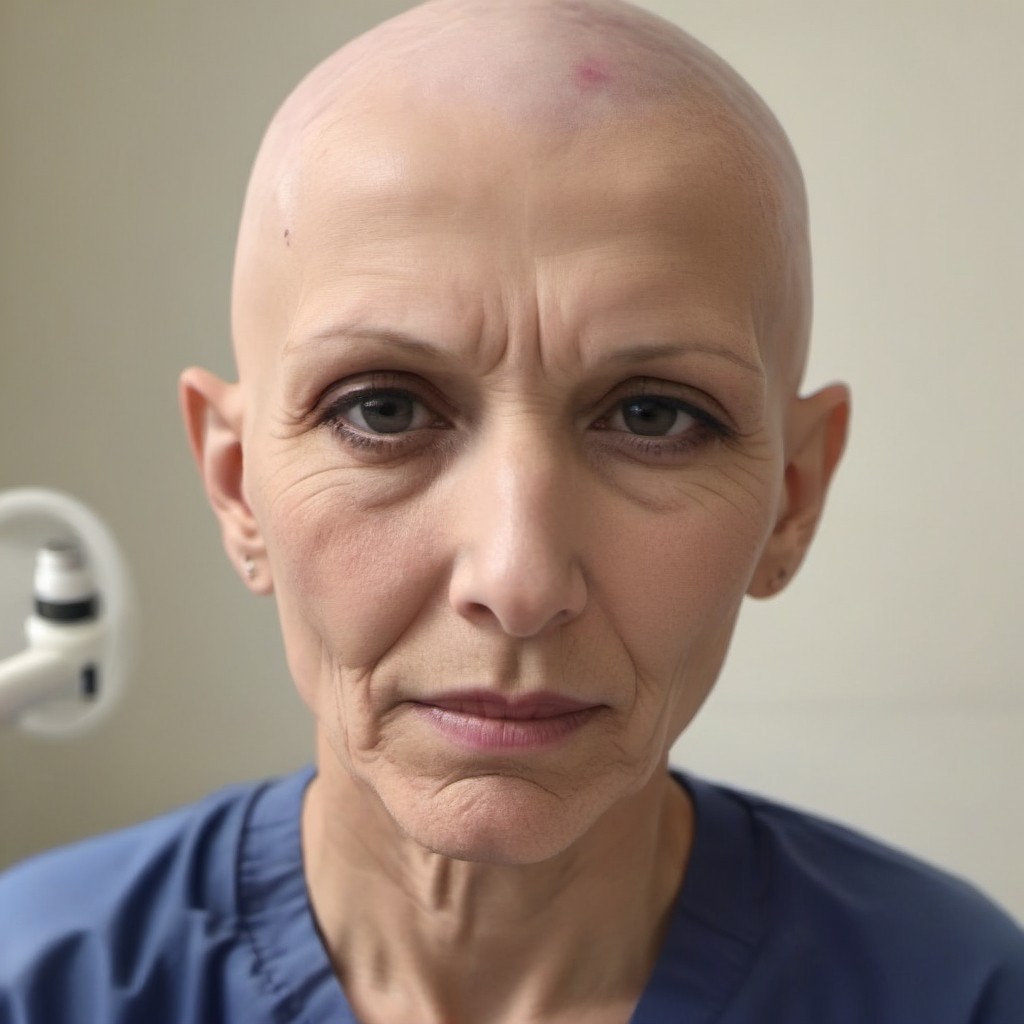

Supplement: Supplementary file 1 — Supplementary file1 (ZIP 11162 KB) [file 11764_2025_1760_MOESM1_ESM.zip › Data Images/cancer patient/Stable Diffusion/120.jpg]

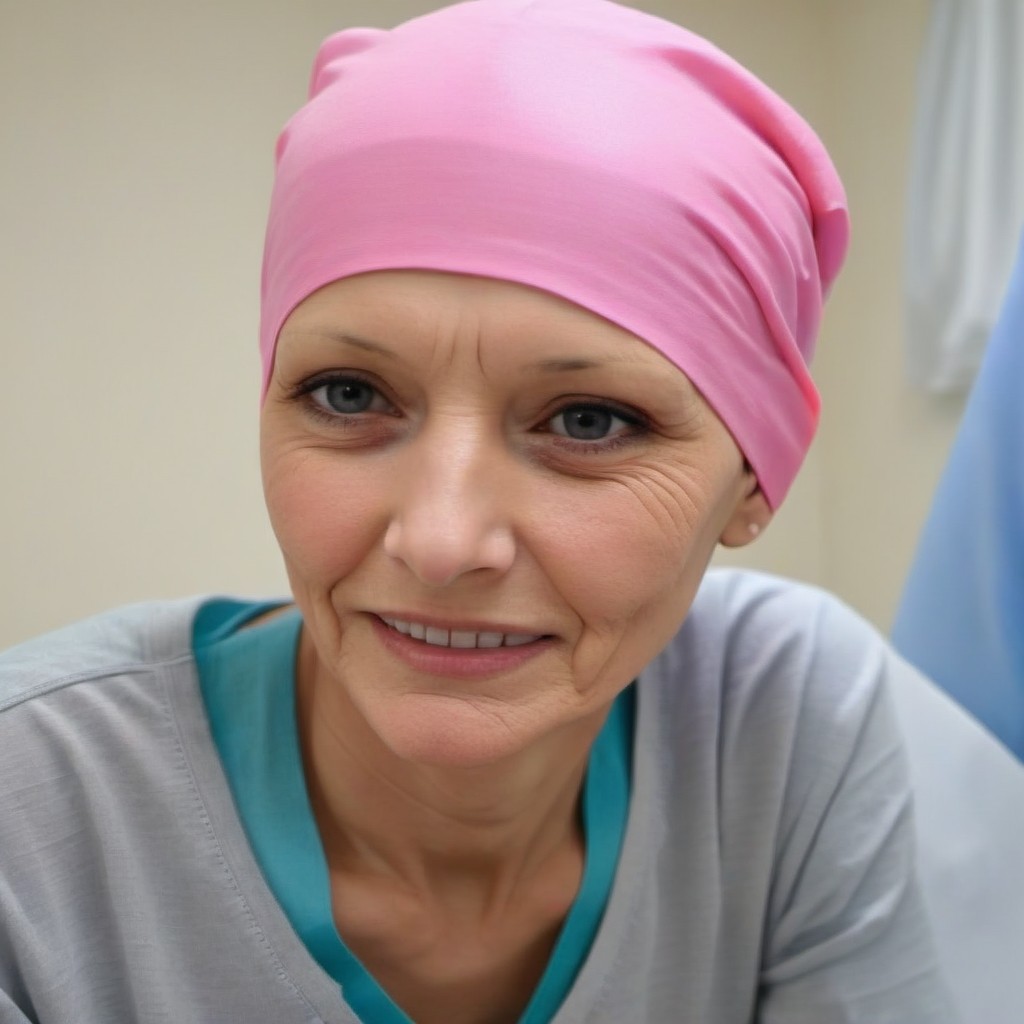

Supplement: Supplementary file 1 — Supplementary file1 (ZIP 11162 KB) [file 11764_2025_1760_MOESM1_ESM.zip › Data Images/cancer patient/Stable Diffusion/121.jpg]

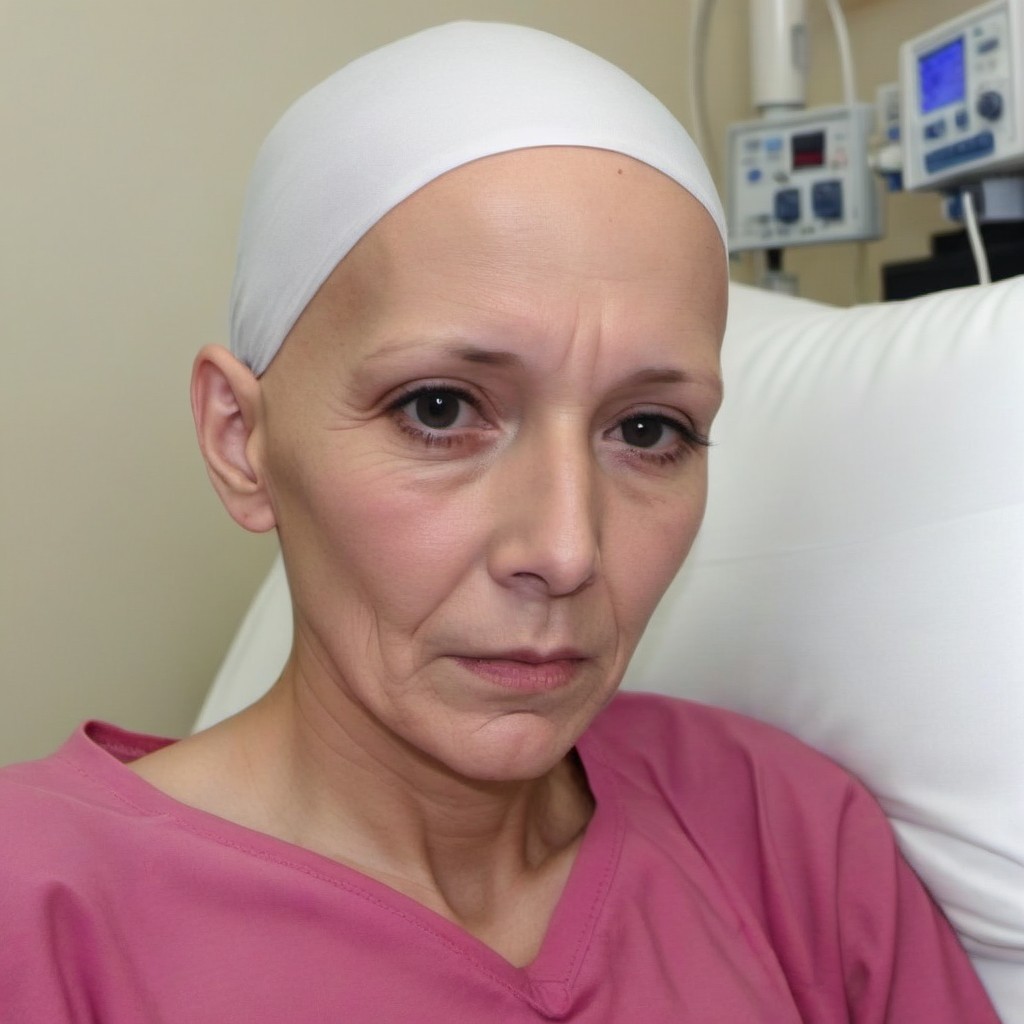

Supplement: Supplementary file 1 — Supplementary file1 (ZIP 11162 KB) [file 11764_2025_1760_MOESM1_ESM.zip › Data Images/cancer patient/Stable Diffusion/122.jpg]

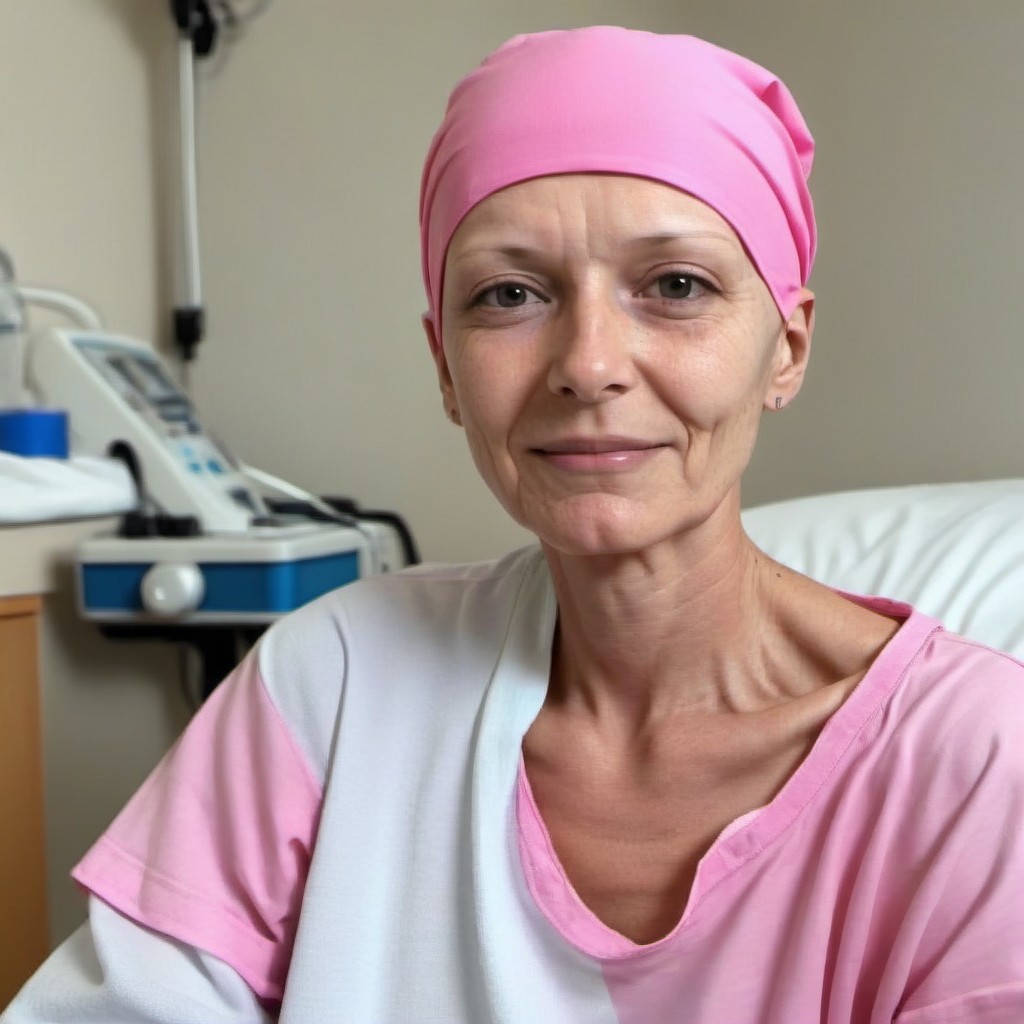

Supplement: Supplementary file 1 — Supplementary file1 (ZIP 11162 KB) [file 11764_2025_1760_MOESM1_ESM.zip › Data Images/cancer patient/Stable Diffusion/123.jpg]

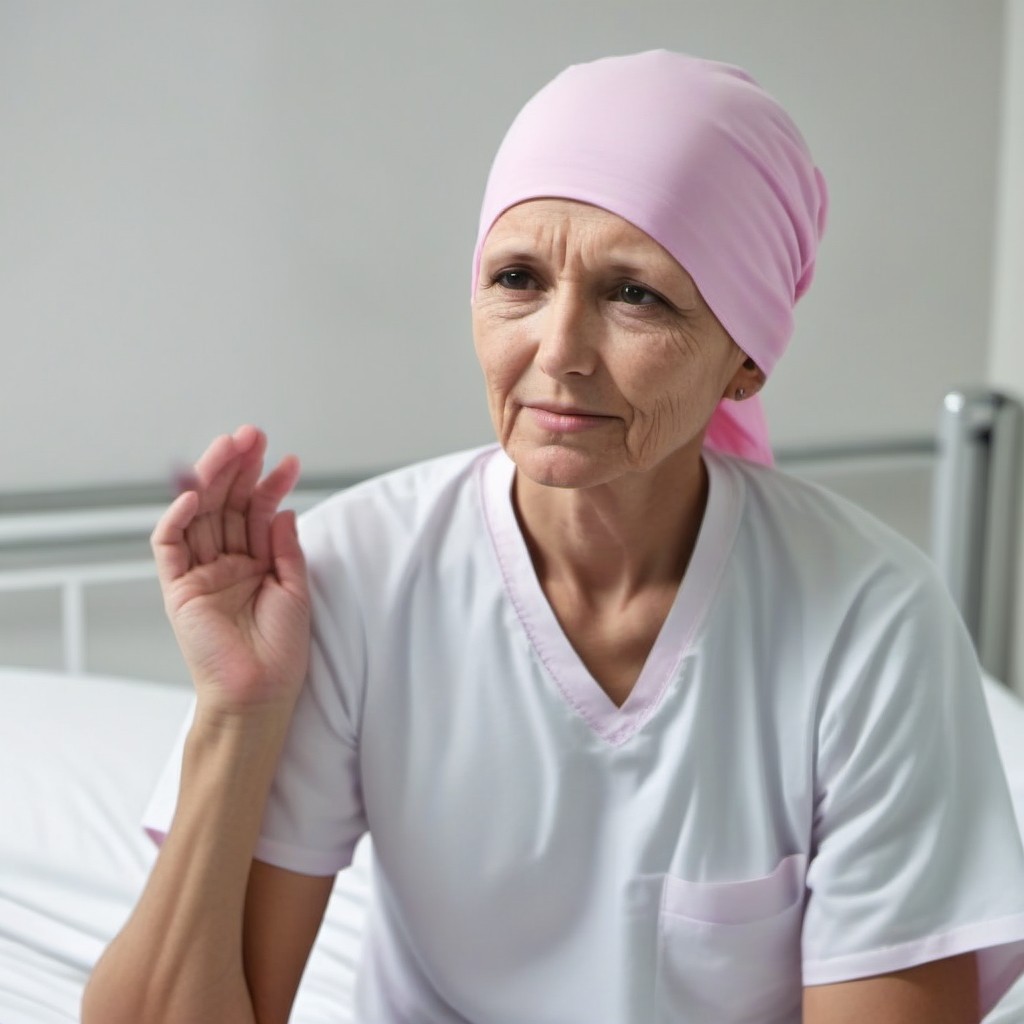

Supplement: Supplementary file 1 — Supplementary file1 (ZIP 11162 KB) [file 11764_2025_1760_MOESM1_ESM.zip › Data Images/cancer patient/Stable Diffusion/124.jpg]

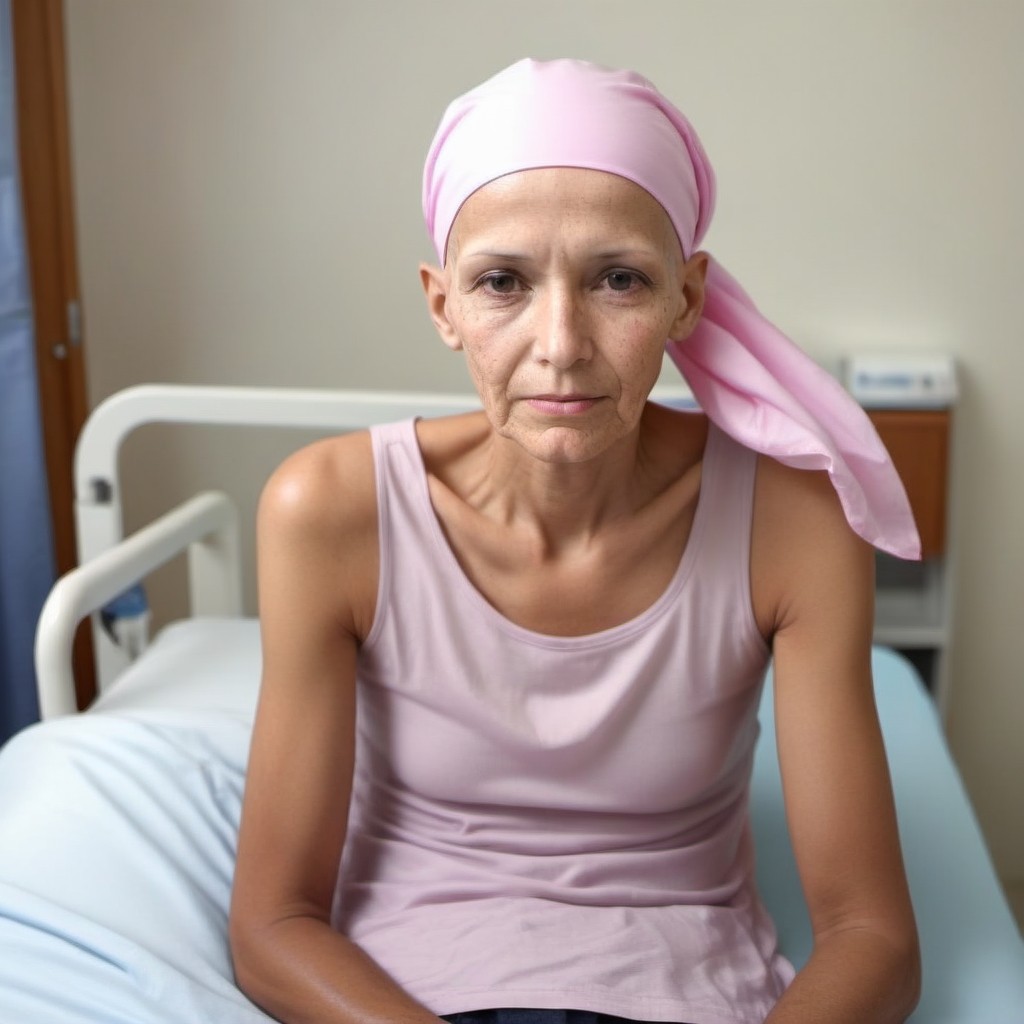

Supplement: Supplementary file 1 — Supplementary file1 (ZIP 11162 KB) [file 11764_2025_1760_MOESM1_ESM.zip › Data Images/cancer patient/Stable Diffusion/125.jpg]

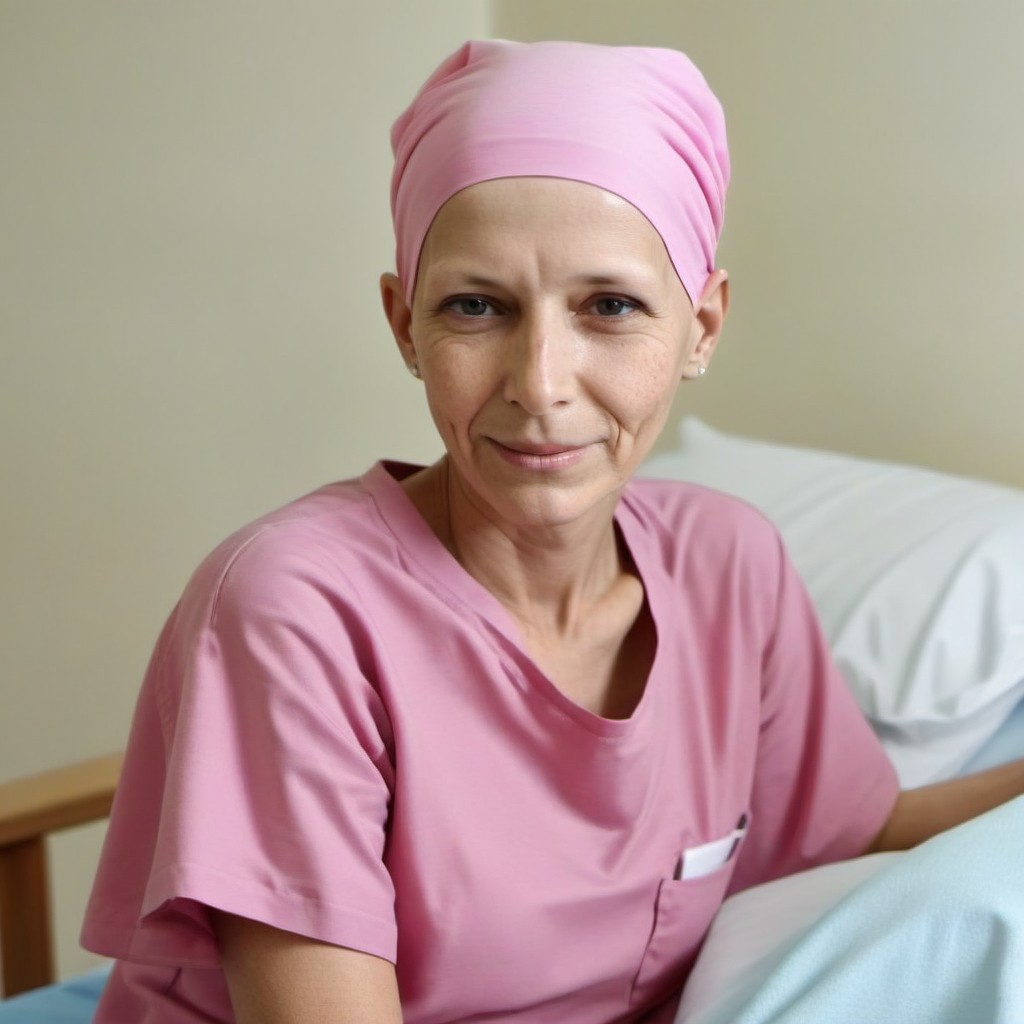

Supplement: Supplementary file 1 — Supplementary file1 (ZIP 11162 KB) [file 11764_2025_1760_MOESM1_ESM.zip › Data Images/cancer patient/Stable Diffusion/126.jpg]

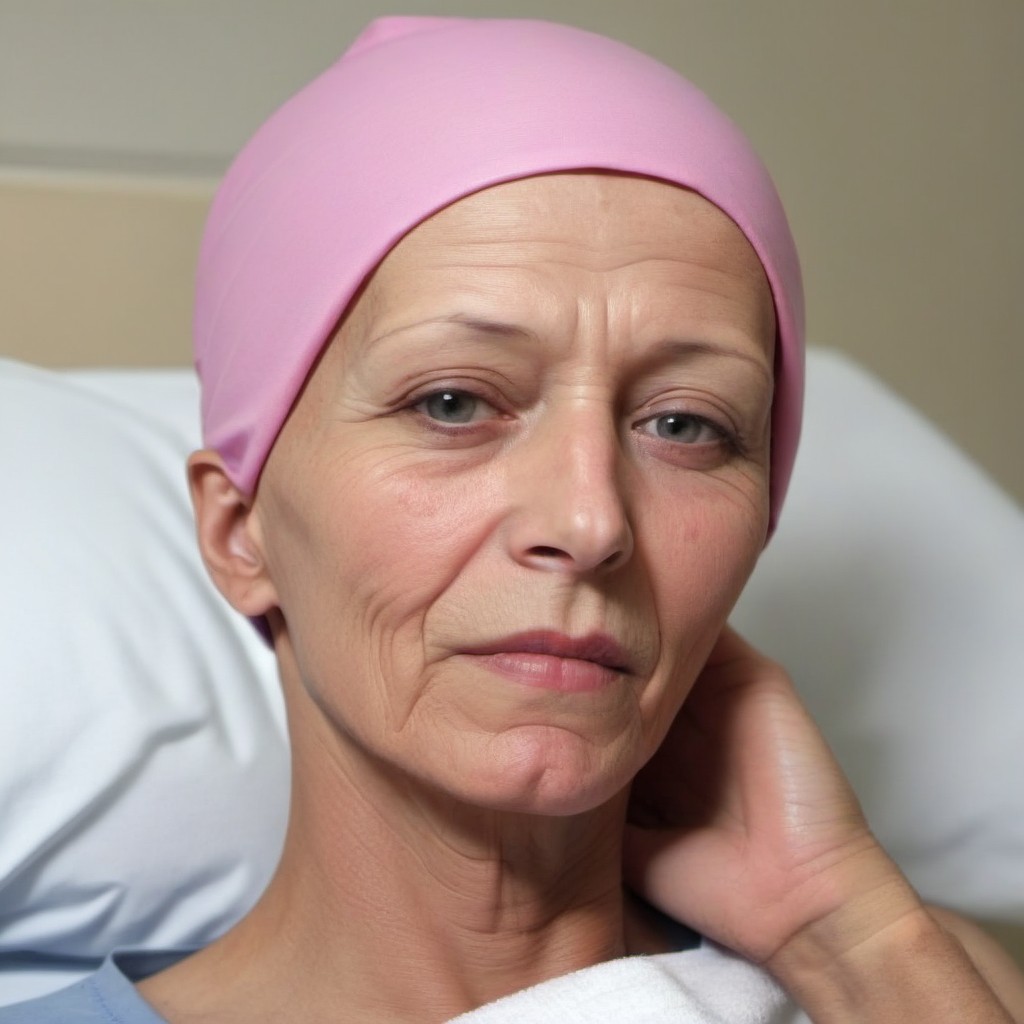

Supplement: Supplementary file 1 — Supplementary file1 (ZIP 11162 KB) [file 11764_2025_1760_MOESM1_ESM.zip › Data Images/cancer patient/Stable Diffusion/127.jpg]

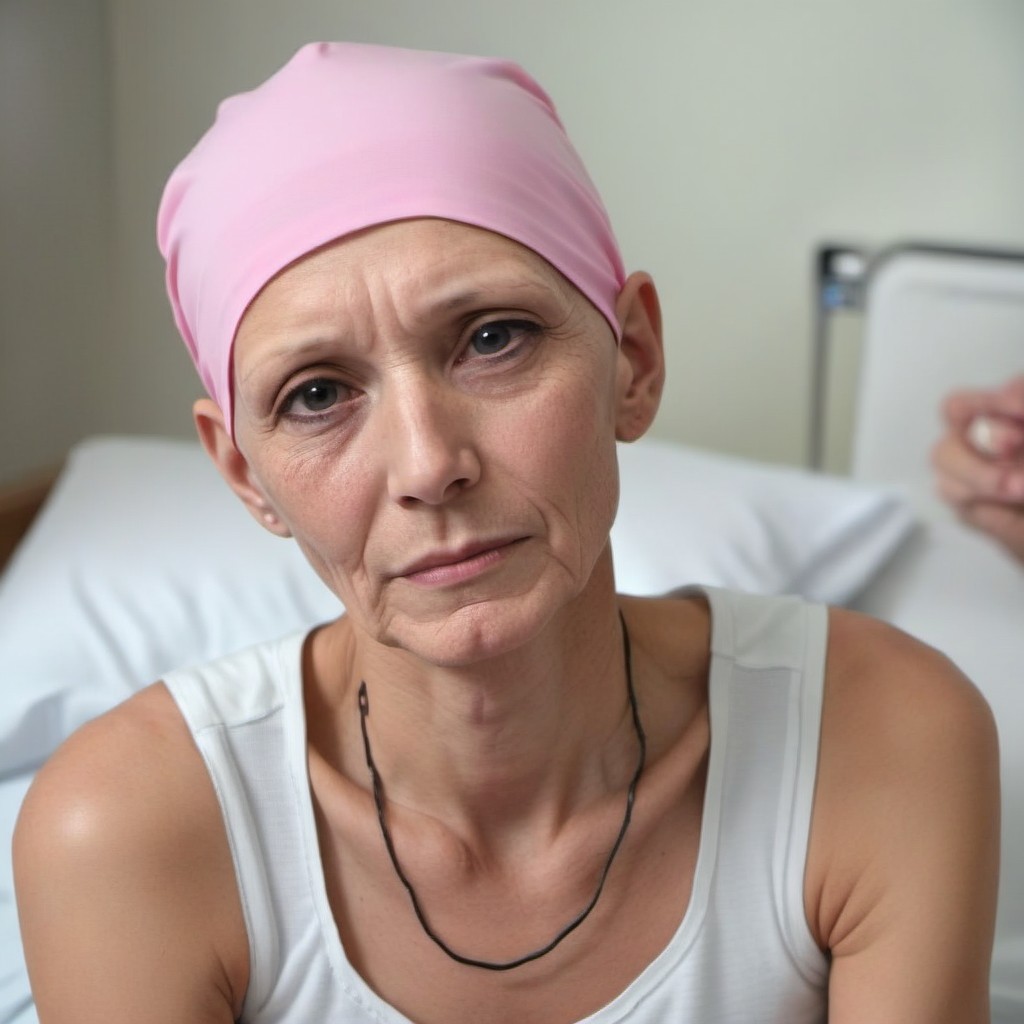

Supplement: Supplementary file 1 — Supplementary file1 (ZIP 11162 KB) [file 11764_2025_1760_MOESM1_ESM.zip › Data Images/cancer patient/Stable Diffusion/128.jpg]

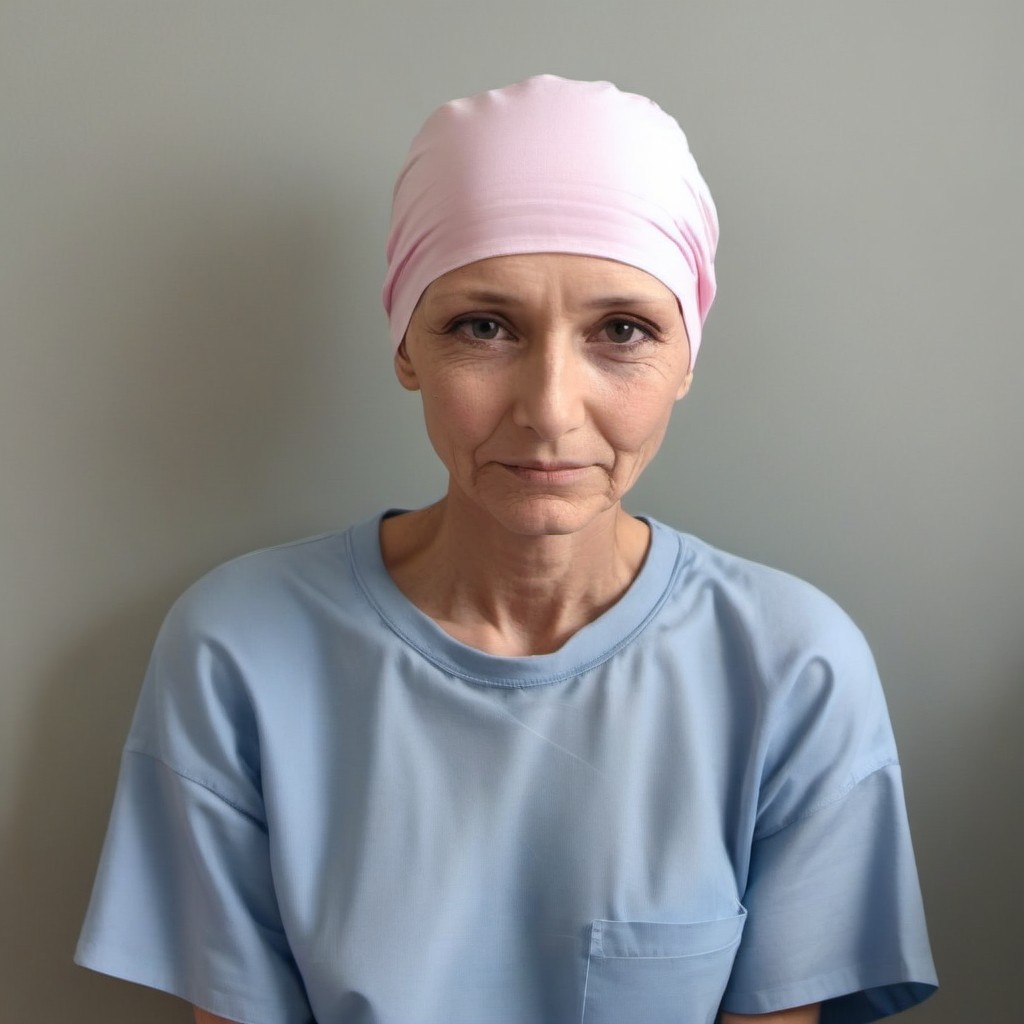

Supplement: Supplementary file 1 — Supplementary file1 (ZIP 11162 KB) [file 11764_2025_1760_MOESM1_ESM.zip › Data Images/cancer patient/Stable Diffusion/129.jpg]

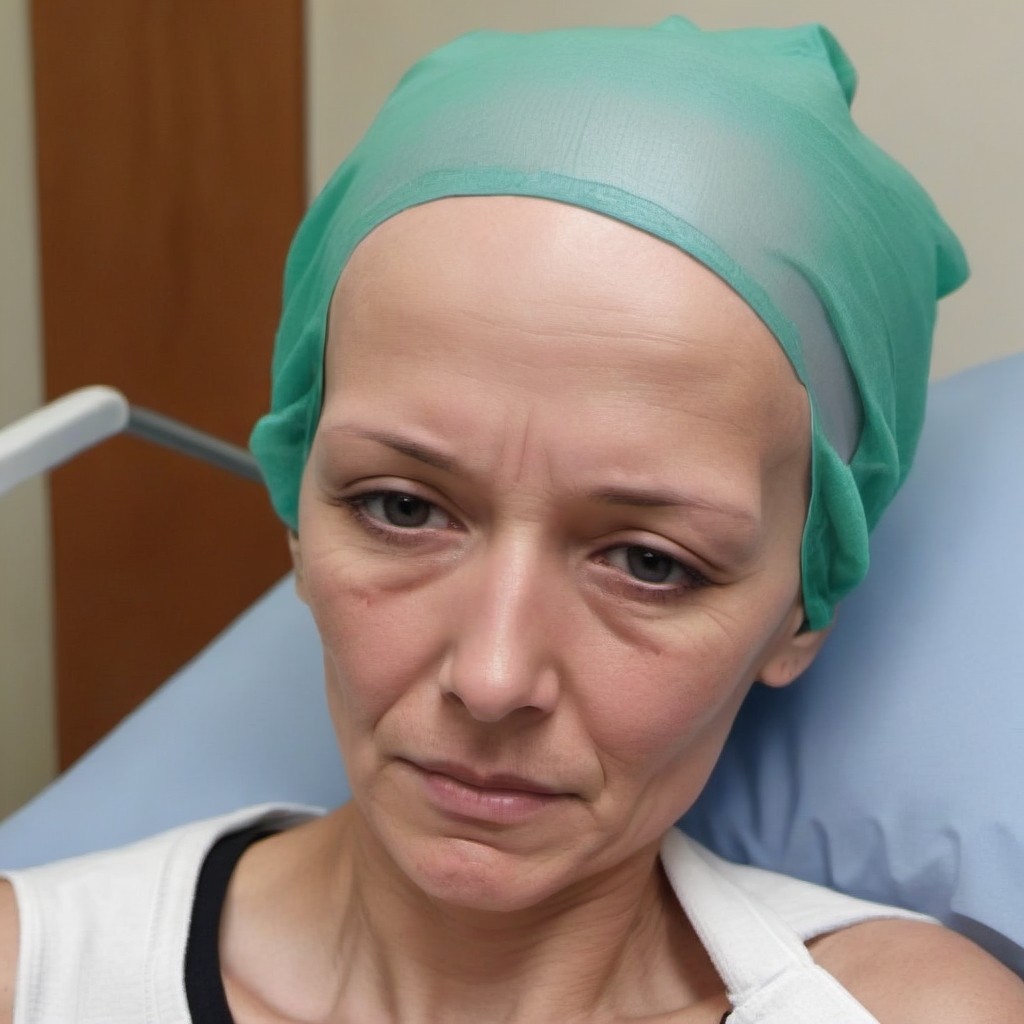

Supplement: Supplementary file 1 — Supplementary file1 (ZIP 11162 KB) [file 11764_2025_1760_MOESM1_ESM.zip › Data Images/cancer patient/Stable Diffusion/130.jpg]

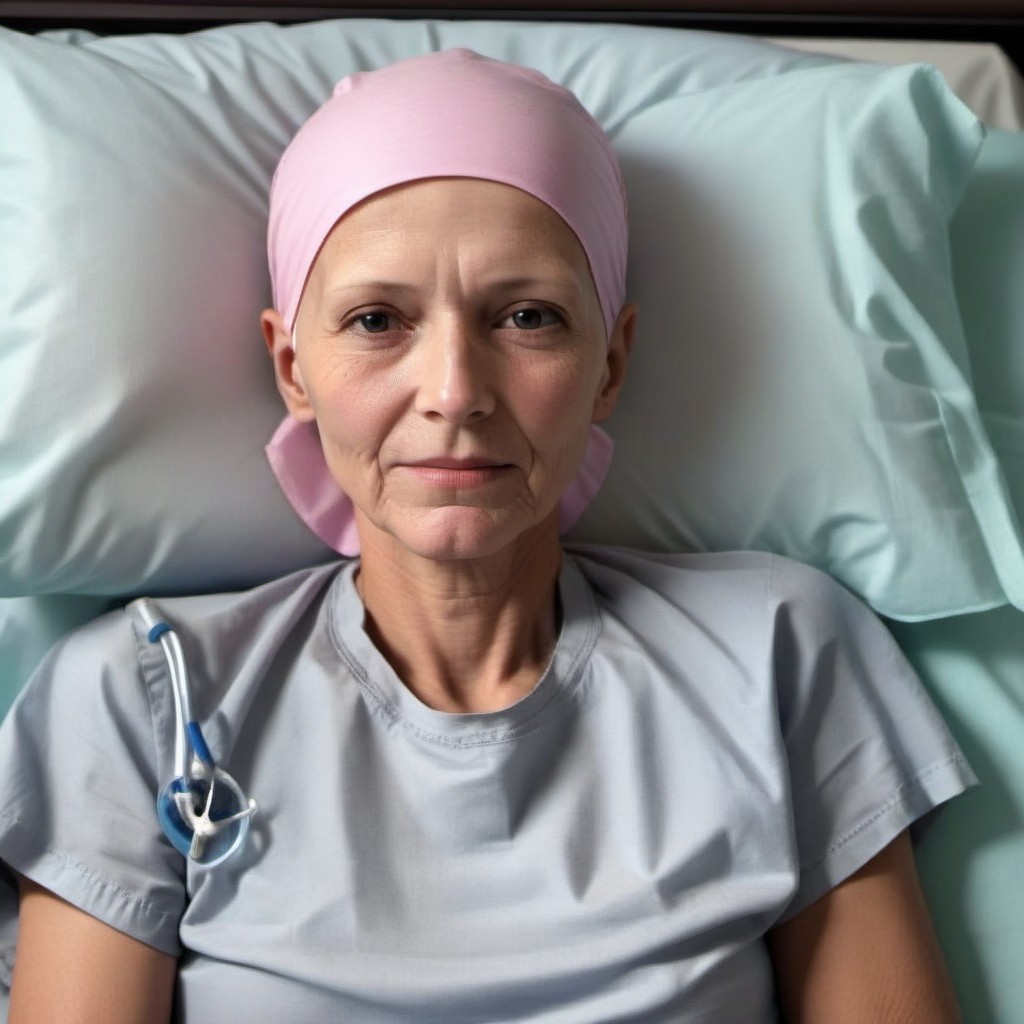

Supplement: Supplementary file 1 — Supplementary file1 (ZIP 11162 KB) [file 11764_2025_1760_MOESM1_ESM.zip › Data Images/cancer patient/Stable Diffusion/131.jpg]

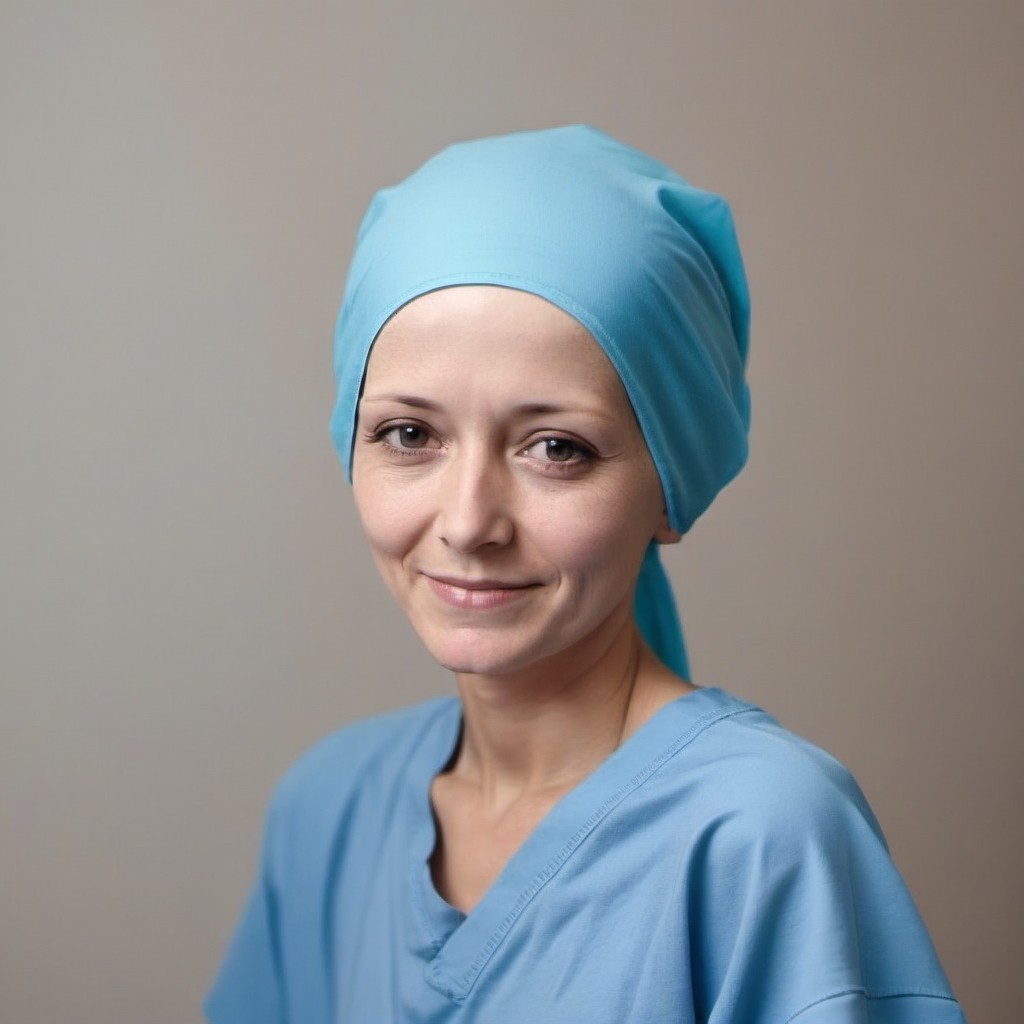

Supplement: Supplementary file 1 — Supplementary file1 (ZIP 11162 KB) [file 11764_2025_1760_MOESM1_ESM.zip › Data Images/cancer patient/Stable Diffusion/132.jpg]

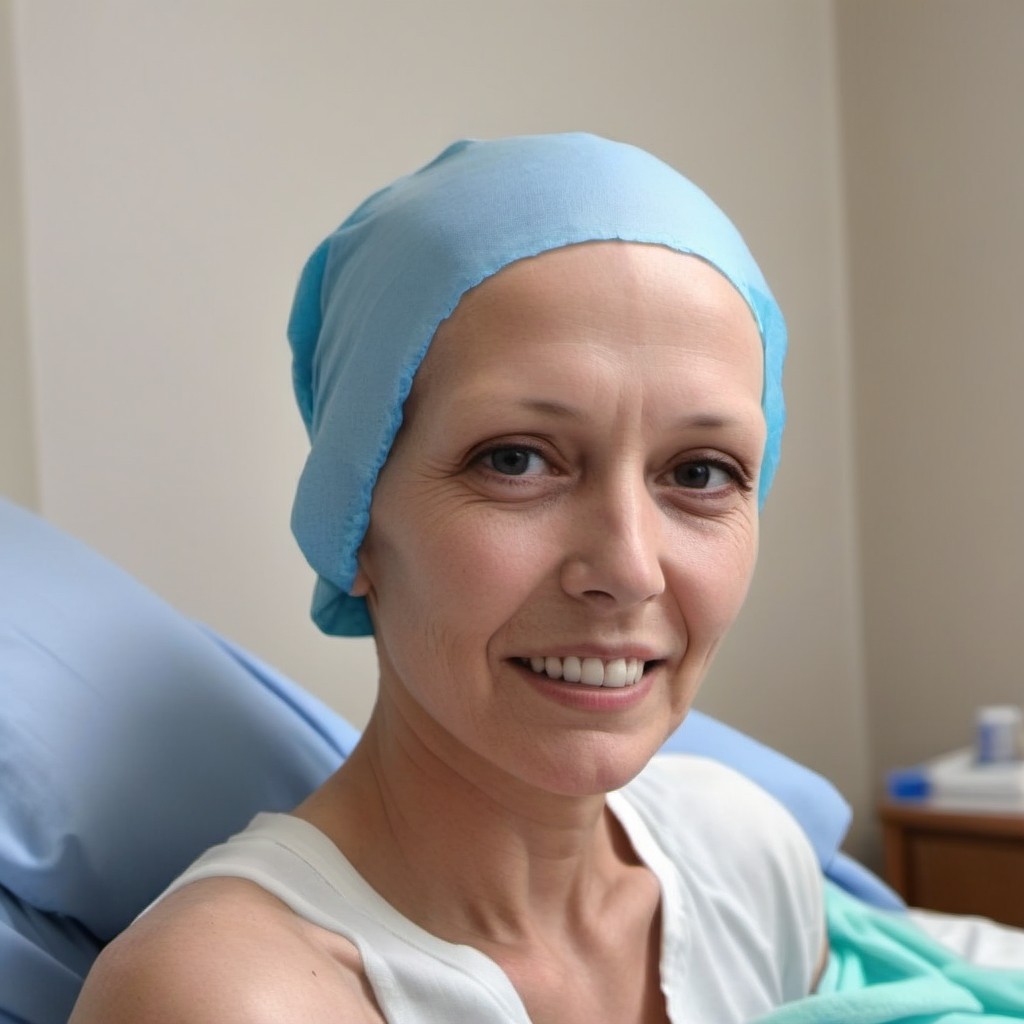

Supplement: Supplementary file 1 — Supplementary file1 (ZIP 11162 KB) [file 11764_2025_1760_MOESM1_ESM.zip › Data Images/cancer patient/Stable Diffusion/133.jpg]

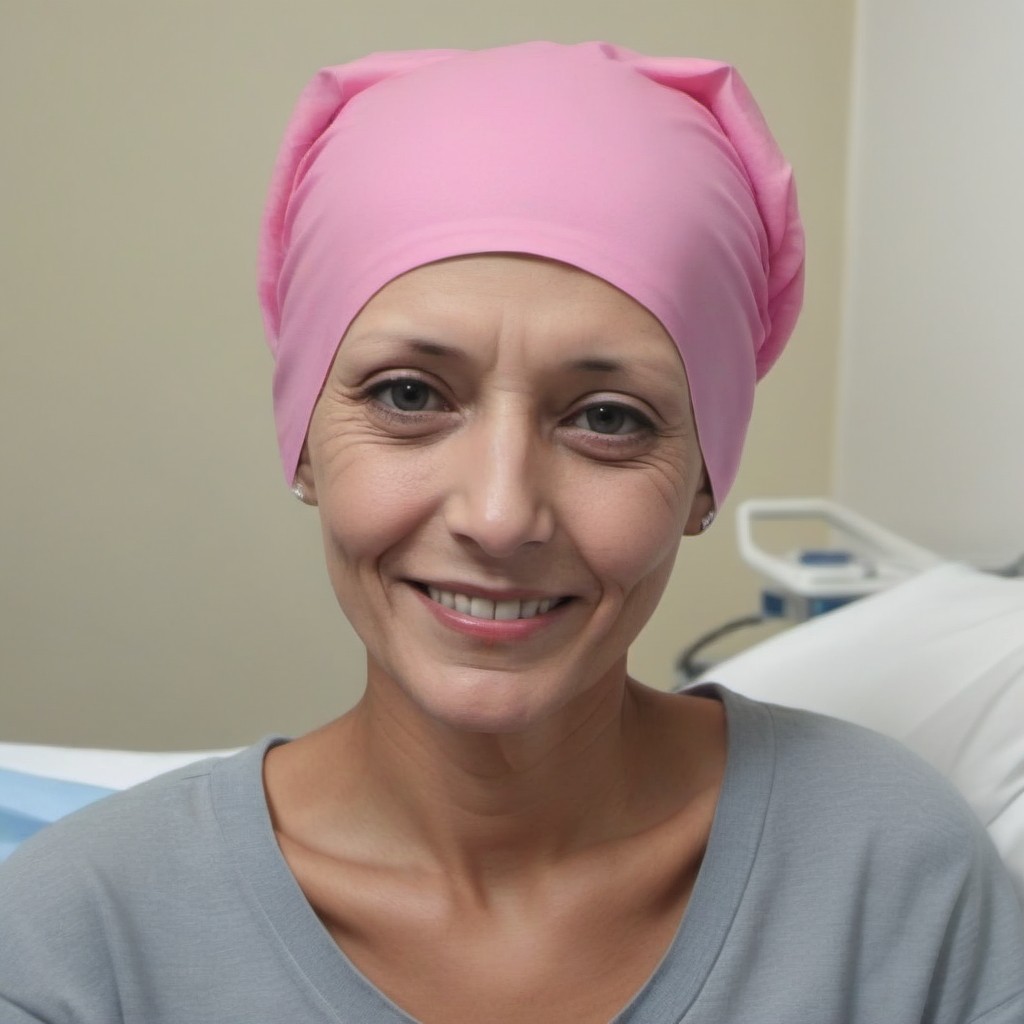

Supplement: Supplementary file 1 — Supplementary file1 (ZIP 11162 KB) [file 11764_2025_1760_MOESM1_ESM.zip › Data Images/cancer patient/Stable Diffusion/134.jpg]

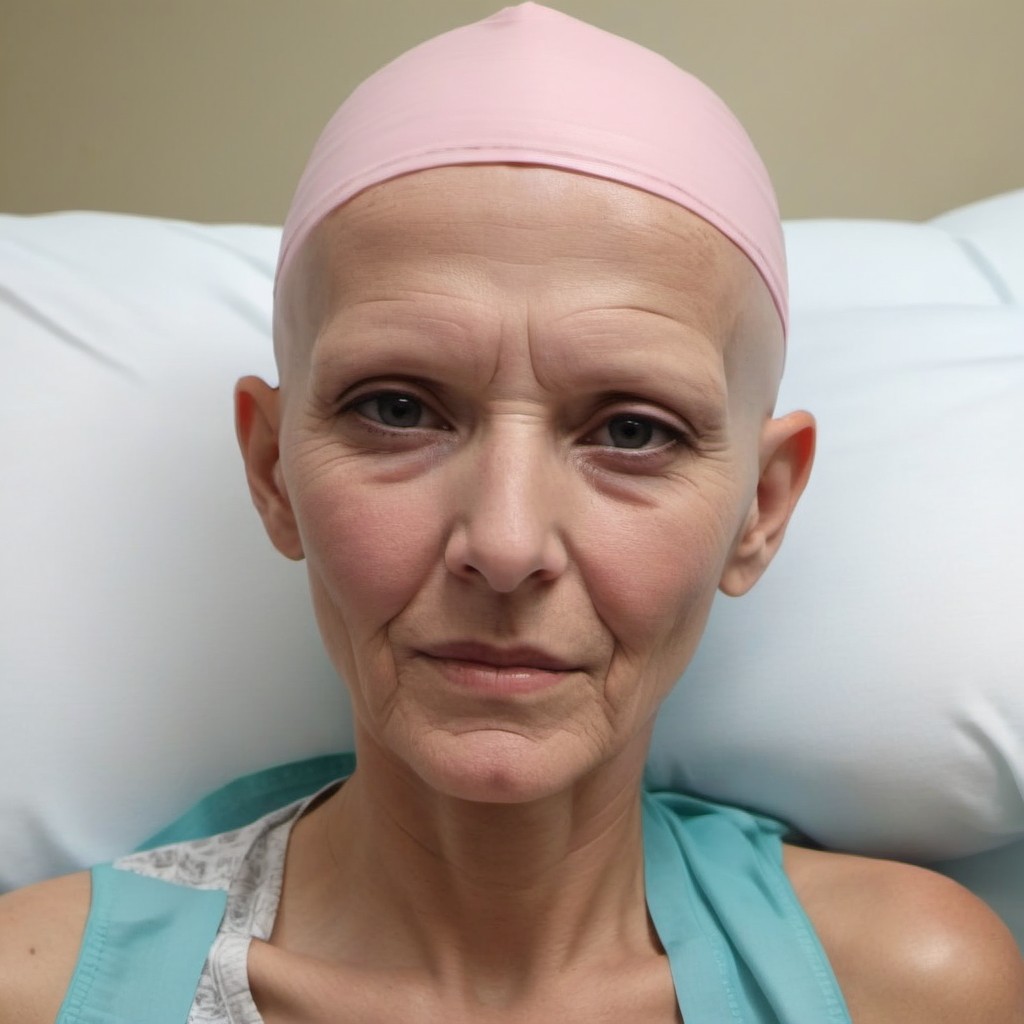

Supplement: Supplementary file 1 — Supplementary file1 (ZIP 11162 KB) [file 11764_2025_1760_MOESM1_ESM.zip › Data Images/cancer patient/Stable Diffusion/135.jpg]

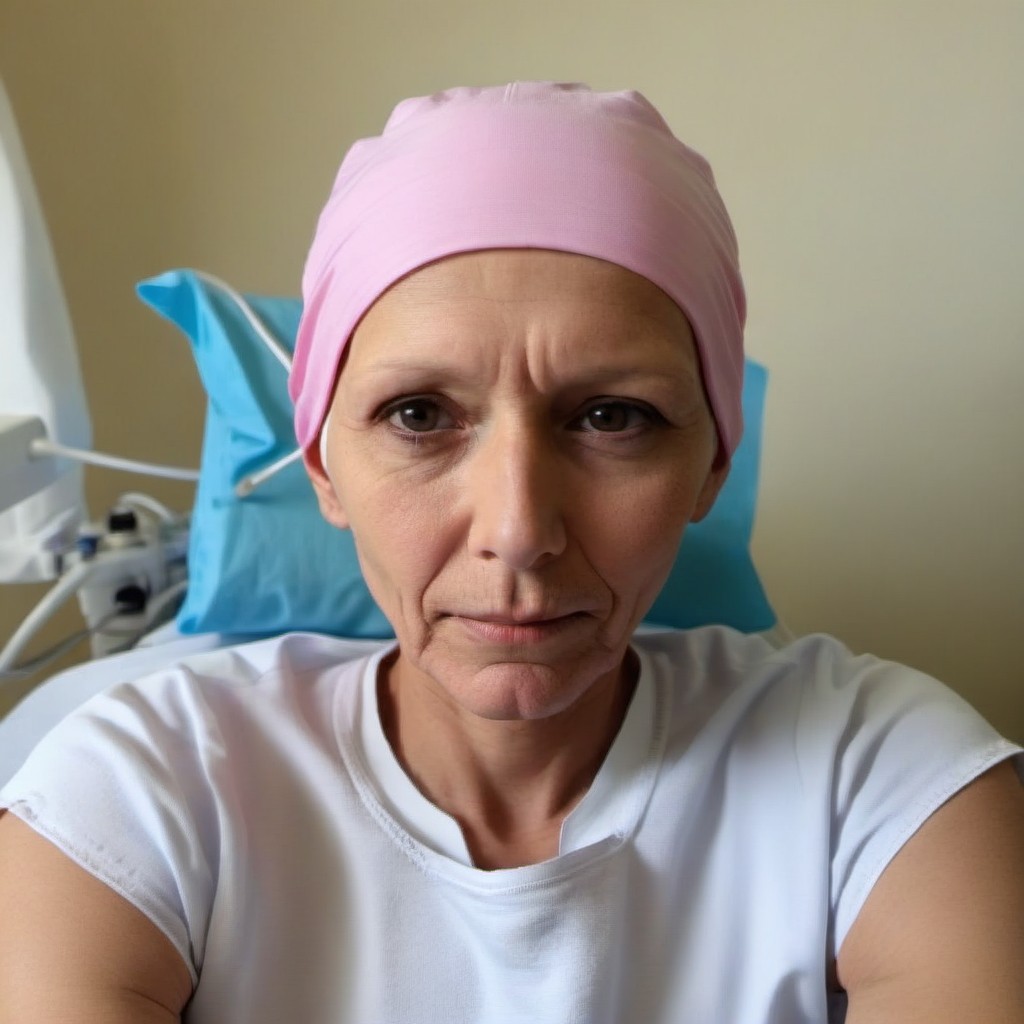

Supplement: Supplementary file 1 — Supplementary file1 (ZIP 11162 KB) [file 11764_2025_1760_MOESM1_ESM.zip › Data Images/cancer patient/Stable Diffusion/136.jpg]

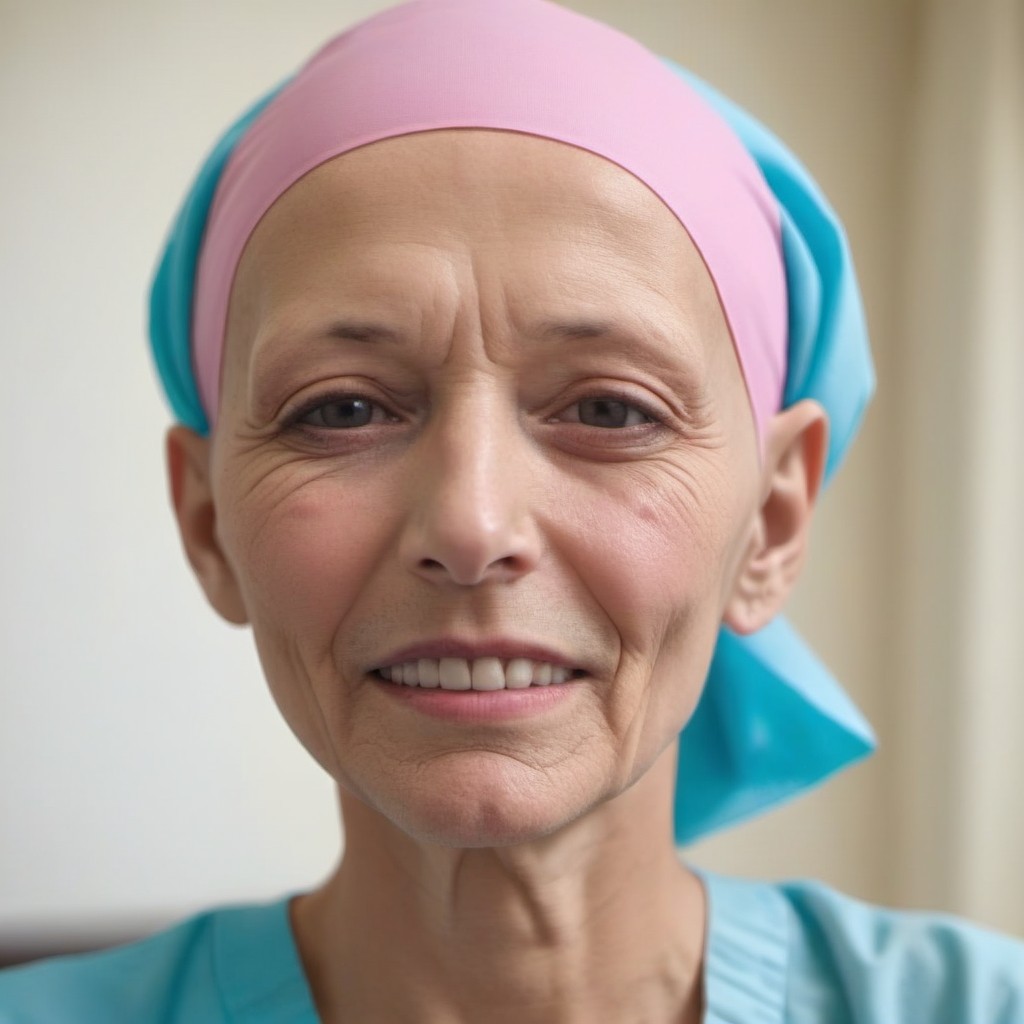

Supplement: Supplementary file 1 — Supplementary file1 (ZIP 11162 KB) [file 11764_2025_1760_MOESM1_ESM.zip › Data Images/cancer patient/Stable Diffusion/137.jpg]

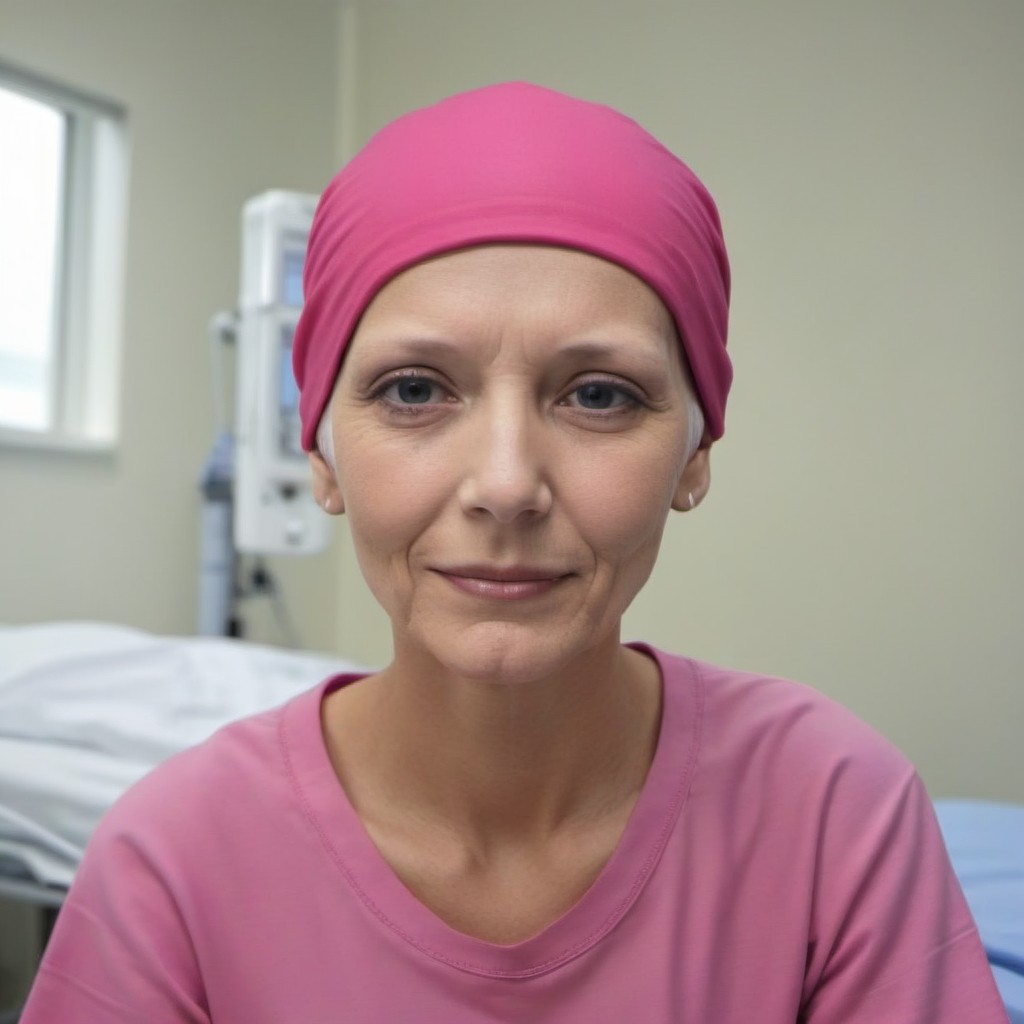

Supplement: Supplementary file 1 — Supplementary file1 (ZIP 11162 KB) [file 11764_2025_1760_MOESM1_ESM.zip › Data Images/cancer patient/Stable Diffusion/138.jpg]

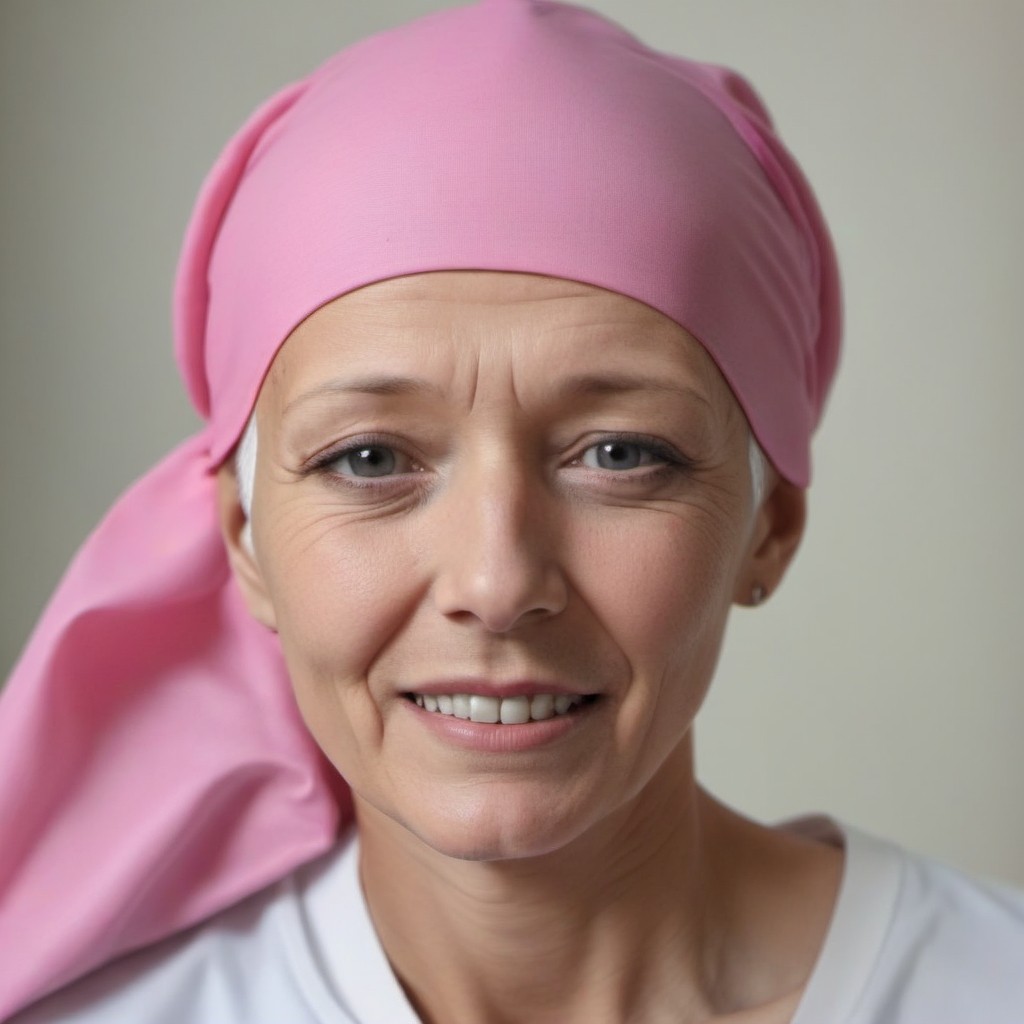

Supplement: Supplementary file 1 — Supplementary file1 (ZIP 11162 KB) [file 11764_2025_1760_MOESM1_ESM.zip › Data Images/cancer patient/Stable Diffusion/139.jpg]

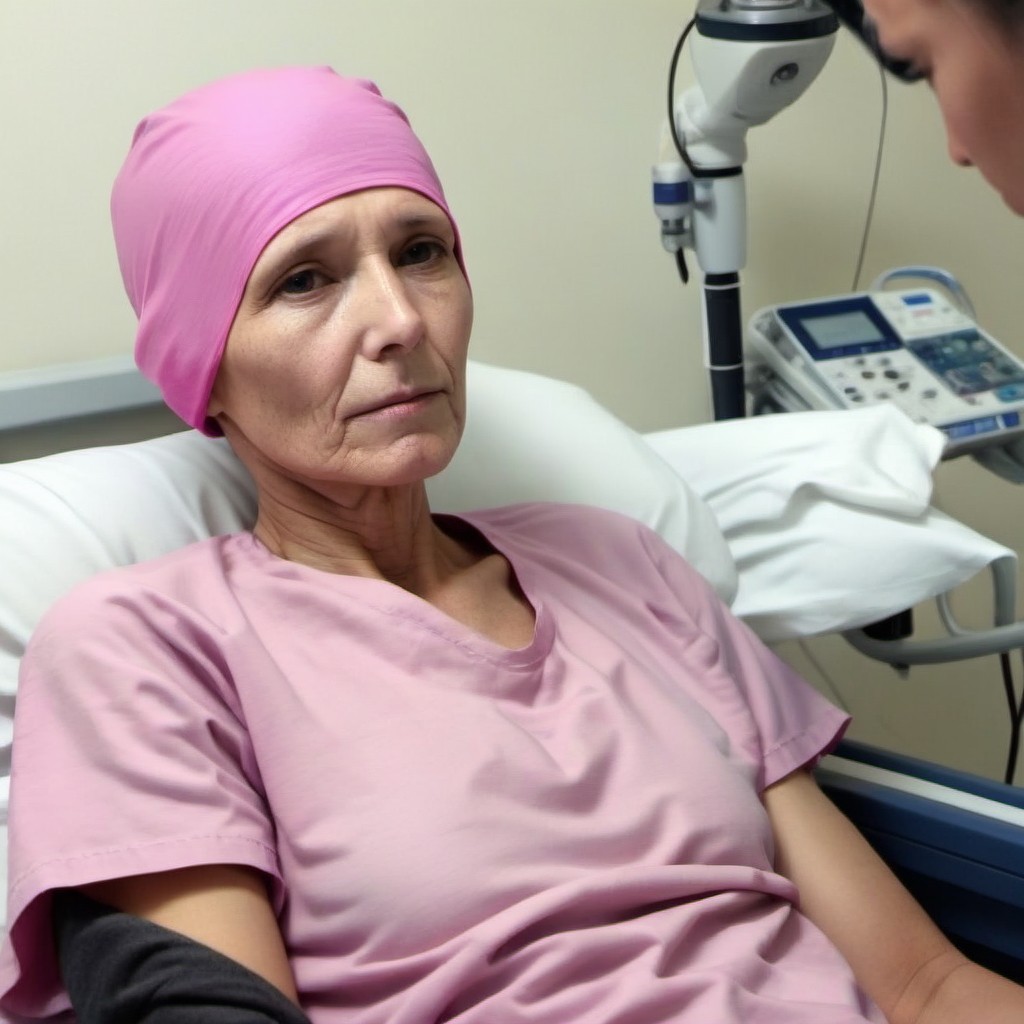

Supplement: Supplementary file 1 — Supplementary file1 (ZIP 11162 KB) [file 11764_2025_1760_MOESM1_ESM.zip › Data Images/cancer patient/Stable Diffusion/140.jpg]

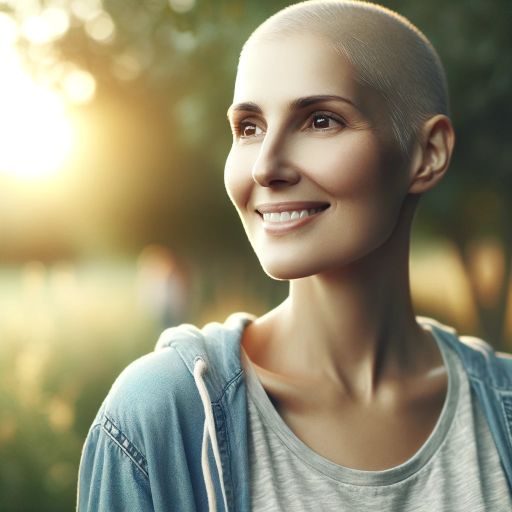

Supplement: Supplementary file 1 — Supplementary file1 (ZIP 11162 KB) [file 11764_2025_1760_MOESM1_ESM.zip › Data Images/cancer survivor/ChatGPT/381.jpg]

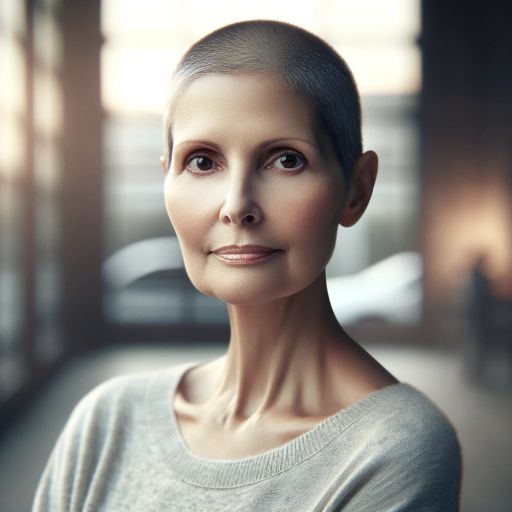

Supplement: Supplementary file 1 — Supplementary file1 (ZIP 11162 KB) [file 11764_2025_1760_MOESM1_ESM.zip › Data Images/cancer survivor/ChatGPT/382.jpg]

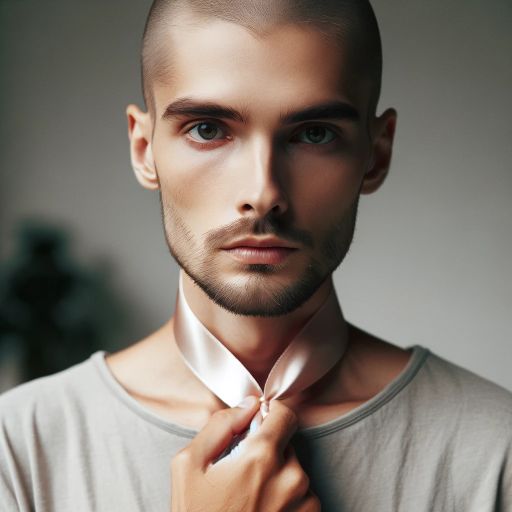

Supplement: Supplementary file 1 — Supplementary file1 (ZIP 11162 KB) [file 11764_2025_1760_MOESM1_ESM.zip › Data Images/cancer survivor/ChatGPT/383.jpg]

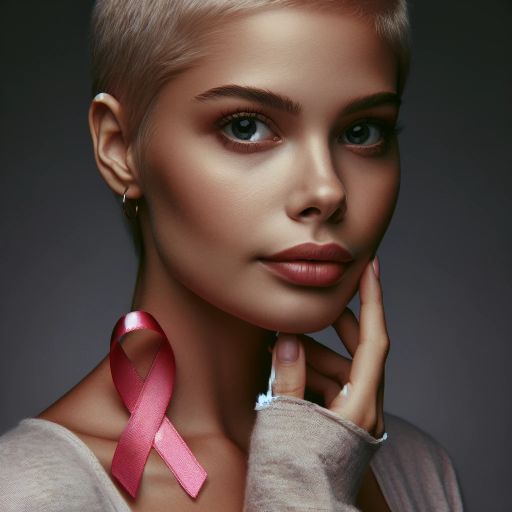

Supplement: Supplementary file 1 — Supplementary file1 (ZIP 11162 KB) [file 11764_2025_1760_MOESM1_ESM.zip › Data Images/cancer survivor/ChatGPT/384.jpg]

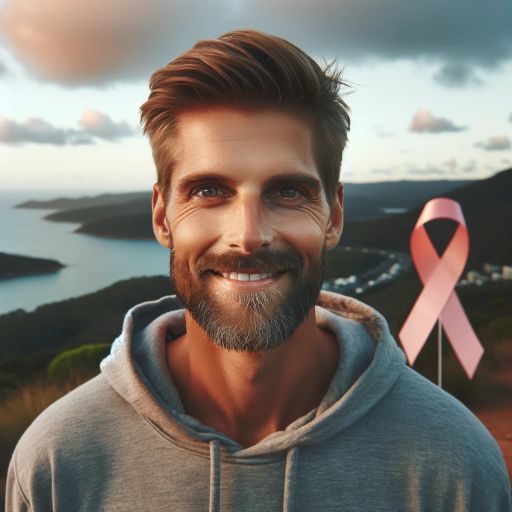

Supplement: Supplementary file 1 — Supplementary file1 (ZIP 11162 KB) [file 11764_2025_1760_MOESM1_ESM.zip › Data Images/cancer survivor/ChatGPT/385.jpg]

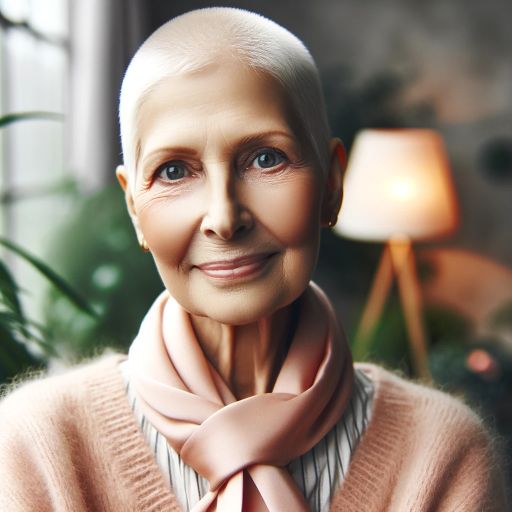

Supplement: Supplementary file 1 — Supplementary file1 (ZIP 11162 KB) [file 11764_2025_1760_MOESM1_ESM.zip › Data Images/cancer survivor/ChatGPT/386.jpg]

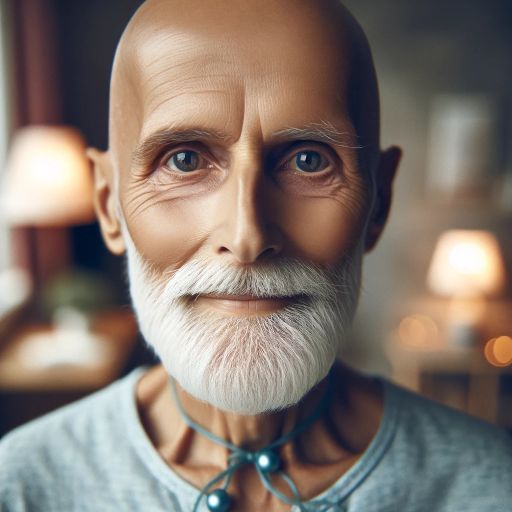

Supplement: Supplementary file 1 — Supplementary file1 (ZIP 11162 KB) [file 11764_2025_1760_MOESM1_ESM.zip › Data Images/cancer survivor/ChatGPT/387.jpg]

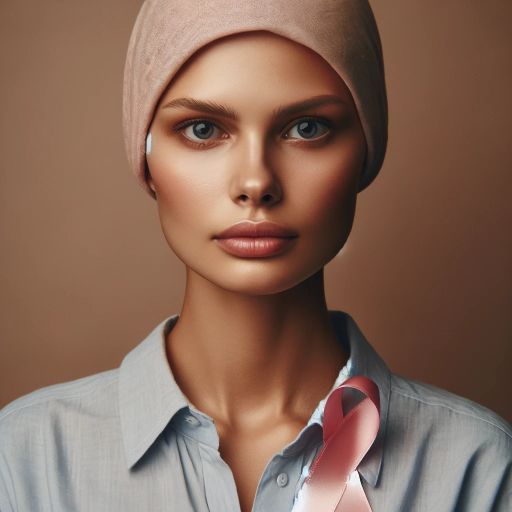

Supplement: Supplementary file 1 — Supplementary file1 (ZIP 11162 KB) [file 11764_2025_1760_MOESM1_ESM.zip › Data Images/cancer survivor/ChatGPT/388.jpg]

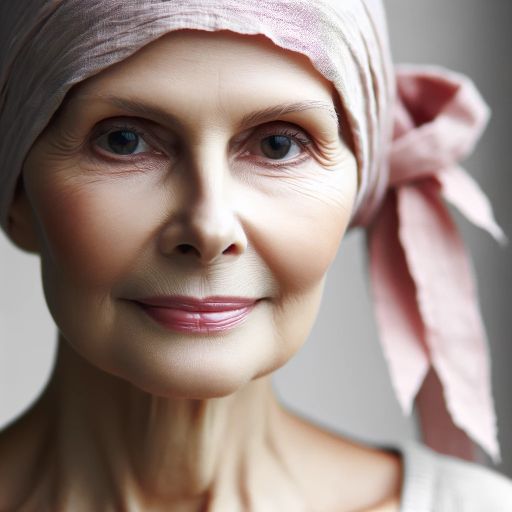

Supplement: Supplementary file 1 — Supplementary file1 (ZIP 11162 KB) [file 11764_2025_1760_MOESM1_ESM.zip › Data Images/cancer survivor/ChatGPT/389.jpg]

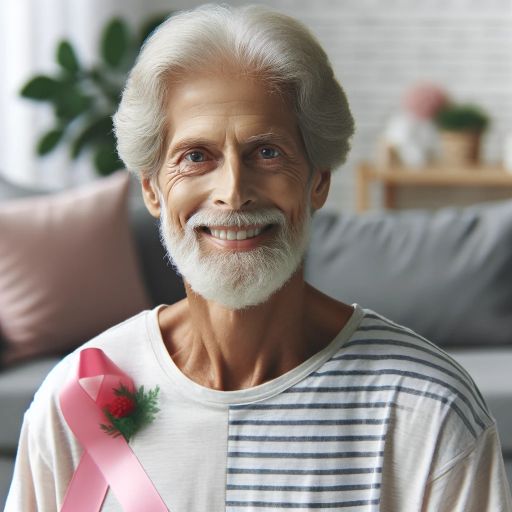

Supplement: Supplementary file 1 — Supplementary file1 (ZIP 11162 KB) [file 11764_2025_1760_MOESM1_ESM.zip › Data Images/cancer survivor/ChatGPT/390.jpg]

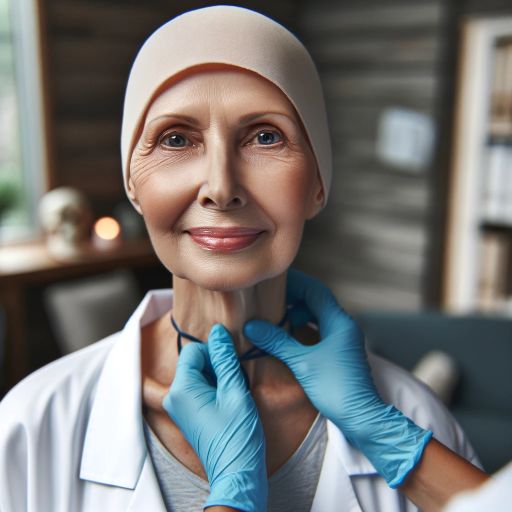

Supplement: Supplementary file 1 — Supplementary file1 (ZIP 11162 KB) [file 11764_2025_1760_MOESM1_ESM.zip › Data Images/cancer survivor/ChatGPT/391.jpg]

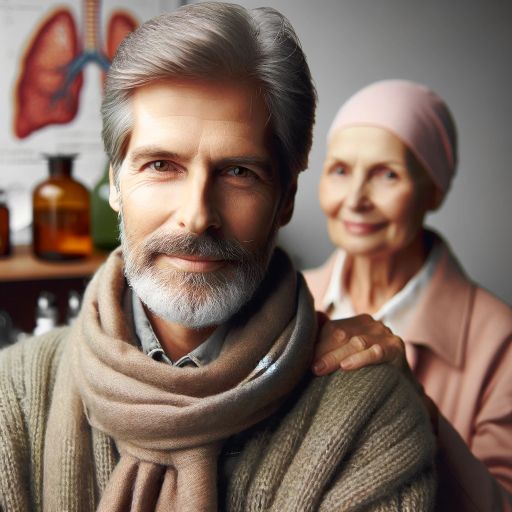

Supplement: Supplementary file 1 — Supplementary file1 (ZIP 11162 KB) [file 11764_2025_1760_MOESM1_ESM.zip › Data Images/cancer survivor/ChatGPT/392.jpg]

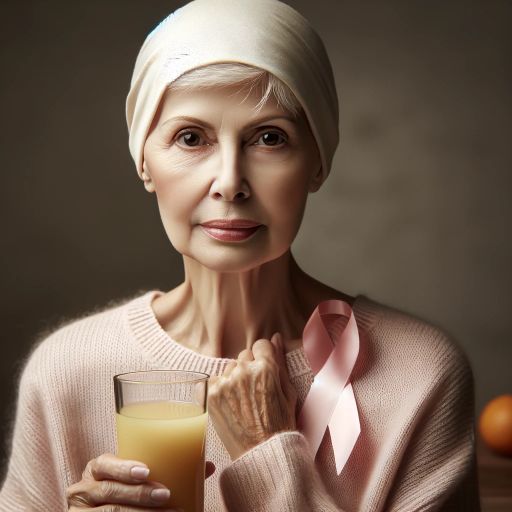

Supplement: Supplementary file 1 — Supplementary file1 (ZIP 11162 KB) [file 11764_2025_1760_MOESM1_ESM.zip › Data Images/cancer survivor/ChatGPT/393.jpg]

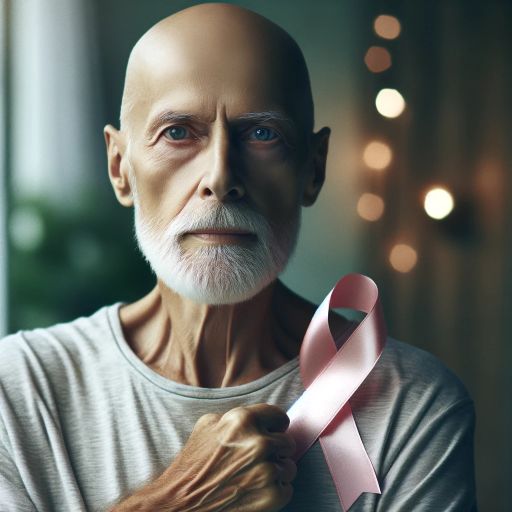

Supplement: Supplementary file 1 — Supplementary file1 (ZIP 11162 KB) [file 11764_2025_1760_MOESM1_ESM.zip › Data Images/cancer survivor/ChatGPT/394.jpg]

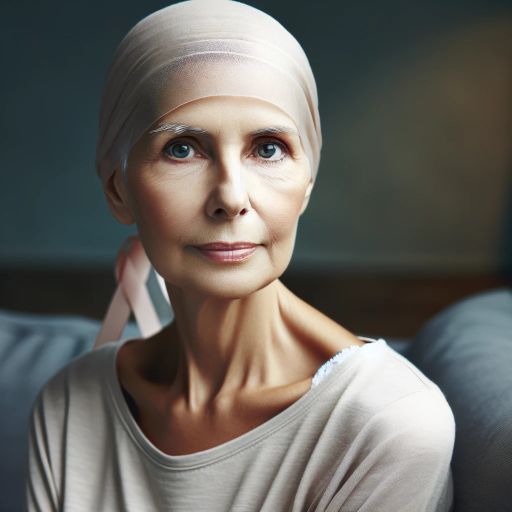

Supplement: Supplementary file 1 — Supplementary file1 (ZIP 11162 KB) [file 11764_2025_1760_MOESM1_ESM.zip › Data Images/cancer survivor/ChatGPT/395.jpg]

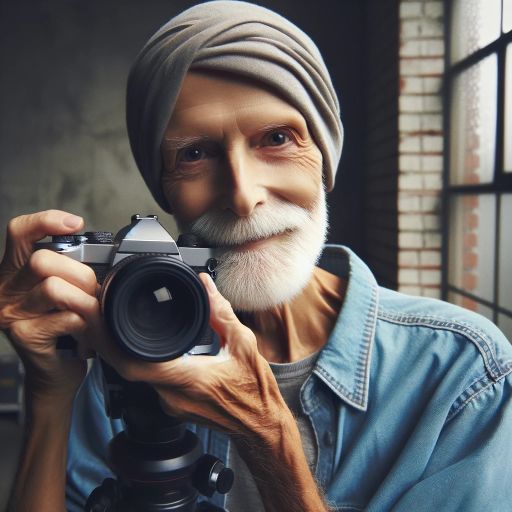

Supplement: Supplementary file 1 — Supplementary file1 (ZIP 11162 KB) [file 11764_2025_1760_MOESM1_ESM.zip › Data Images/cancer survivor/ChatGPT/396.jpg]

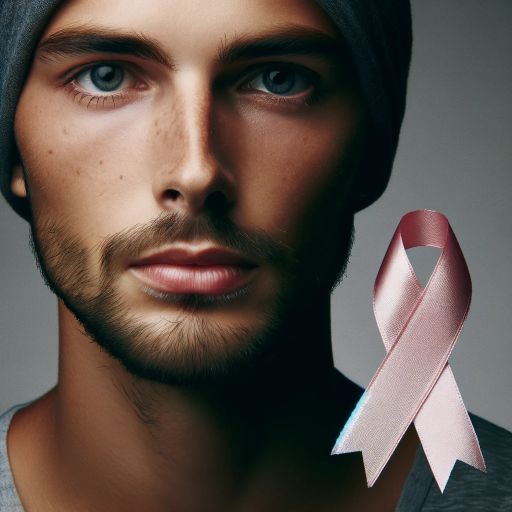

Supplement: Supplementary file 1 — Supplementary file1 (ZIP 11162 KB) [file 11764_2025_1760_MOESM1_ESM.zip › Data Images/cancer survivor/ChatGPT/397.jpg]

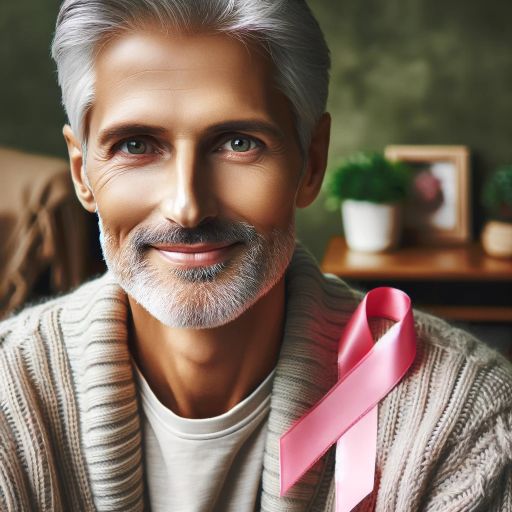

Supplement: Supplementary file 1 — Supplementary file1 (ZIP 11162 KB) [file 11764_2025_1760_MOESM1_ESM.zip › Data Images/cancer survivor/ChatGPT/398.jpg]

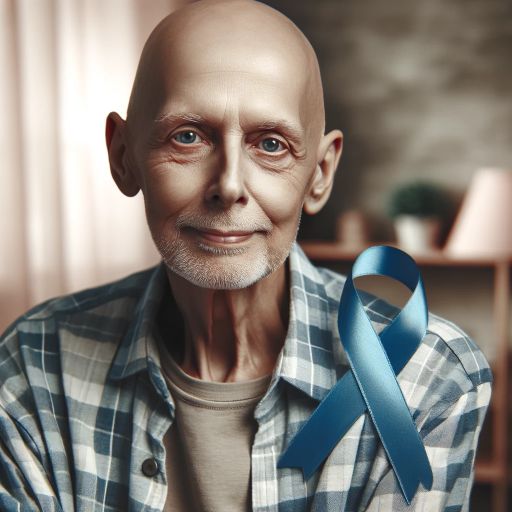

Supplement: Supplementary file 1 — Supplementary file1 (ZIP 11162 KB) [file 11764_2025_1760_MOESM1_ESM.zip › Data Images/cancer survivor/ChatGPT/399.jpg]

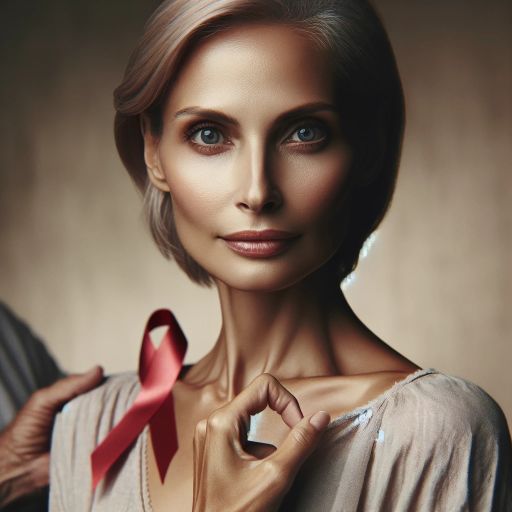

Supplement: Supplementary file 1 — Supplementary file1 (ZIP 11162 KB) [file 11764_2025_1760_MOESM1_ESM.zip › Data Images/cancer survivor/ChatGPT/400.jpg]
