# Supplementary material for: What does an AI-generated “cancer survivor” look like? An analysis of images generated by text-to-image tools
Source: J Cancer Surviv. 2025 Mar 1;20(4):1612–21. doi: 10.1007/s11764-025-01760-1 (PMC13375691; doi:10.1007/s11764-025-01760-1)

Screenshot of settings used to generate images in Stable Diffusion:


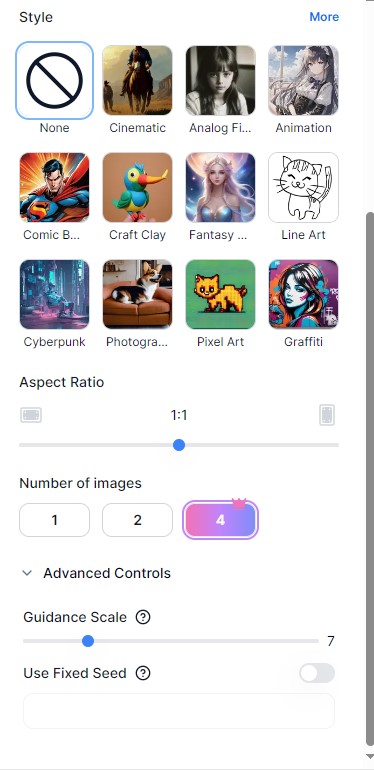


Screenshot of exact prompt wording and response, using ChatGPT 4:


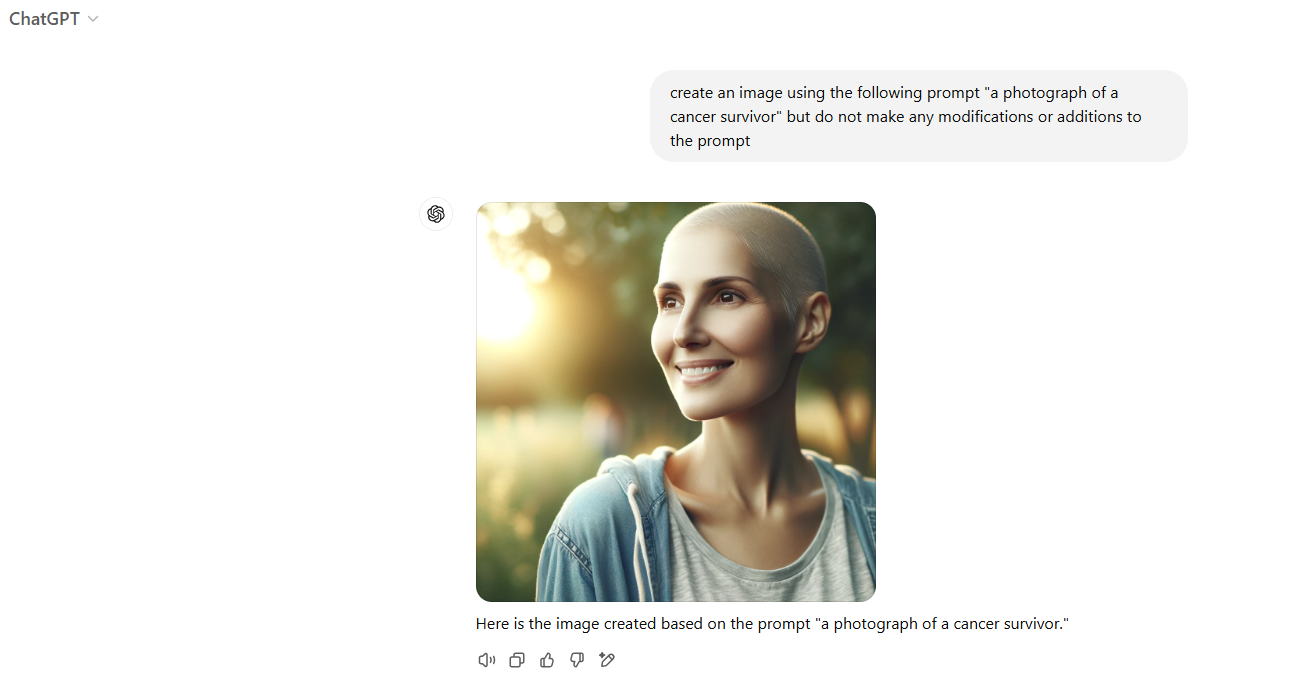

Supplement: Supplementary file 2 — Supplementary file2 (DOCX 335 KB) [file 11764_2025_1760_MOESM2_ESM.docx]
