# Supplementary material for: What does an AI-generated “cancer survivor” look like? An analysis of images generated by text-to-image tools
Source: J Cancer Surviv. 2025 Mar 1;20(4):1612–21. doi: 10.1007/s11764-025-01760-1 (PMC13375691; doi:10.1007/s11764-025-01760-1)
Supplement: Supplementary file 3 — Supplementary file3 (DOCX 17 KB) [file 11764_2025_1760_MOESM3_ESM.docx]

**Online Resource 3: Codebook**

1. Enter your coder initials (e.g., AG, NS): _______
2. Enter the number of the image you are coding: ________
3. Does the image contain a person?
   - Yes
   - No (if “no” is selected, skip to end of survey)

*If two or more people are in the image, code only for the focal person (person in the foreground) and add a note about there being multiple persons in Question 19.*

1. Race/ethnicity:
   - White
   - Non-White [specify below, if able; consider the following race categories: Black/African American; Hispanic/Latinx; American Indian or Alaska Native; Asian; Native Hawaiian or Other Pacific Islander]
   - Unclear [select if the focal person in the image does not look clearly white or non-white]
2. Skin tone:
   - Light [select for very fair skin tone]
   - Medium
   - Dark
3. Gender presentation:
   - Feminine
   - Masculine
   - Unclear
4. Age group:
   - Child/adolescent (<18)
   - Young adult (18-39)
   - Middle aged (>39 and <65)
   - Older adult (65+) [select if there are clear signs of advanced age, like a significant amount of grey hair or wrinkles]
5. Affect - facial expression:
   - Negative
   - Slightly negative
   - Neutral or mixed
   - Slightly positive
   - Positive
   - N/A (e.g., face is covered)
6. Presence of head covering:
   - Yes
   - No
7. Overall physical health appearance:
   - Very sick
   - Somewhat sick
   - Neither clearly sick, nor clearly healthy (neutral/can't tell)
   - Somewhat healthy
   - Very healthy
8. Features of person *[select all that apply]*:
   - Rash
   - In bed (sitting or lying down)/in a wheelchair
   - Absence of hair [look for total baldness; only code if head covering not present]
   - Wearing hospital gown (or similar medical clothing)
   - Other (e.g., frailty, sunken eyes, dark circles around eyes, etc. if these feature are significant) [specify] ____________
   - None of the above
9. Image setting:
   - Indoor - medical setting (e.g., hospital or clinic)
   - Indoor - not a medical setting (e.g., a bedroom)
   - Indoor - not clear
   - Outdoors
   - No identifiable background (e.g., solid background)
10. Presence of medical equipment/medical staff [consider the entire image]:
    - Medical equipment (including latex gloves, surgical face masks, stethoscopes, IVs)
    - Medical staff (someone other than the focal person portrayed as a medical provider)
    - None of the above
11. Presence of cancer ribbon (but not differently shaped bows):
    - Yes
    - No
12. Pink color significantly present:
    - Yes (any shade of pink)
    - No
    - N/A (picture is black and white)
13. Presence of anatomical elements (e.g., DNA, tumor, internal organs):
    - Yes
    - No
14. Level of photo realism:
    - Highly photorealistic
    - Somewhat photorealistic
    - Not very photorealistic
    - Not at all photorealistic (e.g., an animation/cartoon)
15. Obvious rendering errors present in the image, like extra hands or misspelled words:
    - Yes
    - No
16. Add any other details you think are worth mentioning and not captured above (e.g., presence of more than one individual in the image, nudity, person is portrayed as smoking).

________________________________________________________

1. Add any notes about your coding for this image (e.g., wasn’t sure how to code for Q7 because…).

________________________________________________________
