# Supplementary material for: What does an AI-generated “cancer survivor” look like? An analysis of images generated by text-to-image tools
Source: J Cancer Surviv. 2025 Mar 1;20(4):1612–21. doi: 10.1007/s11764-025-01760-1 (PMC13375691; doi:10.1007/s11764-025-01760-1)
Supplement: Supplementary file 4 — Supplementary file4 (DOCX 579 KB) [file 11764_2025_1760_MOESM4_ESM.docx]

**Online Resource 2: Examples of AI-generated images of cancer patients**

**
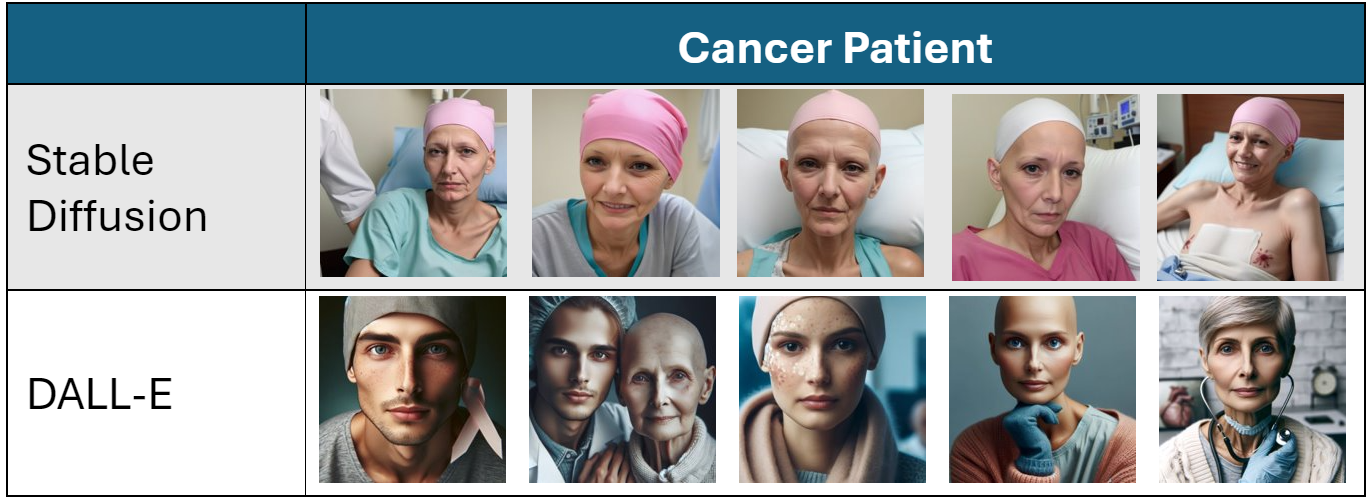
**

*Online Resource 2. Example images of cancer patients generated by Dall-E and Stable Diffusion. Each set of images was randomly sampled from the 40 outputs generated using each tool.*
